# Lipase-catalyzed esterification in water enabled by nanomicelles. Applications to 1-pot multi-step sequences†

**DOI:** 10.1039/d1sc05660c

**Published:** 2021-12-27

**Authors:** Vani Singhania, Margery Cortes-Clerget, Jade Dussart-Gautheret, Bhornrawin Akkachairin, Julie Yu, Nnamdi Akporji, Fabrice Gallou, Bruce H. Lipshutz

**Affiliations:** Department of Chemistry and Biochemistry, University of California Santa Barbara CA 93106 USA lipshutz@chem.ucsb.edu; Program on Chemical Biology, Chulabhorn Graduate Institute, Center of Excellence on Environmental Health and Toxicology (EHT), Ministry of Education 54 Kamphaeng Phet 6, Laksi Bangkok 10210 Thailand; Novartis Pharma AG CH-4057 Basel Switzerland

## Abstract

Esterification in an aqueous micellar medium is catalyzed by a commercially available lipase in the absence of any co-factors. The presence of only 2 wt% designer surfactant, TPGS-750-M, assists in a 100% selective enzymatic process in which only primary alcohols participate (in a 1 : 1 ratio with carboxylic acid). An unexpected finding is also disclosed where the simple additive, PhCF_3_ (1 equiv. *vs.* substrate), appears to significantly extend the scope of usable acid/alcohol combinations. Taken together, several chemo- and bio-catalyzed 1-pot, multi-step reactions can now be performed in water.

## Abstract 2

Esterification in an aqueous micellar medium is catalyzed by a commercially available lipase in the absence of any co-factors.

## Graphical abstract

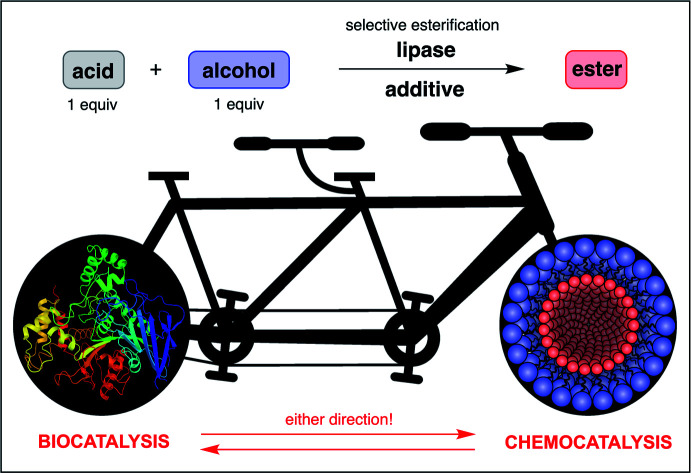

## Introduction

Textbook and related chemical methods continue to discuss the fundamental preparation of esters from acids and alcohols, typically performed in organic solvents, that require removal of water.^1^ Alternatively, biocatalytic processes, while offering mild conditions, high selectivity, and, in general, environmental friendliness, are typically used in aqueous buffered media.^2–6^ Hence, esterification *via* enzymatic catalysis in water involving lipase, esterase, *etc.*, where water is the by-product, seems counterintuitive.^7–10^ Special conditions (*e.g.*, the presence of an organic solvent, prior acid activation, membrane-bound or solid supports, *etc.*) are required to address this issue.^11,12^ Only earlier this year did a report appear, outside the food industry,^7,13^ describing an enzyme-catalyzed ester synthesis in aqueous alcoholic media.^14^

Given the importance of the ester linkage in so many industrial products, it seems remarkable that such a functional group has yet to be constructed *via* a straightforward biocatalytic-based esterification; that such green technology indicative of the options available to synthetic chemists in water, in the absence of any organic solvent, and in particular, dipolar aprotic choices including DMSO,^15^ has yet to be described. We now report that use of an economical, commercially available lipase can, indeed, catalyze esterification in water as the reaction medium, and do so using a 1 : 1 stoichiometric ratio of acid and alcohol. Moreover, these take place in the absence of any co-factor. The key to successful levels of conversion is the presence of the designer surfactant TPGS-750-M (dl-α-tocopherol methoxypolyethylene glycol succinate) in the reaction medium.^16^ Even further levels of conversion can be anticipated by the presence of a simple additive (1 equiv.), as disclosed herein. Applications of this new esterification, together with chemocatalysis, leads to unprecedented tandem, 1-pot processes illustrative of the potential for organic synthesis to be conducted in sequential fashion, all done in water.

## Results and discussion

Esterification reactions were tested using four commercially available lipases: those from *Candida rugosa*, *Rhizopus niveus*, *Rhizomucor miehei*, and *Burkholderia cepacia*, with *Rhizomucor miehei* providing the best results (Table 1).^17^ For optimization purposes, lipase-catalyzed esterification between partners valeric acid (1) and *n*-hexanol (2) was initially examined. While keeping the concentration of valeric acid at either 0.25 M or 0.50 M, varied amounts of hexanol were added at both global reaction concentrations and temperatures. At 0.25 M (entries 1–3), increasing the quantity of hexanol introduced to the reaction mixture maintained at 40 °C improved the extent of product conversion. Increasing the concentration to 0.50 M (entries 4–6), unfortunately afforded no observed improvement. However, lowering the temperature to 30 °C led to essentially full conversion, thereby allowing for use of the ideal ratio of 1 : 1 (entry 8). Neither further reduction in temperature to rt (entry 7) nor increase in reaction temperature to 50 °C (entry 9) gave comparable levels of conversion relative to those observed at 30 °C.

**Table 1:** Optimization of esterification in aqueous buffer + surfactant

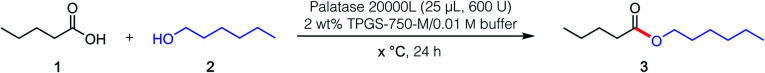				
Entry	Conc. [M]	Alcohol (equiv.)	Temp. (°C)	Conversion^a^ (%)
1	0.25	1	40	66
2	0.25	3	40	78
3	0.25	5	40	91
4	0.50	1	40	83
5	0.50	3	40	79
6	0.50	5	40	79
7	0.50	1	22^b^	74
**8**	**0.50**	**1**	**30**	**>99**
9	0.50	1	50	9

a Determined from crude NMR.

b rt.

**Scheme 1:**
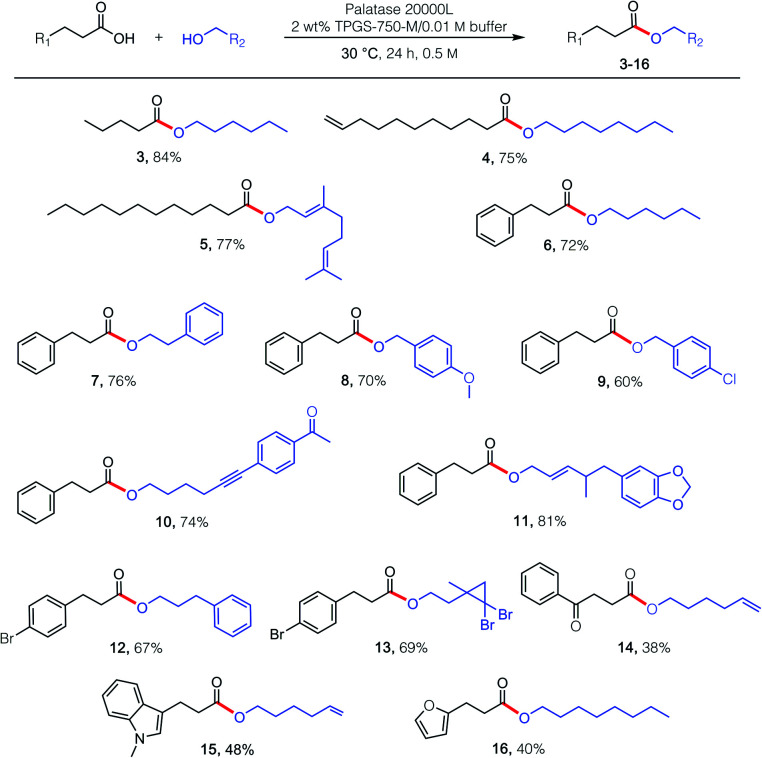
Substrate scope: lipase-catalyzed esterification in buffer under micellar conditions. Conditions: carboxylic acid (0.5 mmol; 1 equiv.), alcohol (0.5 mmol; 1 equiv.), *Rhizomucor miehei* enzyme (25 μL), 2 wt% TPGS-750-M in 0.01 M phosphate buffer solution (1 mL) at 30 °C for 24 h. Isolated yields reported.

Using these optimized conditions (*i.e.*, 1 : 1 acid : alcohol, 30 °C), several esterification reactions were examined, catalyzed by lipase derived from *Rhizomucor miehei* (Scheme 1; commercially available; see the ESI†). Given an enzyme's typical preference for certain structural features associated with reaction partners, as witnessed with carboxylic acids, somewhat greater flexibility was observed in terms of the alcohol that participated in the esterification process. While products 3–16 reflect these enzymatic requirements, they are solely representative of the possibilities for lipases, perhaps in general, to effect esterification in water. Unexpectedly, while the presence of 2 wt% TPGS-750-M (*i.e.*, 20 mg mL^−1^ of water) in the buffered aqueous medium proved beneficial in most cases, increasing the amount to either 4 or 6 wt%, unlike that observed with KRED,^18^ led to no further benefit. While these esterifications in water seem unexpected, the key to success may be the presence of the micelles. Hence, the water-insoluble product esters likely locate within their inner cores, which feature a purely hydrophobic pocket. Thus, opportunities for competitive hydrolysis by water are negated due to this “reservoir” effect.^18^

**Fig. 1:**
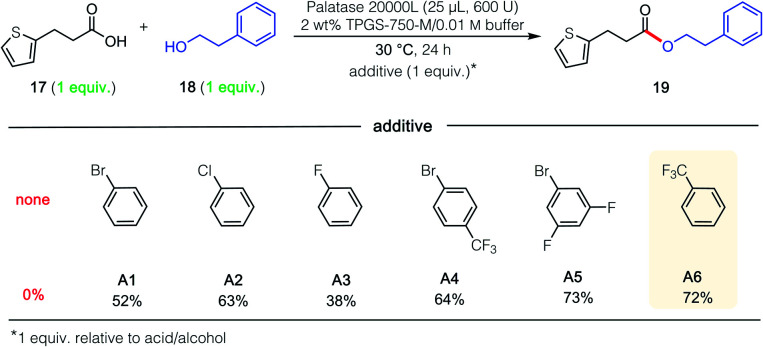
Screening the effect of additives on an esterification reaction.

**Scheme 2:**
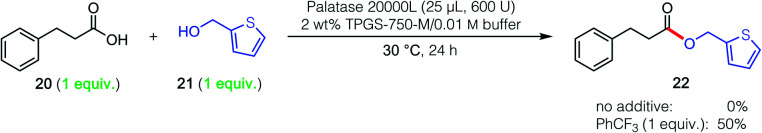
Impact of PhCF_3_ on inverted substrate pair (*i.e.*, *vs.* reaction pair in Fig. 1).

In attempts to extend these lipase-catalyzed esterification reactions to a broader range of heteroaromatic ring-containing partners (*i.e.*, beyond those in 15 and 16; Scheme 1), educt 3-(2-thiophenyl)propionic acid (17; Fig. 1), the analog of 3-phenylpropionic acid (Scheme 1, R_1_ = Ph) was examined, along with phenethyl alcohol (18), which had successfully led to product 7. In the event, and notwithstanding the thiophene's identical distance from the reactive carboxyl site, esterification was completely shut down (Fig. 1; 0% yield). Unexpectedly, therefore, when acid 17 was admixed with either the phenyl- or *p*-bromophenylpropionic acid (see 20 in Scheme 2) and the same alcohol (18), the major ester formed contained the thiophene subunit, product 19! This finding initiated a search for an additive that, ultimately, enabled esterification of 17 to 19 in 72% that was otherwise completely inhibited (notwithstanding the presence of the surfactant). This phenomenon is unlikely to be a simple solvent effect, since common solvents like methylene chloride (no conversion), hexanes (25% yield), cyclohexane (29% yield) and toluene (50% yield) were comparatively ineffective.^19^ Also as shown in Fig. 1, among the additives investigated (A1–A6), trifluoromethylbenzene (PhCF_3_; A6) was selected given its effectiveness, commercial availability, non-chlorinated status, and attractive economics.

The unpredictable yet very intriguing impact of the additive on the inverted combination of reaction partners (*i.e.*, phenylpropanoic acid, 20 and 2-thiophenemethanol, 21) was also studied. In the event, similar results were found where, in the absence of additive, no conversion to ester 22 was observed. However, when PhCF_3_ (1 equiv.) was in the aqueous reaction medium, a remarkable 50% yield was obtained (Scheme 2).

Further studies were carried out on the effect induced by this additive (PhCF_3_) on the lipase derived from *Rhizomucor miehei*,^20^ focusing on several control reactions (Table 2). The highest yield was observed in the presence of one equivalent of additive (entry 3); using half this amount dramatically lowered the extent of conversion (entry 2). Using PhCF_3_ in excess (entry 4) led to a decrease in yield to 57%. Neither the micellar medium alone (entry 1) nor just the aqueous buffer (entry 5) is capable of mediating the intended reaction, a strong indication that acid 17 by itself, as observed previously (*vide supra*), is not an acceptable reaction partner for this lipase-mediated esterification.

**Table 2:** Impact of reaction variables (see reaction in Fig. 1) of additive, PhCF_3_

Entry	Solvent^a^	Additive^b^ (equiv.)	Yield^c^ (%)
1	2 wt% TPGS-750-M/0.01 M buffer	—	0
2	2 wt% TPGS-750-M/0.01 M buffer	PhCF_3_ (0.5)	14
**3**	**2 wt% TPGS-750-M/0.01 M buffer**	**PhCF**_**3**_ **(1)**	**72**
4	2 wt% TPGS-750-M/0.01 M buffer	PhCF_3_ (2)	57
5	0.01 M buffer (no surfactant)	PhCF_3_ (1)	0

a 1 mL of solvent used for 0.5 M global concentration.

b One equiv. of this additive represents *ca.* 6% of the total reaction volume.

c Isolated yield of 19.

**Fig. 2:**
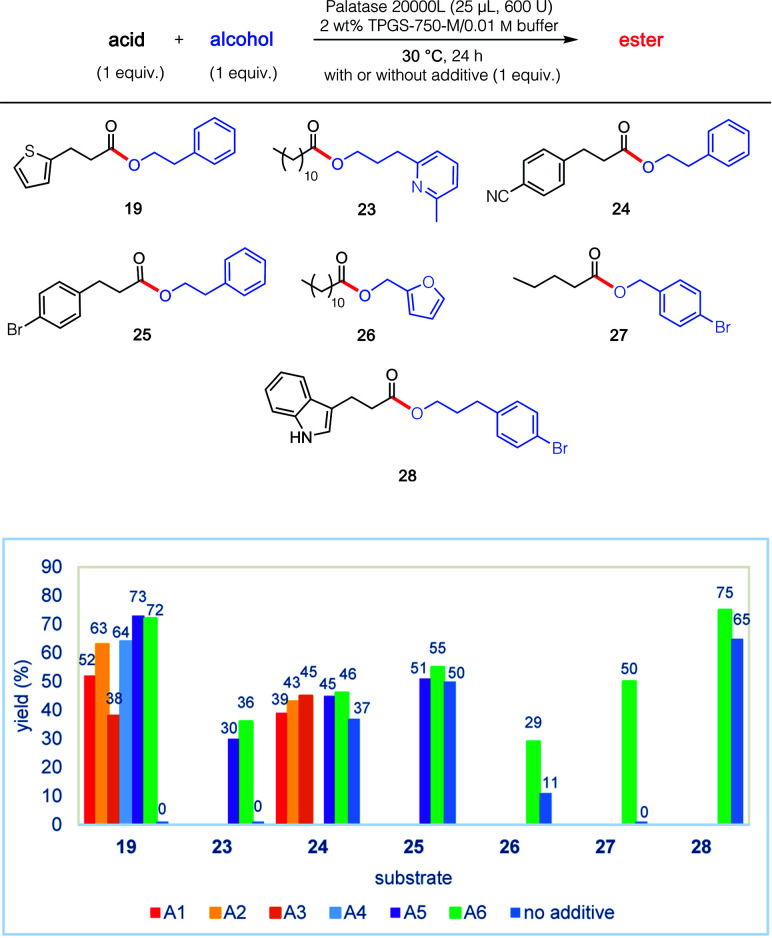
Impact of various additives (see Fig. 1) on lipase-catalysed esterification reactions.

The remarkable impact of the combination of an additive A1–A6, together with the buffer and surfactant in the pot, was further investigated on other substrates 19, and 23–28 (Fig. 2). Thus, in addition to the initial discovery involving esterification to product 19, the isolated yield using PhCF_3_ of esters 23 and 27 increased from 0 to 36% and from 0 to 50%, respectively. And while more modest enhancements in yields were observed for products 24–26 and 28, the overall net positive trend using PhCF_3_ is clear.

The manner in which PhCF_3_ influences enzymatic esterification may be due to a positive allosteric regulation, altering the enzymatic cavity. Typically, however, this phenomenon relies on far more functionalized molecules;^21^ indeed, the overall dramatic effect mediated by such a simple additive as PhCF_3_ appears to be unprecedented, perhaps suggesting that enzymatic modification leading to greater substrate tolerance may not require the types of modulators in current use. Another explanation may be the direct alteration of the entrance to the enzymatic pocket, thereby adding a variable element of “promiscuity” to the level of acceptance associated with its “natural” structural features.^22^ Yet another explanation is that this additive may be providing a hydrophobic layer that alters the extent of lid opening at the enzymatic site.^23^ While a more definitive analysis as to which role is operative awaits further scrutiny, these observations may be indicative of future discoveries perhaps applicable to other enzymatic arrays, thereby augmenting the already huge potential of bio-catalytic processes in organic synthesis.

**Fig. 3:**
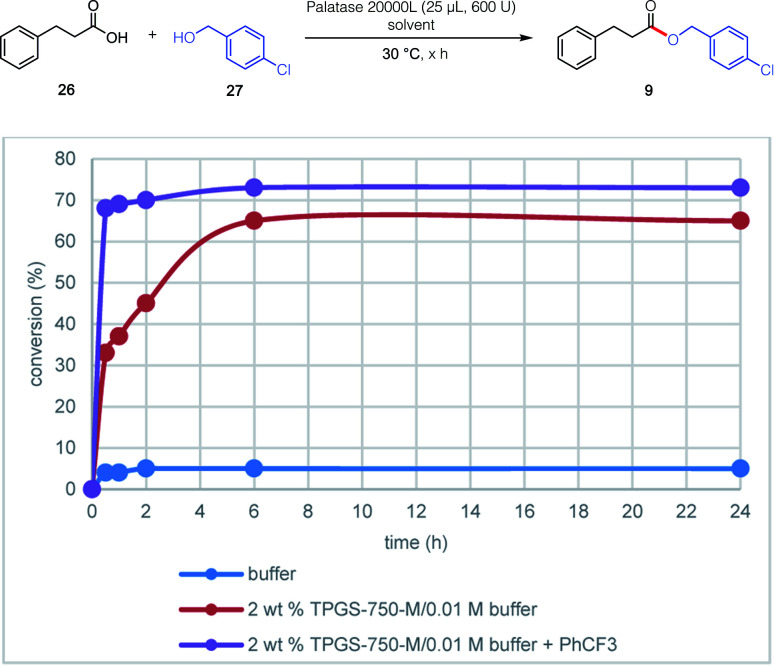
Impact of surfactant and additive on the representative esterification between 20 and 29.

Contributions by the surfactant and additive could be individually evaluated based on the “reservoir effect” first discovered in enzymatic ketone reductions (KREDs).^18^ This phenomenon was also observed in the lipase-catalyzed esterification between 3-phenylpropanoic acid 20 and (4-chlorophenyl)methanol 29 leading to ester 9 (Fig. 3). Thus, while esterification in only buffer did not exceed 5% after a 24 hour period (reflecting commonly observed enzymatic inhibition), the presence of only 2 wt% TPGS-750-M increased the level of conversion to 65%. The same reaction with added PhCF_3_ (A6, 1 equiv.) further raised the conversion to 73%. Noteworthy, however, was the dramatic increase in reaction rate, reaching 70% after only two hours.

**Scheme 3:**
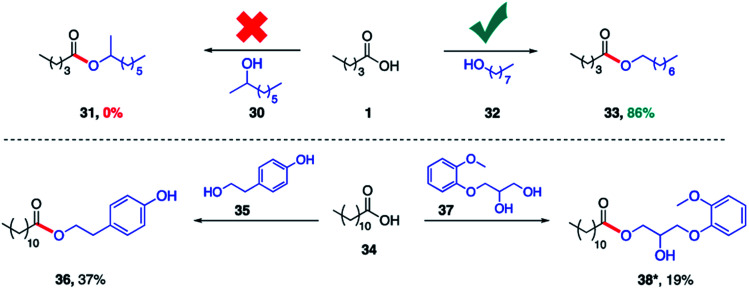
Selective esterification of a primary alcohols catalyzed by lipase. Conditions: *Rhizomucor miehei* enzyme (25 μL), 2 wt% TPGS-750-M/0.01 M buffer solution (1 mL) at 30 °C for 24 h. Only one product observed. Isolated yields reported. *Trace conversion in the absence of PhCF_3_.

The same lipase (*Rhizomucor miehei*) was also found to selectively catalyze esterification with primary alcohols to the complete exclusion of secondary alcohols. Hence, while valeric acid (1) was converted in the presence of 1-octanol (32) to product 33 (86%), identical treatment with 2-octanol (30) afforded none of the corresponding ester 31 (Scheme 3). This specificity prevails even when the alcohol functional groups are present within the same molecule. Thus, irrespective of the connectivity of the alcohol on a sp^2^ carbon (phenol, 35) or an sp^3^ carbon (secondary alcohol, 37), the primary alcohol is esterified exclusively. The same selective outcome favoring esterification of a primary alcohol is observed in the presence of PhCF_3_ (1 equiv.).

With water being the common denominator associated with chemo- and bio-catalysis, virtually unlimited opportunities are available for combining each in 1-pot sequences. The benefits to be realized from such a synthetic strategy include not only minimizing workups and thereby, waste creation, but also time invested (“time economy”),^24^ and processing (“pot economy”).^25^ Importantly, while maintaining global concentrations that typically range between 0.25 and 1 M,^18^ the sequence of reactions involving chemo- or bio-catalysis can be varied (*vide infra*).

**Scheme 4:**
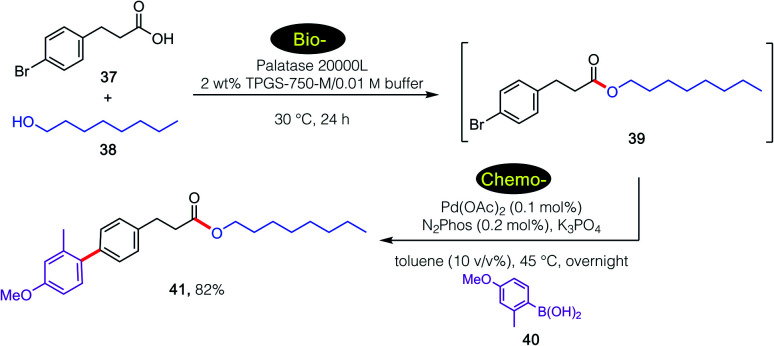
A 1-pot, 2-step bio-/chemo-catalysis sequence in an aqueous micellar medium.

For example, a sequence involving bio- followed by chemo-catalysis is shown in Scheme 4. Lipase-catalyzed esterification of 39 with 40 leads to product 41. Without isolation, and given the presence of the aryl bromide being amenable to a ppm Pd-catalyzed Suzuki–Miyaura coupling^26^ involving arylboronic acid 42, final product 43 is isolated in 82% overall yield. It is important to note the lack of competing hydrolysis of the intermediate ester after the increase of pH upon addition of base, presumably reflecting the preferred location of newly formed ester 41 within the lipophilic inner micellar cores, rather than in the basic aqueous medium.

**Scheme 5:**
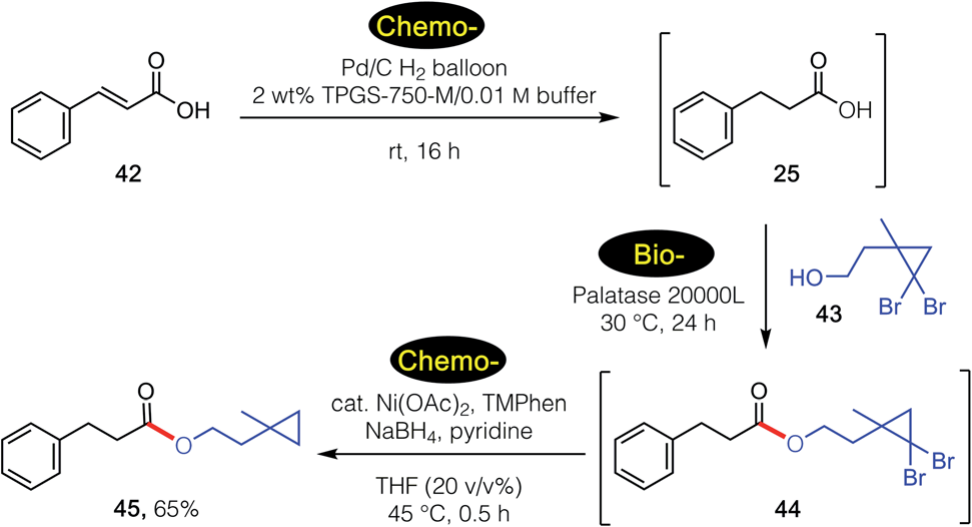
3-Step tandem 1-pot chemo-/bio-/chemo-catalysis sequence in an aqueous micellar medium.

These tandem, mixed bio- and chemo-catalysis sequences are not limited to two steps. As shown in Scheme 5, catalytic hydrogenation of cinnamic acid 44 in water in the presence of the Pd/C^27^ led to intermediate acid 20. Subsequent lipase-catalyzed esterification with alcohol 45 gave *gem*-dibromocyclopropane 46, which was then reduced by nickel nanoparticles^28^ to give product 47 in 65% overall isolated yield, all done, sequentially, in water.

**Scheme 6:**
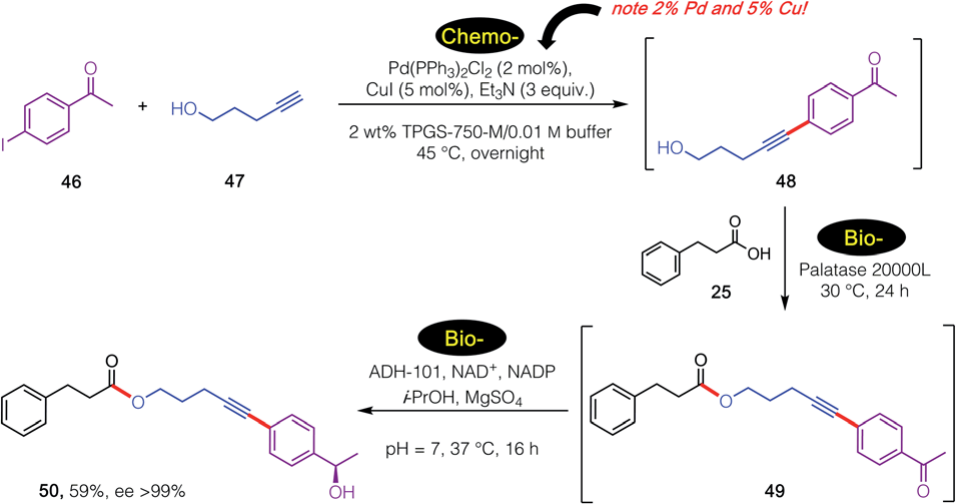
A 1-pot, 3-step chemo-/bio-/bio-sequence in an aqueous micellar medium.

Further extending the options for this approach to chemoenzymatic catalysis and, importantly, substantiating enzymatic compatibility, another 3-step sequence was carried out as illustrated in Scheme 6. In this case, an initial, traditional Pd-catalyzed Sonogashira coupling was performed^29^ specifically as a test of enzymatic compatibility with such high, indeed unsustainable amounts of both Pd and Cu.^30^ Thus, and notwithstanding these levels of metal present in the aqueous medium, initial product alcohol 50 was successfully subjected (after adjustment to pH = 2) to lipase-catalyzed esterification.^31^ The 1-pot reaction mixture containing ester 51 was subsequently adjusted to pH 7, after which addition of an alcohol dehydrogenase (ADH-101)^18^ provided secondary nonracemic alcohol 52 in 59% overall isolated yield (ee >99%).

**Scheme 7:**
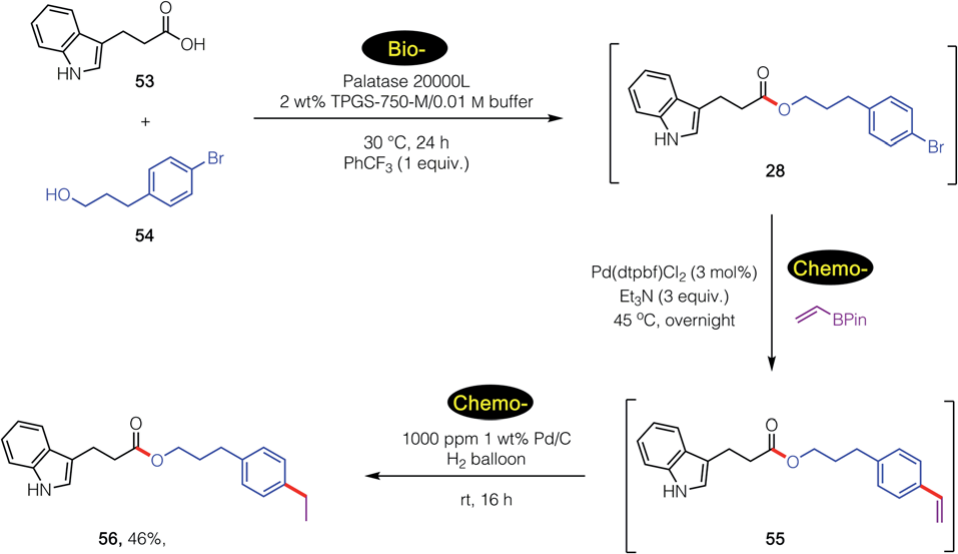
A 1-pot, 3-step bio-/chemo-/chemo-sequence in an aqueous micellar medium.

An even more complex carboxylic acid containing an indole (53) was also amenable to a tandem, 1-pot process (Scheme 7). An initial biocatalytic esterification to 28, which benefited from the presence of PhCF_3_ (*vide supra*; Fig. 2), was followed (without isolation) by two consecutive chemocatalysis steps: a Pd-catalyzed Suzuki–Miyaura vinylation to 55, and thence olefin reduction^27^ to arrive at ester 56. It is worthy of note that the first, selective enzymatic step takes place without protecting group chemistry (of the indole), in further alignment with the 12 Principles of Green Chemistry.^32,33^

## Conclusions

An economically attractive, commercially available lipase has been identified shown to effectively catalyze esterification reactions “in water”, requiring an ideal 1 : 1 ratio of acid and alcohol. The enzymatic process is not only tolerant of the nanomicelles present in the aqueous medium, derived from TPGS-750-M, but is made all the more effective in terms of reaction conversion as well as avoidance of competitive hydrolysis by their presence. Also discussed is the unusual and unexpected finding that by adding PhCF_3_ (1 equiv. relative to substrate) to the reaction medium the extent of conversion can be further advanced. Alteration of the enzymatic pocket using such an uncharacteristically simple additive may present exciting opportunities for extending enzymatic tolerance of additional structural features associated with a broader range of substrates of interest. Unprecedented 1-pot sequences described herein suggest that chemo- and bio-catalysis can now be used in variable combinations within the same aqueous medium, making this approach to synthesis especially attractive in terms of time and pot economy, and perhaps most noteworthy, environmental friendliness.

## Author contributions

All authors contributed equally to the preparation of this ms.

## Conflicts of interest

The authors declare no conflict of interest.

## Supplementary Material

SC-013-D1SC05660C-s001
