# Supplementary material for: Lipase-catalyzed esterification in water enabled by nanomicelles. Applications to 1-pot multi-step sequences
Source: Chem Sci. 2021 Dec 27;13(5):1440–5. doi: 10.1039/d1sc05660c (PMC8809412; doi:10.1039/d1sc05660c)
Supplement: SC-013-D1SC05660C-s001 [file SC-013-D1SC05660C-s001.pdf]

Electronic Supplementary Material (ESI) for Chemical Science.
This journal is © The Royal Society of Chemistry 2021

Electronic Supplementary Information

Lipase-catalyzed *esterification in water* enabled by nanomicelles. Applications to 1-pot multi-step sequences

Vani Singhania,[a] Margery Cortes-Clerget,[a] Bhornrawin Akkachairin,[a] [b] Jade Dussart-Gautheret,[a] Julie Yu,[a] Nnamdi Akporji,[a] and Bruce H. Lipshutz[a] *

[a] Department of Chemistry and Biochemistry, University of California, Santa Barbara, CA, 93106 USA

[b] Program on Chemical Biology, Chulabhorn Graduate Institute, Center of Excellence on Environmental Health and Toxicology (EHT), Ministry of Education, 54 Kamphaeng Phet 6, Laksi, Bangkok 10210 Thailand

Phone : 805-893-2521
Fax : 805-893-8265
Email: lipshutz@chem.ucsb.edu

Table of Contents

General Information 2
Preparation of the buffer solution 2
General approach to optimization of esterification reactions 3
Conversion measurement 3
Optimized procedure for esterification reactions 4
General procedure for esterification using the additive $PhCF_3$ 4
Enzyme study – Table S1 4
Reaction optimization – Table S2 5
Buffer concentration study – Figure S1 5
Surfactant concentration study – Figure S2 6
2-step, 1-pot: esterification, Suzuki-Miyaura cross-coupling 7
3-step, 1-pot sequence: Pd/C reduction, esterification, *gem*-dibromocyclopropane reduction 8
3-step, 1-pot sequence: Sonogashira cross-coupling reaction, esterification, ADH reduction 10
3-step, 1-pot sequence: esterification, Suzuki cross-coupling reaction, Pd/C reduction 11
References 12
Analytical data for esterification reaction products 12

General Information

Palatase 20000L (originating from *Rhizomucor miehei*) was purchased from Strem Chemicals Inc. (cat. no. 06-3118).

https://www.strem.com/catalog/v/06-3118/101/biocatalysts_9001-62-1

Lipozyme ® RM was purchased from Strem Chemicals Inc. (cat. no. 06-3120).

https://www.strem.com/catalog/v3/06-3120/101/biocatalysts_9001-62-1

Amano Lipase PS was purchased from Aldrich (cat. no. 534641). https://www.sigmaaldrich.com/US/en/product/aldrich/534641

Lipase form *Candida rugosa* was purchased from Sigma (cat. no. L1754).

https://www.sigmaaldrich.com/US/en/product/sigma/l1754

Lipase from *Rhizopus niveus* was purchased from Sigma (cat. no. 62310).

https://www.sigmaaldrich.com/US/en/product/sigma/62310

ADH101 is commercially available from the enzyme kit EZK-001 from Johnson Matthey. TPGS-750-M is available from Sigma-Aldrich (catalog #733857 (solution) or #763896 (wax)) or can be prepared following the reported procedure.[1] Potassium phosphate monobasic and dibasic were purchased from Sigma Aldrich. All commercially available reagents were used without further purification. Thin layer chromatography (TLC) was done using Silica Gel 60 F254 plates (Merck, 0.25 mm thick). Flash chromatography was done in glass columns using Silica Gel 60 (EMD, 40-63 μm). ^{1}H and ^{13}C NMR were recorded at 25 °C either on a Varian Unity Inova 400 MHz, a Varian Unity Inova 500 MHz or on a Varian Unity Inova 600 MHz spectrometers in $CDCl_3$ with residual $CHCl_3$ (^{1}H = 7.27 ppm, ^{13}C = 77.16 ppm) as internal standard. Chemical shifts are reported in parts per million (ppm). Data are reported as follows: chemical shift, multiplicity (s = singlet, d = doublet, t = triplet, q = quartet, quin = quintet, m = multiplet), coupling constant (if applicable) and integration. Chiral HPLC data was collected using an Agilent 1220 HPLC. HRMS data were recorded on a Waters Micromass LCT TOF ES+ Premier mass spectrometer using ESI ionization.

Preparation of the buffer solution

Aqueous 1 M stock solutions of potassium phosphate monobasic (A) and potassium phosphate dibasic (B) were prepared. A pH=7 phosphate buffer solution was then prepared by

mixing 38.5 mL of solution A with 61.5 mL of solution B. The pH was controlled and adjusted, if needed, with a 1 M solution of NaOH or HCl. The buffer solution was diluted with HPLC grade water (to 0.01 M, 0.05 M, 0.2 M or 0.4 M). 2 wt % of TPGS-750-M, as a wax, was dissolved and used as media of the reaction. 1, 4 and 6 wt % of TPGS-750-M in the buffer solution have also been prepared.

General approach to optimization of esterification reactions

The evaluation of the impact of various parameters has been set up on the esterification between valeric acid and hexanol.

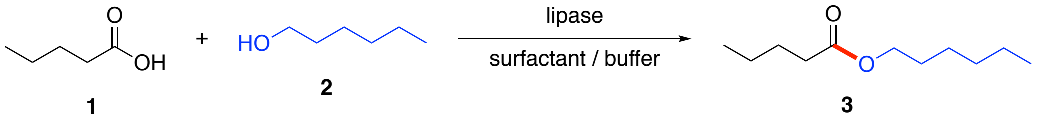

All the reactions have been set up on a 0.5 mmol scale. The source of the enzyme, acid/alcohol ratio, the concentration regarding the limiting starting material, the buffer concentration, the temperature and the TPGS-750-M amount have been evaluated.[a,b]

To a 1 dr vial was added, in succession valeric acid (54 µL, 0.5 mmol, 1 equiv), *n*-hexanol (63-314 µL 0.5-2.5 mmol, 1-5 equiv) and 1 mL of 1-6 wt % TPGS-750-M/buffer [0.01-0.4 M]. The lipase (25 µL; 600 U – enzyme activity is 20000 lipase units per gram) was added and the reaction was stirred at the defined temperature (22-50 ° C) for 24 h. After completion, the pH of the reaction was lowered to 1-2 with a 1 M HCl solution. The product and remaining starting materials were extracted with 2 x 1 mL of EtOAc. The organic layer was dried with anhydrous $MgSO_4$ and concentrated *in vacuo*.

Conversion measurement

From the general procedure, the crude material was analyzed by ^{1}H NMR. The ratio between the triplet at 2.36 ppm (acid) and the triplet at 2.31 ppm (ester) was calculated to determine the conversion of the reaction.

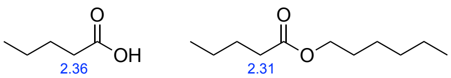

Optimized procedure for esterification reactions

To a 1 dr vial was added, in succession the carboxylic acid (0.5 mmol, 1 equiv), the alcohol (0.5 mmol, 1 equiv) and 1 mL of 2 wt % TPGS-750-M/buffer [0.01 M]. The lipase (600 U) was added and the reaction was stirred at the defined temperature (30 ° C) for 24 h. After completion, the reaction was extracted with 2 x 1 mL of EtOAc. The organic layer was dried with anhydrous $MgSO_4$, filtered through a pad of silica to remove residual surfactant and concentrated *in vacuo*.

General procedure for esterification using the additive $PhCF_3$

To a 1 dr vial containing a stir bar the carboxylic acid (0.5 mmol, 1 equiv), the alcohol (0.5 mmol, 1 equiv) and 1 mL of 2 wt % TPGS-750-M/buffer [0.01 M] was added in that order. The additive was added (0.5 mmol, 1 equiv) followed by the lipase (600 U) and the reaction was stirred at the defined temperature (30 ° C). After 24 h, the reaction was extracted with 2 x 1 mL of EtOAc. The organic layer was dried with anhydrous $MgSO_4$, filtered through a pad of silica to remove residual surfactant and concentrated *in vacuo*.

Table S1. Enzyme study

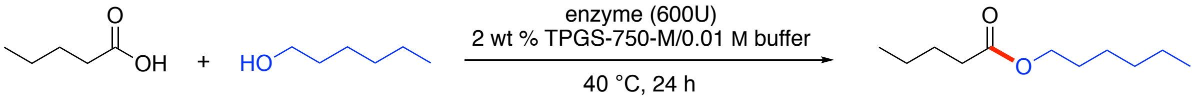

1 **2** **3**

entry	concentration [M]	ratio acid/alcohol	lipase (conversion) *candida rugosa*	*rhizopus niveus*	*rhizomucor miehei*	*burkholderia cepacia*
1	0.25	1:1	32	52	66	n.d.
2		1:3	22	60	78	54
3		1:5	21	33	**91**	80
4	0.5	1:1	17	n.d.	**83**	58
5		1:1	n.d.	n.d.	47[1]	n.d.

6		1:3	16	33	79	**85**
7		1:3	n.d.	n.d.	0[2]	n.d.
8		1:5	22	41	79	74

Conversion determined by crude ^{1}H NMR. [1] performed in the absence of surfactant, [2] pH adjusted to 7 at t = 0.

Table S2. Reaction optimization

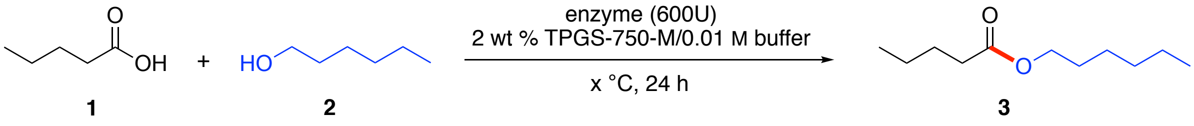

entry	Temperature (° C)	enzyme	ratio acid/alcohol	concentration [M]	conversion (%)
1	30	*rhizomucor miehei*	1:5	0.25	83
2	40	*rhizomucor miehei*	1:5	0.25	91
3	50	*rhizomucor miehei*	1:5	0.25	21
4	rt (22)	*rhizomucor miehei*	1:1	0.5	74
**5**	**30**	***rhizomucor miehei***	**1:1**	**0.5**	**99**
6	40	*rhizomucor miehei*	1:1	0.5	83
7	50	*rhizomucor miehei*	1:1	0.5	9
8	30	*burkholderia cepacia*	1:3	0.5	72
9	40	*burkholderia cepacia*	1:3	0.5	91
10	50	*burkholderia cepacia*	1:3	0.5	81

Buffer concentration study – Figure S1

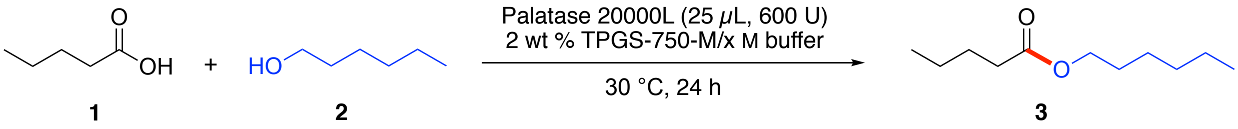

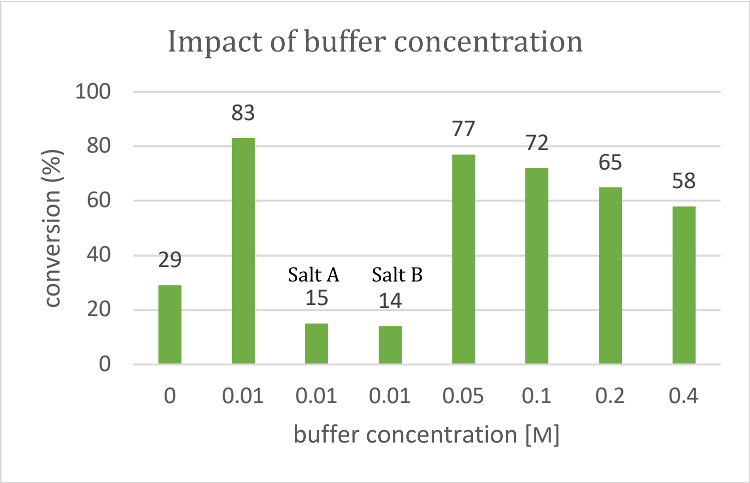

Salt A: Na_2SO_4; Salt B: NaSCN

To evaluate the impact of the buffer concentration on the conversion of the ester, **3,** various concentrations of buffer were studied. To a 1 dr vial was added, in succession the carboxylic acid (0.5 mmol, 1 equiv), the alcohol (0.5 mmol, 1 equiv) and 1 mL of 2 wt % TPGS-750-M/buffer [0-0.4 M]. The lipase (600 U) was added and the reaction was stirred at the defined temperature (30 °C) for 24 h. After completion, the reaction was extracted with 2 x 1 mL of EtOAc. The organic layer was dried with anhydrous $MgSO_4$, filtered through a pad of silica to remove residual surfactant and concentrated *in vacuo*. The samples were analyzed by ^{1}H NMR to determine the conversion.

Figure S2. Surfactant concentration study

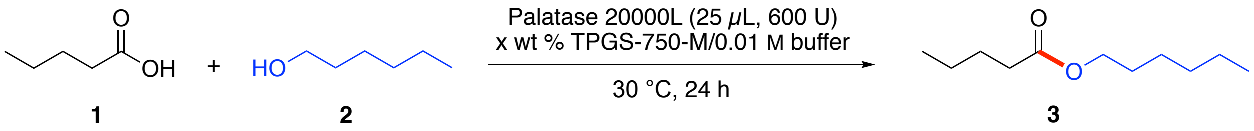

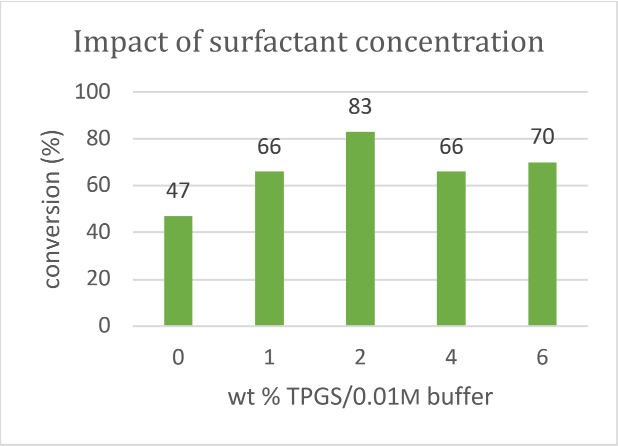

To evaluate the impact of the surfactant concentration on the conversion of reaction between **1** and **2,** various concentrations were studied. To a 1 dr vial was added, in succession the carboxylic acid (0.5 mmol, 1 equiv), the alcohol (0.5 mmol, 1 equiv) and 1 mL of 0-6 wt % TPGS-750-M/buffer [0.01 M]. The lipase (600 U) was added and the reaction was stirred at the defined temperature (30 ° C) for 24 h. After completion, the reaction was extracted with 2 x 1 mL of EtOAc. The organic layer was dried with anhydrous $MgSO_4$, filtered through a pad of silica to remove residual surfactant and concentrated *in vacuo*. The samples were analyzed by ^{1}H NMR to determine the conversion.

2-step, 1-pot: esterification, Suzuki-Miyaura cross-coupling

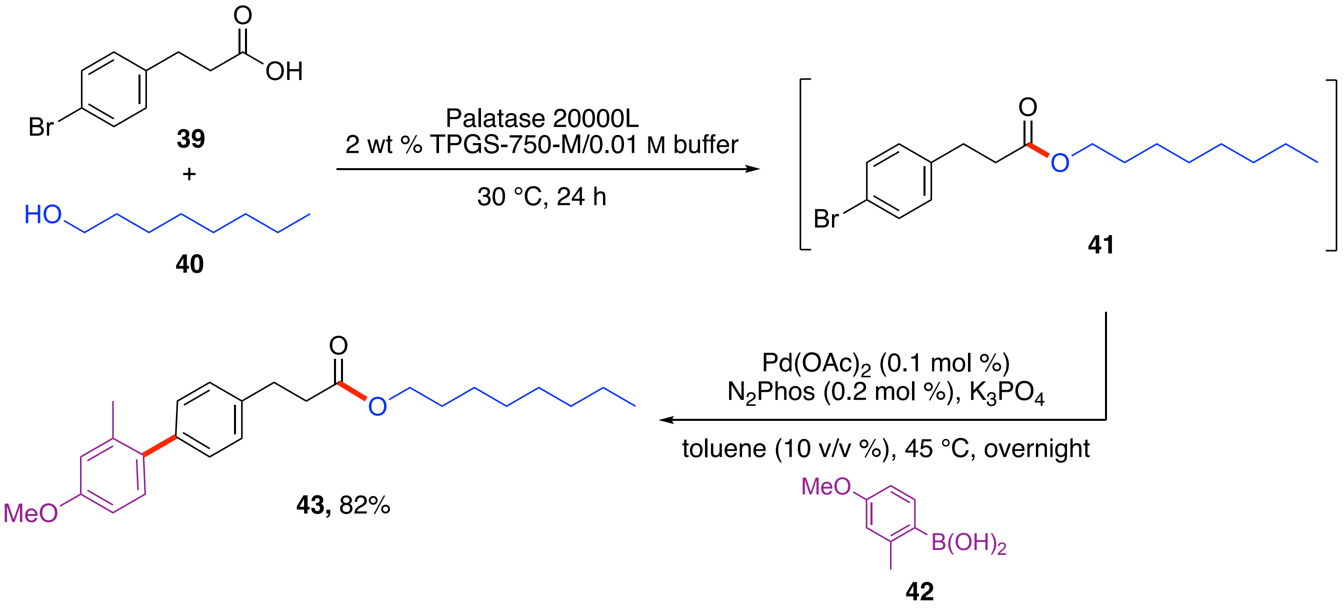

To a 1 dr vial was added 3-(4-bromophenyl)propionic acid (116.9 mg, 0.5 mmol, 1 equiv), octanol (78 µL, 0.5 mmol, 1 equiv) and 2 wt % TPGS-750-M/buffer (1 mL [0.5 M]/0.01 M phosphate buffer at pH = 7). Palatase 20000L (25 µL) was added and the reaction was stirred at 30 °C for 24 h. After completion, 2-methyl-4-methoxyphenyl boronic acid (124.5 mg, 0.75 mmol, 1.5 equiv) was added, followed by K_3PO_4 (159.2 mg, 0.75 mmol, 1.5 equiv). Finally, the catalyst solution* (0.1 mL, 1000 ppm) was added and the reaction was stirred at 45 °C overnight. Upon completion of the reaction, the vial was cooled to rt. The mixture was extracted with ethyl acetate (3 x 3 mL), and the organic layer was washed with brine three times. The layers were then separated and the organic layer was dried over anhydrous $MgSO_4$. The mixture was concentrated *in vacuo*.

* Catalyst stock solution contains $Pd(OAc)_2$ (1.1 mg) and N_2Phos (7.6 mg) in toluene (1 mL).[c]

3-step, 1-pot sequence: Pd/C reduction, esterification, *gem*-dibromocyclopropane reduction

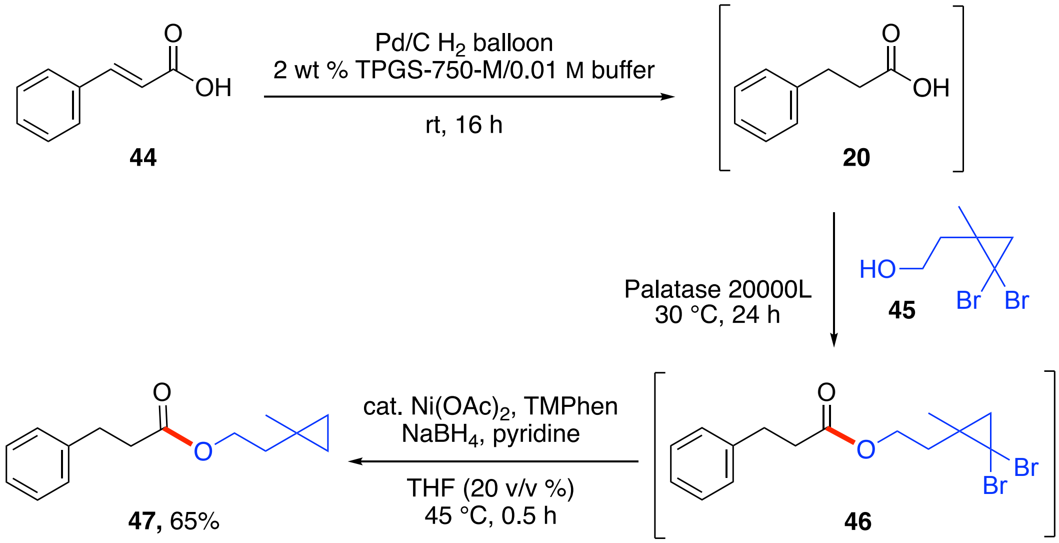

In a 1-dram vial containing equipped with a stir bar, 1 wt % Pd/C (2.6 mg), alkene (74.1 mg, 0.5 mmol, 1 equiv) and 2 wt % TPGS-750-M/buffer (1 mL [0.5 M]/0.01 M phosphate buffer at pH = 7) were added.[d] The reaction vial was sealed with a septum which was punctured with a needle (18 G) attached with pre-filled balloon of hydrogen gas. The vial was purged with the H_2 and a vent needle for ca. 2 min. The vent needle was removed and another H_2 filled balloon was then attached to the reaction vial. The reaction mixture was stirred at rt or 45 °C. Upon completion, Palatase

20000L (25 μL) was added and the reaction was set to stir for 24 h. Upon completion the intermediate was extracted with MTBE and concentrated *in vacuo*. In a 25 mL round-bottom flask equipped with a stir bar was added $Ni(OAc)_2$ (9 mg) under argon. The reaction vial was cooled to 0 ^{o}C and 2 wt % TPGS-750-M/H_2O (1.5 mL) was added to the reaction vial and the mixture was set to stir for 10 min. Pyridine (0.1 mL) was added to the reaction vial and the mixture was set to stir for an additional 5 min. $NaBH_4$ (189.15 mg, 2.5 mmol, 5 equiv) was then added in one portion. The crude reaction material from the previous step was dissolved in THF (300 μL) and added as a solution to the flask through the septum. The vial containing the crude mixture was rinsed with another portion of THF (300 μL, 20 v/v % total). A 12 mL syringe (containing 0.1 mL of THF) was added through the top of the septum to accept evolving hydrogen gas. The reaction was stirred for 30 min at 45 °C. If the volume of gas generated exceeds the volume of the syringe, the syringe must be emptied outside of the flask and adapted again.[e] After completion, the reaction was dissolved in EtOAc (10 mL) and filtered through a pad of silica. The pad was rinsed with EtOAc (3 x 10 mL). The organic layer was dried over anhydrous $MgSO_4$, filtered, and concentrated under vacuum. Purification by flash chromatography using hexanes and EtOAc (85:15) was performed to afford the product.

3-step, 1-pot sequence: Sonogashira cross-coupling reaction, esterification, ADH reduction

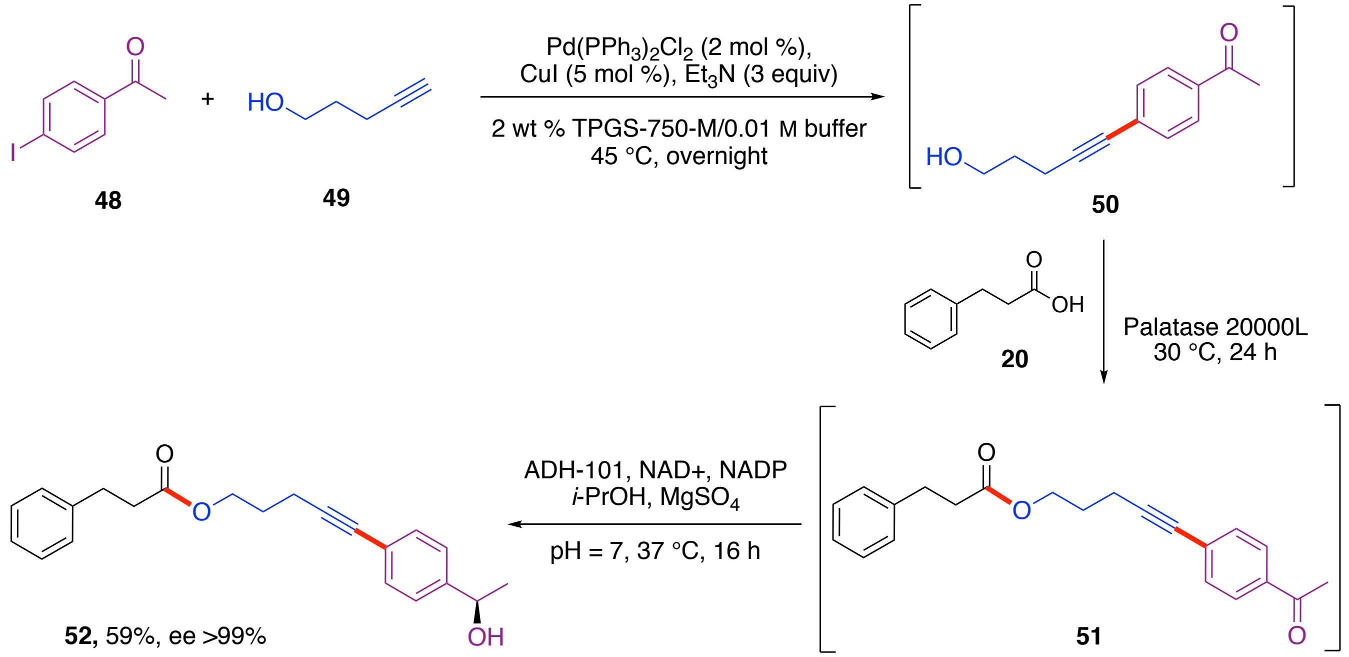

To a dried 1-dram vial were added, under an argon atmosphere, $Pd(PPh_3)_3Cl_2$ (2.8 mg, 2 mol %), CuI (1.9 mg, 5 mol %), aryl iodide (49.2 mg, 1 equiv, 0.2 mmol), alkyne (27.9 µL, 1 equiv, 0.2 mmol) and Et_3N (84.0 µL, 3 equiv). The vial was capped with a rubber septum. A 2 wt % TPGS-750-M/buffer (0.4 mL [0.5 M]/0.01 M phosphate buffer at pH = 7) was added. The reaction was stirred at 45 °C under argon until completion. The pH was adjusted to 3 with a solution of HCl (1 M) and the mixture became opaque white. Then, 3-phenylpropionic acid (30.0 mg, 1 equiv, 0.2 mmol) and Palatase 20000L (10 µL) were added and the reaction was set to stir at 30 °C for 24 h. The concentration was adjusted to [0.056 M] by adding 2.6 mL of a 2 wt % TPGS-750-M/buffer solution (phosphate, [0.23 M], pH = 7). $MgSO_4$ (0.8 mg), NAD+ (2.6 mg), NADP+ (2.4 mg), *i*-PrOH (0.6 mL) and ADH101 (20.0 mg) was added in succession.[b] The reaction was stirred at 37 °C until completion. The reaction was extracted in EtOAc. The organic layer was washed with H_2O, dried over anhydrous $MgSO_4$, filtered and concentrated under vacuum. Purification by flash chromatography using hexanes and EtOAc (85:15) was performed to afford (59%, 40 mg) of the product.

3-step, 1-pot sequence: esterification, Suzuki cross-coupling reaction, Pd/C reduction

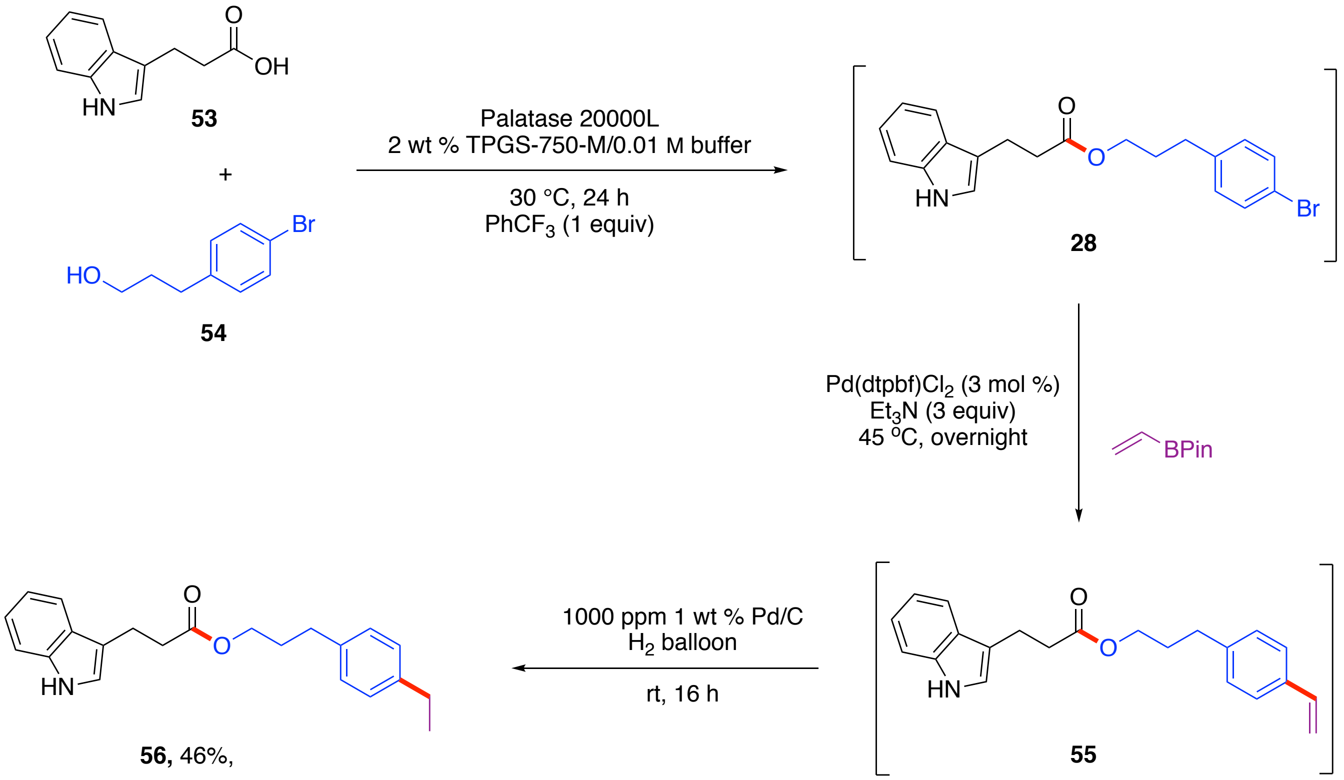

To a 1-dram vial equipped with a stirrer, 3-(4-bromophenyl)propan-1-ol (0.25 mmol, 53.8 mg, 1 equiv), 3-(1*H*-indol-3-yl), propanoic acid (0.25 mmol, 47.3 mg, 1 equiv) and trifluoromethyl-benezene (0.25 mmol, 36.5 mg, 1 equiv) were added. Followed by 2 wt % TPGS-750-M/buffer (0.5 mL [0.5 M]/0.01 M phosphate buffer at pH = 7). The resulting mixture was allowed to stir to emulsify. Finally, the enzyme was added. The reaction mixture was stirred vigorously at 30 °C for 24 h. To the reaction vial are then added Pd(dtbpf)Cl_2 (0.06 mmol, 4.9 mg, 3 mol %), triethylamine (0.75 mmol, 75.9 mg, 3 equiv) and vinylboronic acid pinacol ester (0.26 mmol, 40.4 mg, 1.05 equiv). The reaction was stirred at 45 °C. Upon completion (monitored by TLC), the reaction was charged with Pd/C (1 wt % Pd loading; 2.6 mg). The reaction was stirred at rt under a balloon of hydrogen.[d] After the reaction completed (monitored by proton NMR), the reaction was subjected to flash column chromatography to obtain purified product.

References

(a) Lipshutz, B. H.; Ghorai, S.; Abela, A. R.; Moser, R.; Nishikata, T.; Duplais, C.; Krasovskiy, A.; Gaston, R. D.; Gadwood, R. C. TPGS-750-M: A Second-Generation Amphiphile for Metal-Catalyzed Cross-Couplings in Water at Room Temperature. *J. Org. Chem.* **2011**, *76*, 4379–4391.

(b) Cortes-Clerget, M.; Akporji, N.; Zhou, J.; Gao, F.; Guo, P.; Parmentier, M.; Gallou, F.; Berthon, J.-Y.; Lipshutz, B. H. Bridging the Gap between Transition Metal- and Bio-Catalysis via Aqueous Micellar Catalysis. *Nat. Comm.* **2019**, *10*, 2169.

(c) Akporji, N.; Thakore, R. R.; Cortes-Clerget, M.; Andersen, J.; Landstrom, E.; Aue, D. H.; Gallou, F.; Lipshutz, B. H. N_2Phos – an Easily Made, Highly Effective Ligand Designed for Ppm Level Pd-Catalyzed Suzuki–Miyaura Cross Couplings in Water. *Chem. Sci.* **2020**, *11*, 5205–5212.

(d) Takale, B. S.; Thakore, R. R.; Gao, E. S.; Gallou, F.; Lipshutz, B. H. Environmentally responsible, safe, and chemoselective catalytic hydrogenation of olefins: ppm level Pd catalysis in recyclable water at room temperature. *Green Chem.* **2020**, *22*, 6055-6061.

(e) Wood, A. B.; Cortes-Clerget, M.; Kincaid, J. R. A.; Akkachairin, B.; Singhania, V.; Gallou, F.; and Lipshutz, B.H. Nickel Nanoparticle Catalyzed Mono- and Di-Reductions of Gem-Dibromocyclopropanes Under Mild, Aqueous Micellar Conditions. *Angew. Chem., Int. Ed.* **2020**, *59*, 17587-17593.

Analytical data for reaction products from esterification

n-Hexyl pentanoate (**3**)

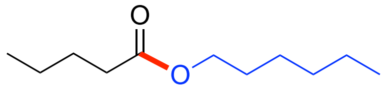

^{1}H NMR (500 MHz, $CDCl_3$) δ 4.06 (t, *J* = 6.7 Hz, 2H), 2.30 (t, *J* = 7.6 Hz, 2H), 1.62 (dtt, *J* = 11.5, 7.7, 3.6 Hz, 4H), 1.41 – 1.23 (m, 8H), 0.98 – 0.85 (m, 6H); **^{13}C NMR (126 MHz, $CDCl_3$) δ** 174.1, 64.5, 34.3, 31.6, 28.8, 27.2, 25.7, 22.7, 22.4, 14.1, 13.8. **Yield**: 84%, 78.2 mg; pale yellow oil. **R_f** = 0.73 (95:5 hexanes/EtOAc). Spectral data matched those previously reported.[1]

n-Octyl undec-10-enoate (**4**)

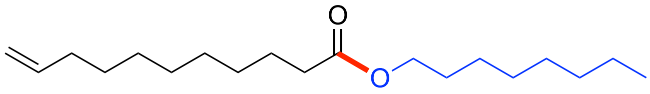

[1] Seitz, L. M.; Ram, M. S. Metabolites of Lesser Grain Borer in Grains. *J. Agric. Food Chem.* **2004**, *52*, 898–908.

^{1}H NMR (600 MHz, CDCl$_3$) δ 5.82 (ddt, *J* = 16.9, 10.2, 6.7 Hz, 1H), 5.00 (dq, *J* = 17.1, 1.7 Hz, 1H), 4.94 (ddt, *J* = 10.2, 2.3, 1.2 Hz, 1H), 4.06 (t, *J* = 6.7 Hz, 2H), 2.30 (t, *J* = 7.5 Hz, 2H), 2.08 – 2.01 (m, 2H), 1.67 – 1.59 (m, 4H), 1.43 – 1.22 (m, 20H), 0.89 (t, *J* = 7.0 Hz, 3H); **^{13}C NMR (151 MHz, CDCl$_3$) δ** 174.2, 139.3, 114.3, 64.6, 34.6, 33.9, 31.9, 29.4, 29.4, 29.3, 29.3, 29.2, 29.0, 28.8, 26.1, 25.2, 22.8, 14.2. **Yield**: 75%, 111.0 mg; colorless oil. **R$_f$** = 0.53 (90:10 hexanes/EtOAc). Spectral data matched those previously reported.[2]

(*E*)-3,7-Dimethylocta-2,6-dien-1-yl dodecanoate (**5**)

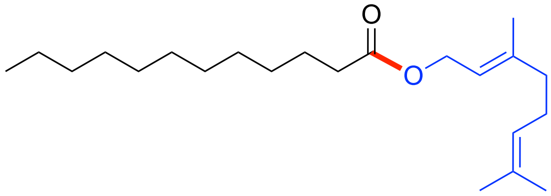

^{1}H NMR (500 MHz, CDCl$_3$) δ 5.34 (tt, *J* = 7.0, 1.4 Hz, 1H), 5.09 (tt, *J* = 6.9, 1.5 Hz, 1H), 4.60 (d, *J* = 7.1 Hz, 2H), 2.30 (t, *J* = 7.6 Hz, 2H), 2.15 – 2.08 (m, 2H), 2.05 (dd, *J* = 8.7, 6.5 Hz, 2H), 1.70 (dd, *J* = 9.6, 1.4 Hz, 6H), 1.67 – 1.56 (m, 5H), 1.28 (d, *J* = 15.3 Hz, 17H), 0.89 (t, *J* = 6.9 Hz, 3H); **^{13}C NMR (126 MHz, CDCl$_3$) δ** 174.076, 142.237, 131.951, 123.912, 118.582, 61.313, 39.680, 34.553, 32.057, 29.750, 29.614, 29.480, 29.420, 29.305, 26.454, 25.817, 25.171, 22.830, 17.824, 16.606, 14.253. **Yield:** 77%, 133.1 mg; colorless oil. **R$_f$** = 0.61 (95:5 hexanes/EtOAc). **Chemical Formula**: HRMS (ESI) *m/z*: [M+Na]$^+$ calcd. for $C_{22}H_{40}O_2Na$ 359.2926; found 359.2928.

n-Hexyl 3-phenylpropanoate (**6**)

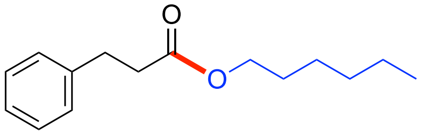

^{1}H NMR (600 MHz, CDCl$_3$) δ 7.31 – 7.26 (m, 2H), 7.23 – 7.18 (m, 3H), 4.06 (t, *J* = 6.7 Hz, 2H), 2.95 (t, *J* = 7.8 Hz, 2H), 2.65 – 2.61 (m, 2H), 1.62 – 1.56 (m, 2H), 1.35 – 1.22 (m, 6H), 0.89 (t, *J* = 6.9 Hz, 3H); **^{13}C NMR (126 MHz, CDCl$_3$) δ** 173.0, 140.6, 128.5, 128.3, 126.2, 64.6, 35.9, 31.4, 31.0, 28.6, 25.5, 22.5, 14.0. **Yield:** 72%, 84.2 mg; colorless oil. **R$_f$** = 0.46 (90:10 hexanes/EtOAc). **Chemical Formula:** HRMS (CI) *m/z*: [M+H]$^+$ calcd. for $C_{15}H_{23}O_2$ 235.1698; found 235.1694.

---

[2] Stenhagen, E.; Abrahamsson, S.; McLaffcrty, F. W. (eds.) *Archives of Mass Spectral Data*. Interscience Publishers, New York 1970.

Phenethyl 3-phenylpropanoate (**7**)

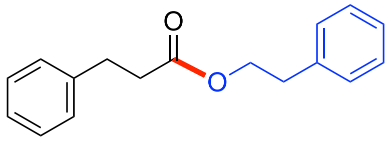

^{1}H NMR (600 MHz, $CDCl_3$) δ 7.31 – 7.25 (m, 4H), 7.25 – 7.15 (m, 6H), 4.29 (t, *J* = 7.1 Hz, 2H), 2.94 – 2.88 (m, 4H), 2.61 (t, *J* = 7.8 Hz, 2H); **^{13}C NMR (126 MHz, $CDCl_3$) δ** 172.7, 140.4, 137.7, 128.8, 128.4, 128.2, 126.5, 126.2, 64.9, 35.8, 35.0, 30.8. **Yield:** 76%, 95.0 mg; colorless oil. **R_f** = 0.50 (90:10 hexanes/EtOAc). **Chemical Formula:** HRMS (ESI) *m/z*: $[M+Na]^+$ calcd. for $C_{17}H_{18}O_2Na$ 277.1205; found 277.1204.

4-Methoxybenzyl 3-phenylpropanoate (**8**)

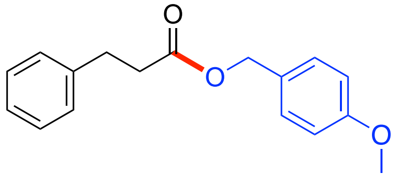

^{1}H NMR (500 MHz, $CDCl_3$) δ 7.31 – 7.25 (m, 4H), 7.24 – 7.17 (m, 3H), 6.92 – 6.87 (m, 2H), 5.06 (s, 2H), 3.83 (s, 3H), 2.97 (t, *J* = 7.8 Hz, 2H), 2.67 (dd, *J* = 8.4, 7.2 Hz, 2H); **^{13}C NMR (126 MHz, $CDCl_3$) δ** 172.7, 159.6, 140.4, 130.0, 128.4, 128.3, 128.0, 126.2, 113.9, 66.1, 55.2, 35.9, 30.9. **Yield:** 70%, 95.2 mg; pale yellow oil. **R_f** = 0.49 (80:20 hexanes/EtOAc). **Chemical Formula:** HRMS (CI) *m/z*: $[M]^+$ calcd. for $C_{17}H_{18}O_3$ 270.1256; found 270.1246.

4-Chlorobenzyl 3-phenylpropanoate (**9**)

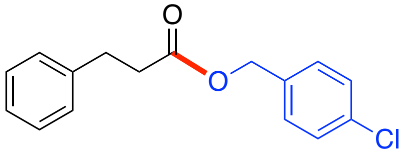

^{1}H NMR (500 MHz, $CDCl_3$) δ 7.33 – 7.25 (m, 4H), 7.24 – 7.16 (m, 5H), 5.07 (s, 2H), 2.97 (t, *J* = 7.8 Hz, 2H), 2.69 (dd, *J* = 8.2, 7.2 Hz, 2H); **^{13}C NMR (126 MHz, $CDCl_3$) δ** 172.5, 140.2, 134.4, 134.0, 129.5, 128.7, 128.5, 128.2, 126.3, 65.4, 35.8, 30.9. **Yield:** 60%, 82.9 mg; pale yellow oil. **R_f** = 0.53 (80:20 hexanes/EtOAc). **Chemical Formula:** HRMS (ESI) *m/z*: $[M+Na]^+$ calcd. for $C_{16}H_{15}ClO_2Na$ 297.0658; found 297.0663.

6-(4-Acetylphenyl)hex-5-yn-1-yl 3-phenylpropanoate (**10**)

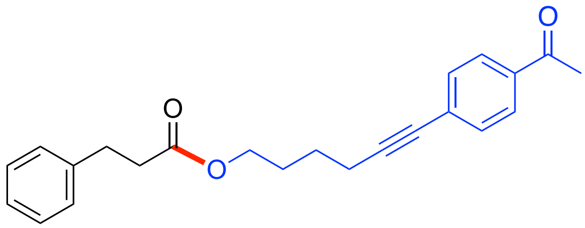

^{1}H NMR (600 MHz, $CDCl_3$) δ 7.90 – 7.86 (m, 2H), 7.48 – 7.44 (m, 2H), 7.31 – 7.27 (m, 2H), 7.22 – 7.18 (m, 3H), 4.13 (t, *J* = 6.5 Hz, 2H), 2.96 (t, *J* = 7.8 Hz, 2H), 2.64 (dd, *J* = 8.3, 7.3 Hz, 2H), 2.59 (s, 3H), 2.45 (t, *J* = 7.0 Hz, 2H), 1.82 – 1.75 (m, 2H), 1.67 – 1.61 (m, 2H); **^{13}C NMR (126 MHz, $CDCl_3$) δ** 197.3, 172.9, 140.4, 135.7, 131.6, 128.8, 128.4, 128.2, 128.1, 126.2, 93.3, 80.5, 63.9, 35.8, 30.9, 27.8, 26.5, 24.9, 19.1. **Yield:** 74%, 129.3 mg; colorless oil. **R$_f$** = 0.50 (70:30 hexanes/EtOAc). **Chemical Formula:** HRMS (CI) *m/z*: $[M+C_2H_5]^+$ calcd. for $C_{25}H_{29}O_3$ 377.2117; found 377.2112.

(*E*)-5-(Benzo[d][1,3]dioxol-5-yl)-4-methylpent-2-en-1-yl 3-phenylpropanoate (**11**)

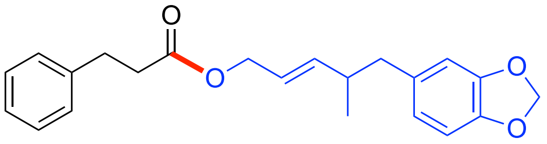

^{1}H NMR (500 MHz, $CDCl_3$) δ 7.31 – 7.27 (m, 2H), 7.23 – 7.18 (m, 3H), 6.71 (d, *J* = 7.9 Hz, 1H), 6.62 (d, *J* = 1.7 Hz, 1H), 6.57 (dd, *J* = 7.9, 1.7 Hz, 1H), 5.90 (s, 2H), 5.69 (ddt, *J* = 15.4, 6.8, 1.2 Hz, 1H), 5.46 (dtd, *J* = 15.5, 6.5, 1.2 Hz, 1H), 4.50 (d, *J* = 6.4 Hz, 2H), 2.95 (t, *J* = 7.8 Hz, 2H), 2.64 (dd, *J* = 8.5, 7.2 Hz, 2H), 2.58 (dd, *J* = 13.0, 6.4 Hz, 1H), 2.47 – 2.35 (m, 2H), 0.98 (d, *J* = 6.5 Hz, 3H); **^{13}C NMR (126 MHz, $CDCl_3$) δ** 172.6, 147.3, 145.6, 140.9, 140.5, 134.1, 128.4, 128.3, 126.2, 122.4, 122.0, 109.5, 107.9, 100.7, 65.1, 42.8, 38.2, 35.9, 30.9, 19.2. **Yield:** 81%, 142.1 mg; colorless oil. **R$_f$** = 0.54 (70:30 hexanes/EtOAc). **Chemical Formula**: HRMS (ESI) *m/z*: $[M+Na]^+$ calcd. for $C_{22}H_{24}O_4Na$ 375.1572; found 375.1576.

3-Phenylpropyl 3-(4-bromophenyl)propanoate (**12**)

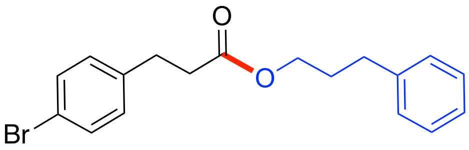

^{1}H NMR (400 MHz, CDCl$_3$) δ 7.46 – 7.40 (m, 2H), 7.34 – 7.28 (m, 2H), 7.25 – 7.15 (m, 3H), 7.15 – 7.07 (m, 2H), 4.11 (t, *J* = 6.5 Hz, 2H), 2.93 (t, *J* = 7.6 Hz, 2H), 2.65 (dt, *J* = 15.0, 7.5 Hz, 4H), 2.00 – 1.90 (m, 2H); **^{13}C NMR (101 MHz, CDCl$_3$) δ** 172.63, 141.16, 139.54, 131.61, 130.17, 128.51, 128.44, 126.09, 120.14, 77.48, 77.16, 76.84, 63.97, 35.62, 32.20, 30.39, 30.20. **Yield:** 67%, 116.6 mg; colorless oil. **R$_f$** = 0.45 (90:10 hexanes/EtOAc). **Chemical Formula:** HRMS (ESI) *m/z*: [M+Na]$^+$ calcd. for $C_{18}H_{19}BrO_2Na$ 369.0466; found 369.0465.

2-(2,2-Dibromo-1-methylcyclopropyl)ethyl 3-(4-bromophenyl)propanoate (**13**)

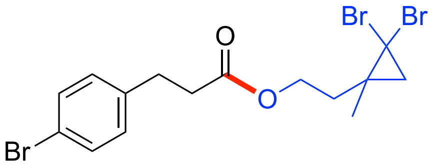

^{1}H NMR (500 MHz, CDCl$_3$) δ 7.44 – 7.38 (m, 2H), 7.12 – 7.06 (m, 2H), 4.28 (td, *J* = 7.0, 1.7 Hz, 2H), 2.93 (t, *J* = 7.7 Hz, 2H), 2.64 (t, *J* = 7.7 Hz, 2H), 1.99 (dp, *J* = 14.3, 7.1 Hz, 2H), 1.48 (d, *J* = 7.5 Hz, 1H), 1.42 (d, *J* = 7.5 Hz, 1H), 1.38 (s, 3H); **^{13}C NMR (151 MHz, CDCl$_3$) δ** 172.6, 139.5, 131.7, 130.2, 120.3, 62.0, 38.2, 37.0, 35.8, 34.5, 30.4, 27.6, 22.7. **Yield:** 69%, 161.7 mg; pale yellow oil. **R$_f$** = 0.33 (95:5 hexanes/EtOAc). **Chemical Formula:** HRMS (ESI) *m/z*: [M+Na]$^+$ calcd. for $C_{15}H_{17}Br_3O_2Na$ 490.8656; found 490.8651.

Hex-5-en-1-yl 4-oxo-4-phenylbutanoate (**14**)

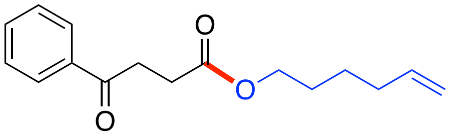

^{1}H NMR (400 MHz, CDCl$_3$) δ 7.98 (d, *J* = 7.0 Hz, 2H), 7.57 (t, *J* = 7.4 Hz, 1H), 7.46 (t, *J* = 7.6 Hz, 2H), 5.78 (ddt, *J* = 17.0, 10.3, 6.7 Hz, 1H), 5.04 – 4.92 (m, 2H), 4.10 (t, *J* = 6.6 Hz, 2H), 3.31 (t, *J* = 6.6 Hz, 2H), 2.76 (t, *J* = 6.6 Hz, 2H), 2.07 (q, *J* = 7.0 Hz, 2H), 1.70 – 1.60 (m, 2H), 1.45 (q, *J* = 7.7 Hz, 2H); **^{13}C NMR (101 MHz, CDCl$_3$) δ** 198.23, 173.08, 138.48, 136.69, 133.33, 128.73, 128.15, 114.92, 77.48, 77.16, 76.84, 64.75, 33.50, 33.39, 28.39, 28.14, 25.28. **Yield:** 38%, 48.9 mg; white solid. **R$_f$** = 0.45 (80:20 hexanes/EtOAc). **Chemical Formula:** HRMS (ESI) *m/z*: [M+Na]$^+$ calcd. for $C_{15}H_{24}O_3Na$ 283.1310; found 283.1311.

Hex-5-en-1-yl 3-(1-methyl-1H-indol-3-yl)propanoate (**15**)

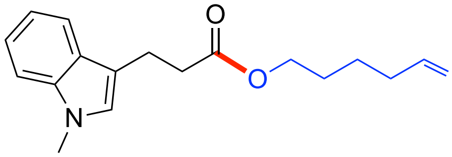

^{1}H NMR (500 MHz, $CDCl_3$) δ 7.60 (dt, *J* = 8.0, 1.0 Hz, 1H), 7.29 (dt, *J* = 8.3, 0.9 Hz, 1H), 7.23 (ddd, *J* = 8.2, 6.9, 1.2 Hz, 1H), 7.11 (ddd, *J* = 8.0, 6.9, 1.1 Hz, 1H), 6.87 (s, 1H), 5.78 (ddt, *J* = 16.9, 10.2, 6.6 Hz, 1H), 5.01 (dq, *J* = 17.1, 1.7 Hz, 1H), 4.96 (ddt, *J* = 10.2, 2.2, 1.2 Hz, 1H), 4.08 (t, *J* = 6.7 Hz, 2H), 3.74 (s, 3H), 3.10 (t, *J* = 7.5 Hz, 2H), 2.71 (dd, *J* = 8.2, 7.1 Hz, 2H), 2.10 – 2.03 (m, 2H), 1.67 – 1.58 (m, 2H), 1.45 – 1.37 (m, 2H); **^{13}C NMR (126 MHz, $CDCl_3$) δ** 173.4, 138.3, 136.9, 127.5, 126.2, 121.5, 118.7, 118.8, 114.7, 113.4, 109.1, 64.2, 35.1, 33.2, 32.5, 28.0, 25.1, 20.5. **Yield:** 48%, 68.6 mg; pale colorless oil. **R_f** = 0.45 (80:20 hexanes/EtOAc). **Chemical Formula:** HRMS (CI) *m/z*: $[M+H]^+$ calcd. for $C_{18}H_{25}NO_2$ 286.1807; found 286.1815.

n-Octyl 3-(furan-2-yl)propanoate (**16**)

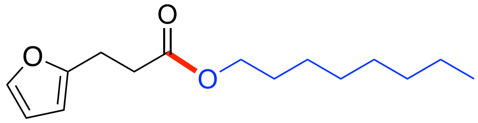

^{1}H NMR (400 MHz, $CDCl_3$) δ 7.30 (d, *J* = 1.8 Hz, 1H), 6.30 – 6.25 (m, 1H), 6.01 (d, *J* = 3.2 Hz, 1H), 4.08 (t, *J* = 6.7 Hz, 2H), 2.96 (t, *J* = 7.6 Hz, 2H), 2.65 (t, *J* = 7.6 Hz, 2H), 1.61 (t, *J* = 7.0 Hz, 2H), 1.28 (dq, *J* = 11.7, 4.6, 3.8 Hz, 10H), 0.90 – 0.86 (m, 3H); **^{13}C NMR (101 MHz, $CDCl_3$) δ** 172.75, 154.35, 141.31, 110.30, 105.36, 88.05, 77.48, 77.16, 76.84, 64.90, 32.87, 31.91, 29.33, 29.31, 28.73, 26.02, 23.63, 22.78, 14.22. **Yield:** 40%, 50.4 mg; yellowish oil. **R_f** = 0.64 (90:10 hexanes/EtOAc). **Chemical Formula:** HRMS (ESI) *m/z*: $[M+Na]^+$ calcd. for $C_{15}H_{24}O_3Na$ 275.1623; found 275.1625.

Phenethyl 3-(thiophen-2-yl)propanoate (**19**)

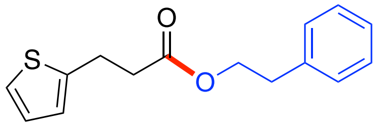

^{1}H NMR (600 MHz, $CDCl_3$) δ 7.34 (t, *J* = 7.5 Hz, 2H), 7.30 – 7.22 (m, 3H), 7.15 (dd, *J* = 5.2, 1.2 Hz, 1H), 6.94 (dd, *J* = 5.1, 3.4 Hz, 1H), 6.82 (dd, *J* = 3.5, 1.3 Hz, 1H), 4.35 (t, *J* = 7.1 Hz, 2H), 3.20 – 3.14 (m, 2H), 2.96 (t, *J* = 7.1 Hz, 2H), 2.70 (t, *J* = 7.6 Hz, 2H); **^{13}C NMR (101 MHz, $CDCl_3$) δ** 172.35, 143.10,

137.83, 128.97, 128.58, 126.91, 126.65, 124.72, 123.59, 65.13, 36.21, 35.15, 25.19. **Yield:** 72%, 93.9 mg; yellow-brown liquid. **R$_f$** = 0.3 (95:5 hexanes/EtOAc). **Chemical Formula:** HRMS (ESI) *m/z*: $[M+Na]^+$ calcd. for $C_{15}H_{16}O_2SNa$ 283.0769; found 283.0774.

Thiophen-2-ylmethyl 3-phenylpropanoate (**22**)

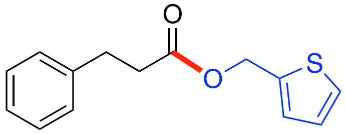

^{1}H NMR (400 MHz, CDCl$_3$) δ 7.36 – 7.17 (m, 6H), 7.09 (d, *J* = 3.6 Hz, 1H), 7.00 (dd, *J* = 5.1, 3.5 Hz, 1H), 5.29 (s, 2H), 2.98 (t, *J* = 7.8 Hz, 2H), 2.68 (t, *J* = 7.8 Hz, 2H); **^{13}C NMR (101 MHz, CDCl$_3$) δ** 172.64, 140.43, 137.98, 128.60, 128.39, 128.32, 126.95, 126.91, 126.37, 60.58, 35.91, 30.94. **Yield:** 50%, 60.8 mg; yellowish oil. **R$_f$** = 0.33 (80:20 hexanes/EtOAc). **Chemical Formula:** HRMS (ESI) *m/z*: $[M+Na]^+$ calcd. for $C_{14}H_{14}SO_2$ 269.0700; found 269.0392.

3-(6-Methylpyridin-2-yl)propyl dodecanoate (**23**)

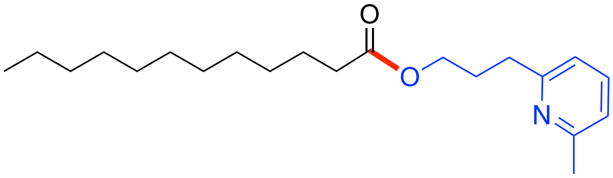

^{1}H NMR (400 MHz, CDCl$_3$) δ 7.46 (t, *J* = 7.7 Hz, 1H), 6.94 (dd, *J* = 9.9, 7.7 Hz, 2H), 4.10 (t, *J* = 6.5 Hz, 2H), 2.80 (dd, *J* = 8.8, 6.7 Hz, 2H), 2.50 (s, 3H), 2.27 (t, *J* = 7.5 Hz, 2H), 2.10 – 1.99 (m, 2H), 1.65 – 1.54 (m, 2H), 1.25 (d, *J* = 10.2 Hz, 16H), 0.86 (t, *J* = 6.6 Hz, 3H); **^{13}C NMR (101 MHz, CDCl$_3$) δ** 174.04, 160.43, 158.03, 136.69, 120.79, 119.69, 63.84, 34.86, 34.45, 32.01, 29.71, 29.57, 29.44, 29.38, 29.28, 28.91, 25.10, 24.62, 22.78, 14.22. **Yield:** 36%, 60.3 mg; colorless solid. **R$_f$** = 0.32 (80:20 hexanes/EtOAc). **Chemical Formula:** HRMS (ESI) *m/z*: $[M+H]^+$ calcd. for $C_{21}H_{36}NO_2$ 334.2746; found 334.2748.

Phenethyl 3-(4-cyanophenyl)propanoate (**24**)

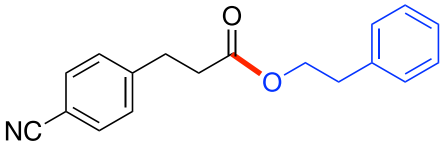

^{1}H NMR (600 MHz, CDCl$_3$) δ 7.60 – 7.56 (m, 2H), 7.33 (dd, *J* = 8.3, 6.8 Hz, 2H), 7.31 – 7.24 (m, 3H), 7.24 – 7.20 (m, 2H), 4.33 (t, *J* = 7.0 Hz, 2H), 3.00 (t, *J* = 7.5 Hz, 2H), 2.95 (t, *J* = 7.0 Hz, 2H), 2.66 (t, *J* = 7.6 Hz, 2H); **^{13}C NMR (101 MHz, CDCl$_3$) δ** 172.16, 146.10, 137.71, 132.35, 129.21, 128.91, 128.58, 126.69, 118.99, 110.26, 77.47, 65.18, 35.06, 30.89. **Yield:** 46%, 64.6 mg; white solid. **R$_f$** = 0.26 (85:15 hexanes/EtOAc). **Chemical Formula:** HRMS (ESI) *m/z*: [M+Na]$^+$ calcd. for $C_{18}H_{17}NO_2Na$ 302.1157; found 302.1161.

Phenethyl 3-(4-bromophenyl)propanoate (**25**)

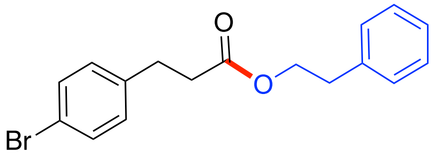

^{1}H NMR (600 MHz, CDCl$_3$) δ 7.43 – 7.38 (m, 2H), 7.35 – 7.29 (m, 2H), 7.28 – 7.23 (m, 1H), 7.23 – 7.18 (m, 2H), 7.07 – 7.02 (m, 2H), 4.31 (t, *J* = 7.0 Hz, 2H), 2.93 (t, *J* = 7.0 Hz, 2H), 2.89 (t, *J* = 7.7 Hz, 2H), 2.61 (t, *J* = 7.7 Hz, 2H); **^{13}C NMR (101 MHz, CDCl$_3$) δ** 172.57, 139.52, 137.83, 131.62, 130.18, 128.97, 128.60, 126.69, 120.15, 65.10, 35.69, 35.14, 30.35. **Yield:** 55%, 91.3 mg; white solid. **R$_f$** = 0.33 (90:10 hexanes/EtOAc). **Chemical Formula:** HRMS (ESI) *m/z*: [M+Na]$^+$ calcd. for $C_{17}H_{17}BrO_2Na$ 355.0310; found 355.0310.

Furan-2-ylmethyl dodecanoate (**26**)

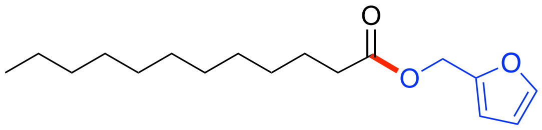

^{1}H NMR (400 MHz, CDCl$_3$) δ 7.42 (dd, *J* = 1.9, 0.9 Hz, 1H), 6.40 (d, *J* = 3.2 Hz, 1H), 6.36 (dd, *J* = 3.3, 1.8 Hz, 1H), 5.06 (s, 2H), 2.32 (t, *J* = 7.6 Hz, 2H), 1.68 – 1.56 (m, 2H), 1.34 – 1.22 (m, 16H), 0.88 (t, *J* = 6.8 Hz, 3H); **^{13}C NMR (101 MHz, CDCl$_3$) δ** 173.63, 149.79, 143.32, 110.66, 110.61, 57.99, 34.29, 32.04, 30.09, 29.74, 29.72, 29.58, 29.47, 29.37, 29.21, 25.01, 22.83, 14.26. **Yield:** 29%, 40.3 mg; yellow oil. **R$_f$** = 0.73 (90:10 hexanes/EtOAc). **Chemical Formula:** HRMS (ESI) *m/z*: [M+Na]$^+$ calcd. for $C_{17}H_{28}O_3Na$ 303.1936; found 303.1938.

4-Bromobenzyl pentanoate (**27**)

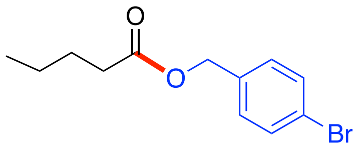

^{1}H NMR (600 MHz, $CDCl_3$) δ 7.51 – 7.46 (m, 2H), 7.25 – 7.20 (m, 2H), 5.05 (s, 2H), 2.35 (t, *J* = 7.6 Hz, 2H), 1.66 – 1.58 (m, 2H), 1.38 – 1.31 (m, 2H), 0.91 (t, *J* = 7.4 Hz, 3H); **^{13}C NMR (101 MHz, $CDCl_3$) δ** 173.68, 135.31, 131.82, 129.97, 122.32, 65.37, 34.12, 27.11, 22.38, 13.82. **Yield:** 50%, 27 mg; colorless oil. **R_f** = 0.30 (98:2 hexanes/EtOAc). Spectral data matched those previously reported.[3]

3-(4-Bromophenyl)propyl 3-(1H-indol-3-yl)propanoate (**28**)

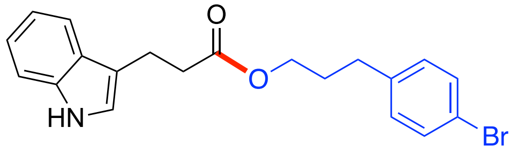

^{1}H NMR (600 MHz, $CDCl_3$) 7.87 (s, 1H), 7.54 (dd, *J* = 7.9, 1.2 Hz, 1H), 7.32 – 7.16 (m, 3H), 7.12 (ddd, *J* = 8.2, 7.0, 1.2 Hz, 1H), 7.05 (ddd, *J* = 8.0, 7.0, 1.0 Hz, 1H), 6.93 (d, *J* = 2.3 Hz, 1H), 6.92 – 6.87 (m, 2H), 3.99 (t, *J* = 6.4 Hz, 2H), 3.06 – 3.01 (m, 2H), 2.65 (t, *J* = 7.6 Hz, 2H), 2.47 (dd, *J* = 8.6, 6.8 Hz, 2H), 1.83 – 1.76 (m, 2H); **^{13}C NMR (101 MHz, $CDCl_3$) δ** 173.53, 140.28, 136.40, 131.57, 130.28, 127.30, 122.23, 121.54, 119.84, 119.50, 118.85, 115.08, 111.27, 63.59, 35.04, 31.65, 30.14, 20.83. **Yield:** 75%, 77 mg; white solid. **R_f** = 0.34 (85:15 hexanes/EtOAc). **Chemical Formula:** HRMS (ESI) *m/z*: $[M+Na]^+$ calcd. for $C_{20}H_{20}BrNO_2Na$ 408.0571; found 408.0575.

n-Octyl pentanoate (**33**)

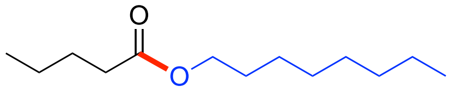

^{1}H NMR (500 MHz, $CDCl_3$) δ 4.06 (t, *J* = 6.7 Hz, 2H), 2.30 (t, *J* = 7.5 Hz, 2H), 1.66 – 1.56 (m, 4H), 1.39 – 1.21 (m, 12H), 0.90 (dt, *J* = 16.6, 7.2 Hz, 6H)**; ^{13}C NMR (126 MHz, $CDCl_3$) δ** 173.97, 64.38, 34.11,

[3] Janssen, P. G. A.; Pouderoijen, M.; van Breemen, A. J. J. M.; Herwig, P. T.; Koeckelberghs, G.; Popa-Merticaru, A. R.; Meskers, S. C. J.; Valeton, J. J. P.; Meijer, E. W.; Schenning, A. H. J. Synthesis and Properties of α,ω-Phenyl-Capped Bithiophene Derivatives. *J. Mater. Chem.* **2006**, *16*, 4335–4342.

31.77, 29.20, 29.18, 28.65, 27.10, 25.92, 22.63, 22.27, 14.06, 13.70. **Yield:** 86%, 43 mg; colorless liquid. **R$_f$** = 0.65 (95:5 hexanes/EtOAc). Spectral data matched those previously reported. [4]

4-Hydroxyphenethyl dodecanoate (**36**)

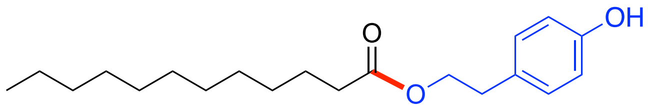

^{1}H NMR (400 MHz, CDCl$_3$) δ 7.10 – 7.03 (m, 2H), 6.80 – 6.74 (m, 2H), 4.25 (t, *J* = 7.1 Hz, 2H), 2.86 (t, *J* = 7.1 Hz, 2H), 2.35 (t, *J* = 7.5 Hz, 1H), 2.29 (t, *J* = 7.5 Hz, 2H), 1.70 – 1.53 (m, 3H), 1.26 (d, *J* = 3.9 Hz, 22H), 0.88 (t, *J* = 6.8 Hz, 4H). **^{13}C NMR (126 MHz, CDCl$_3$) δ** 180.20, 174.24, 154.44, 130.17, 115.48, 65.17, 34.52, 34.41, 34.19, 32.05, 29.76, 29.74, 29.60, 29.57, 29.49, 29.47, 29.41, 29.38, 29.27, 29.20, 25.09, 24.83, 22.83, 14.25. **Yield:** 37%, 36.5 mg; white solid. **R$_f$** = 0.73 (80:20 hexanes/EtOAc). **Chemical Formula:** HRMS (ESI) *m/*z: [M+Na]$^+$ calcd. for $C_{20}H_{32}O_3Na$ 343.2249; found 343.2253. Sample contains some hexanes.

2-Hydroxy-3-(2-methoxyphenoxy)propyl dodecanoate (**38**)

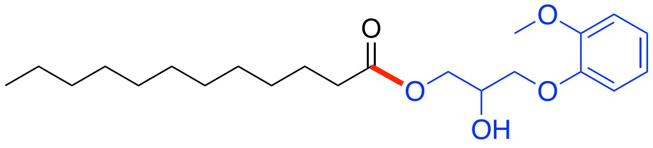

^{1}H NMR (400 MHz, CDCl$_3$) δ 7.03 – 6.87 (m, 4H), 4.33 – 4.17 (m, 3H), 4.12 – 3.93 (m, 2H), 3.86 (s, 3H), 2.34 (t, *J* = 7.6 Hz, 2H), 1.67 – 1.57 (m, 2H), 1.30 – 1.21 (m, 16H), 0.88 (t, *J* = 6.8 Hz, 3H). **^{13}C NMR (126 MHz, CDCl$_3$) δ** 173.96, 150.03, 147.96, 122.56, 121.05, 115.73, 112.04, 71.50, 68.49, 64.95, 55.82, 34.15, 31.90, 29.59, 29.44, 29.32, 29.25, 29.13, 24.91, 22.67, 14.11. **Yield:** 19%, 35.2 mg; white solid. **R$_f$** = 0.35 (70:30 hexanes/EtOAc). **Chemical Formula:** HRMS (ESI) *m/z*: [M+Na]$^+$ calcd. for $C_{22}H_{36}O_5Na$ 403.2460; found 403.2447.

Octyl 3-(4'-methoxy-2'-methyl-[1,1'-biphenyl]-4-yl)propanoate (**43**)

[4] Cahiez, G.; Chaboche, C.; JeÂzeÂquel, M. Cu-Catalyzed Alkylation of Grignard Reagents: A New Efficient Procedure. *Tetrahedron* **2000**, *56*, 2733-2737.

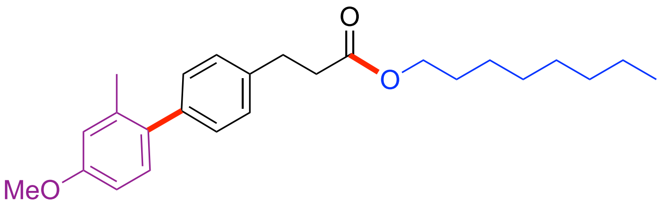

^{1}H NMR (500 MHz, $CDCl_3$) δ 7.24 (s, 4H), 7.15 (d, *J* = 8.3 Hz, 1H), 6.83 (d, *J* = 2.7 Hz, 1H), 6.80 (dd, *J* = 8.3, 2.7 Hz, 1H), 4.10 (t, *J* = 6.8 Hz, 2H), 3.84 (s, 3H), 3.01 (dd, *J* = 8.5, 7.3 Hz, 2H), 2.69 (dd, *J* = 8.5, 7.2 Hz, 2H), 2.27 (s, 3H), 1.68 – 1.57 (m, 2H), 1.40 – 1.23 (m, 10H), 0.93 – 0.87 (m, 3H); **^{13}C NMR (126 MHz, $CDCl_3$) δ** 173.2, 158.8, 139.7, 138.9, 136.9, 134.5, 131.0, 129.6, 128.1, 115.8, 111.2, 64.8, 55.4, 36.0, 31.9, 30.8, 29.3, 29.3, 28.8, 26.0, 22.8, 20.9, 14.2. **Yield:** 82%, 157.2mg, colorless oil. **R_f** = 0.50 (95:5 hexanes/EtOAc). **Chemical Formula**: HRMS (ESI) *m/z*: $[M+Na]^+$ calcd. for $C_{25}H_{34}O_3Na$ 405.2406; found 405.2404.

2-(1-Methylcyclopropyl)ethyl 3-phenylpropanoate (**47**)

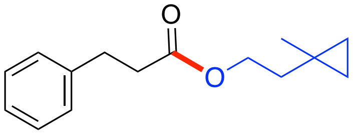

^{1}H NMR (500 MHz, $CDCl_3$) δ 7.31 – 7.27 (m, 2H), 7.22 – 7.18 (m, 3H), 4.18 (t, *J* = 7.1 Hz, 2H), 2.95 (t, *J* = 7.9 Hz, 2H), 2.64 – 2.60 (m, 2H), 1.54 (t, *J* = 7.1 Hz, 2H), 1.04 (s, 3H), 0.31 – 0.23 (m, 4H); **^{13}C NMR (126 MHz, $CDCl_3$) δ** 173.09, 140.73, 128.62, 128.43, 126.37, 63.35, 38.00, 36.14, 31.11, 22.90, 13.09, 12.84. **Yield**: 65%, 75.4 mg, colorless oil. **R_f** = 0.60 (85:15 hexanes/EtOAc). Spectral data matched those previously reported.[5]

(*R*)-5-(4-(1-Hydroxyethyl)phenyl)pent-4-yn-1-yl 3-phenylpropanoate (**52**)

[5] Wood, A. B.; Cortes-Clerget, M.; Kincaid, J. R. A.; Akkachairin, B.; Singhania, V.; Gallou, F.; and Lipshutz, B.H. Nickel Nanoparticle Catalyzed Mono- and Di-Reductions of Gem-Dibromocyclopropanes Under Mild, Aqueous Micellar Conditions. *Angew. Chem., Int. Ed.* **2020**, *59*, 17587-17593.

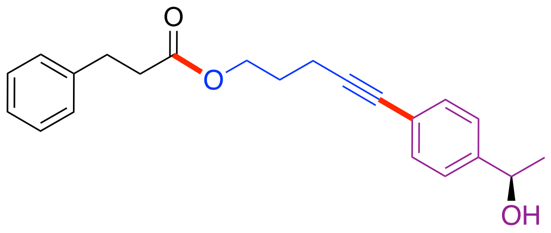

^{1}H NMR (500 MHz, $CDCl_3$) δ 7.37 (d, *J* = 8.1 Hz, 2H), 7.29 (d, 4H), 7.23-7.16 (m, 3H), 4.37 (q, *J* = 6.4 Hz, 1H), 4.22 (t, *J* = 6.3 Hz, 2H), 2.96 (t, *J* = 7.8 Hz, 2H), 2.65 (t, *J* = 7.8 Hz, 2H), 2.45 (t, *J* = 7.0 Hz, 2H), 1.90 (p, *J* = 6.7 Hz, 2H), 1.47 (d, *J* = 6.5 Hz, 3H), 1.27 (s, 1H); **^{13}C NMR (126 MHz, $CDCl_3$) δ** 172.9, 145.4, 140.5, 131.7, 128.5, 128.3, 126.3, 125.3, 122.8, 88.5, 81.1, 70.1, 63.2, 35.9, 40.0, 27.8, 25.1, 16.2. **Yield**: 59%, 40 mg, colorless oil. **R_f** = 0.3 (85:15 hexanes/EtOAc). **Chemical Formula:** HRMS (ESI) *m/z*: $[M+Na]^+$ calcd. for $C_{22}H_{24}O_3Na$ 318.1620; found 318.1627.

3-(4-Ethylphenyl)propyl 3-(1H-indol-3-yl)propanoate (**56**)

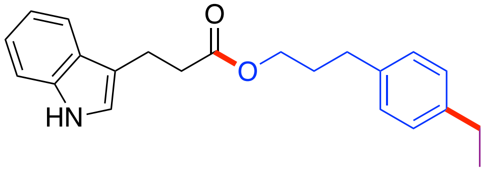

^{1}H NMR (400 MHz, $CDCl_3$) δ 7.93 (s, 1H), 7.61 (d, *J* = 7.8 Hz, 1H), 7.34 (d, *J* = 8.1 Hz, 1H), 7.22 – 7.07 (m, 4H), 7.04 (d, *J* = 7.8 Hz, 2H), 7.00 (s, 1H), 4.09 (t, *J* = 6.5 Hz, 2H), 3.11 (t, *J* = 7.7 Hz, 2H), 2.72 (t, *J* = 7.6 Hz, 2H), 2.60 (dt, *J* = 12.2, 7.5 Hz, 4H), 1.90 (p, *J* = 6.9 Hz, 2H), 1.21 (t, *J* = 7.6 Hz, 3H).**^{13}C NMR (101 MHz, $CDCl_3$) δ** 173.42, 141.85, 138.36, 136.27, 128.30, 127.87, 127.19, 122.07, 121.35, 119.34, 118.72, 115.06, 111.10, 77.20, 63.79, 34.93, 31.66, 30.24, 28.41, 20.68, 15.62. **Yield:** 46%, 39 mg, yellow oil. **R_f** = 0.21 (90:10 hexanes/EtOAc). **Chemical Formula:** HRMS (ESI) *m/z*: $[M+Na]^+$ calcd. for $C_{22}H_{25}NO_2Na$ 358.1783; found 358.1789.

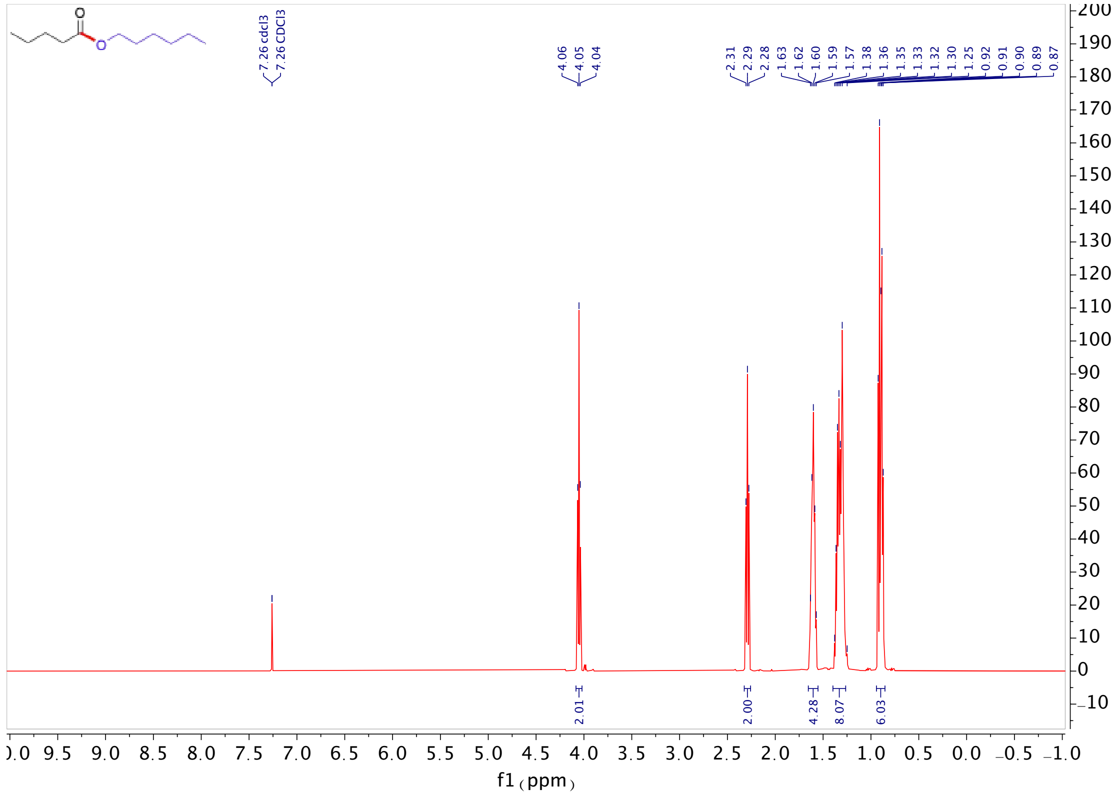

^{1}H NMR spectrum of *n*-Hexyl pentanoate (**3**)

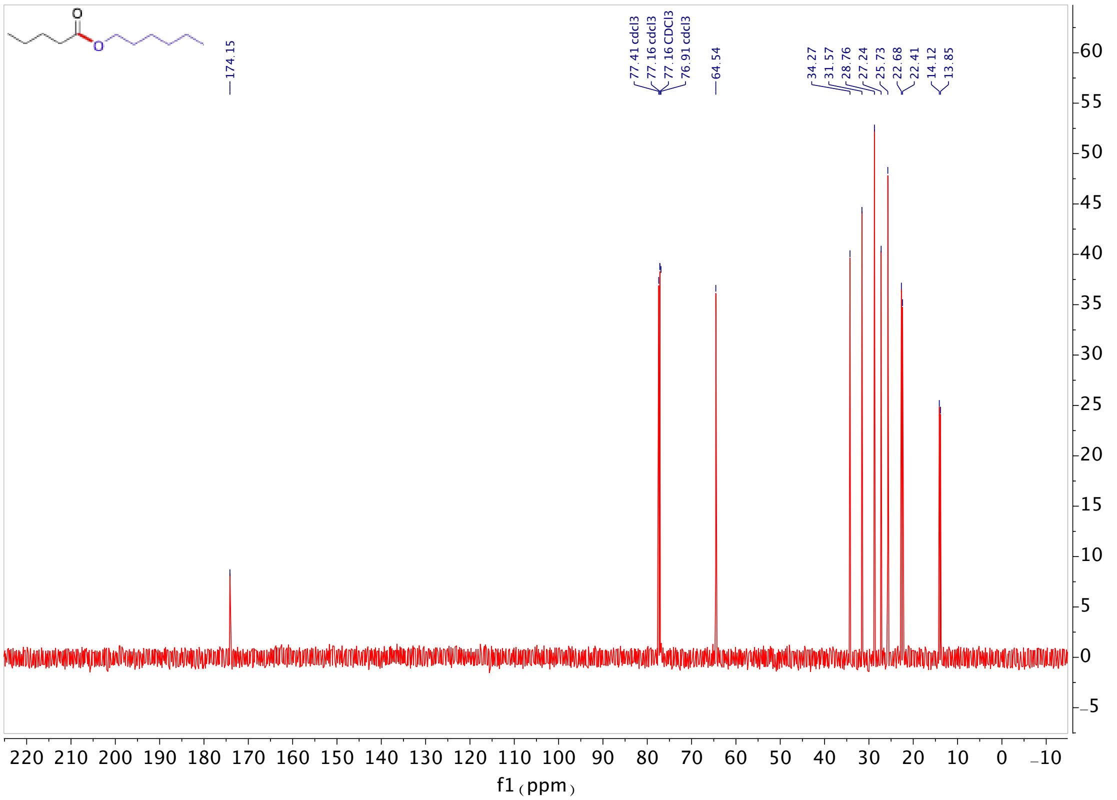

^{13}C NMR spectrum of *n*-Hexyl pentanoate (**3**)

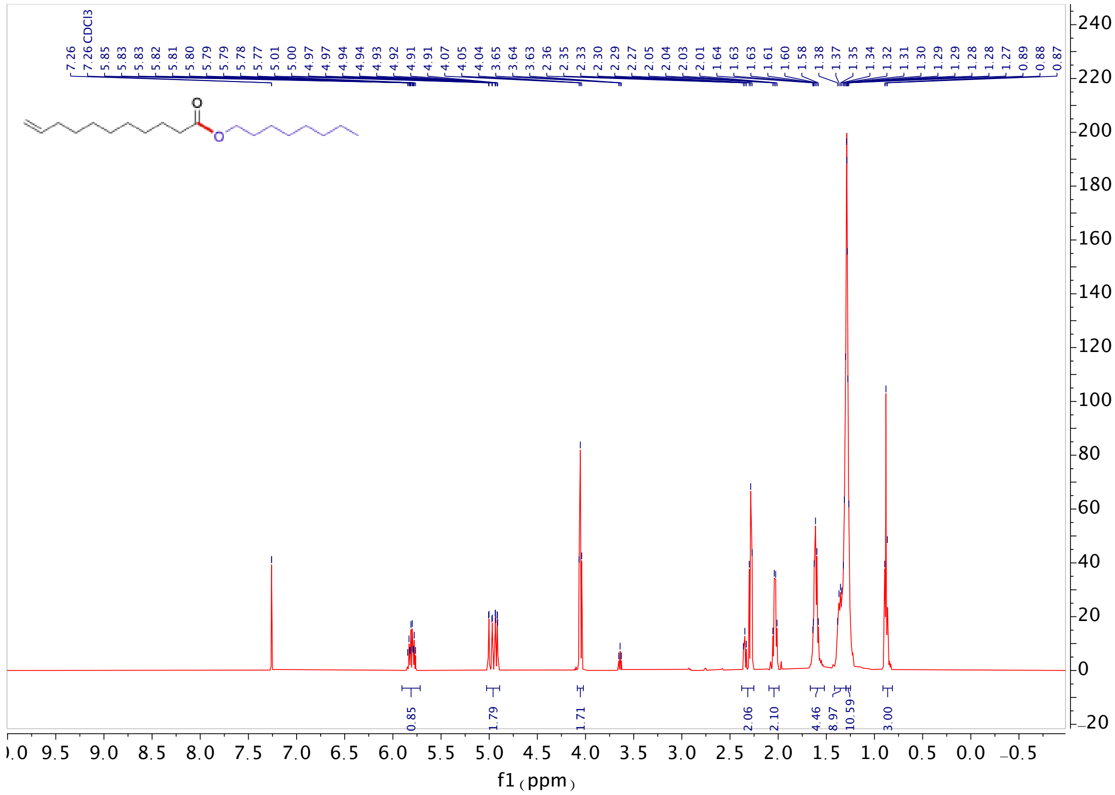

^{1}H NMR spectrum of *n*-Octyl undec-10-enoate (**4**)

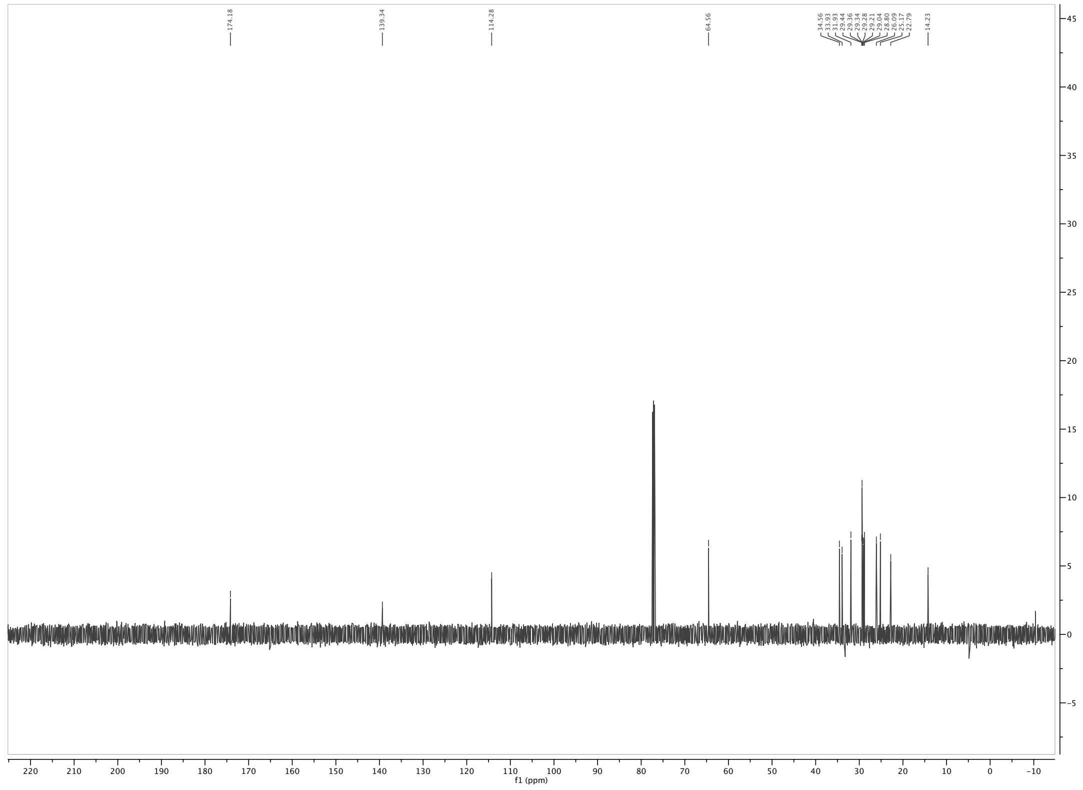

^{13}C NMR spectrum of *n*-Octyl undec-10-enoate (**4**)

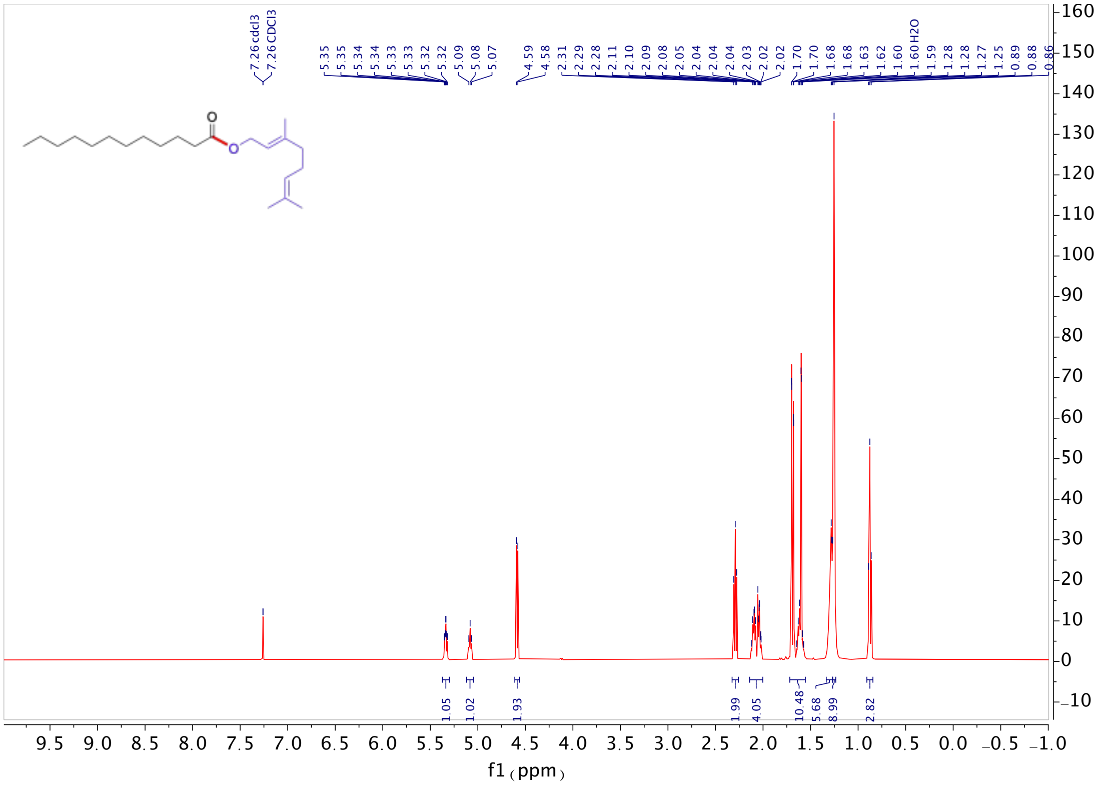

^{1}H NMR spectrum of (*E*)-3,7-Dimethylocta-2,6-dien-1-yl dodecanoate (**5**)

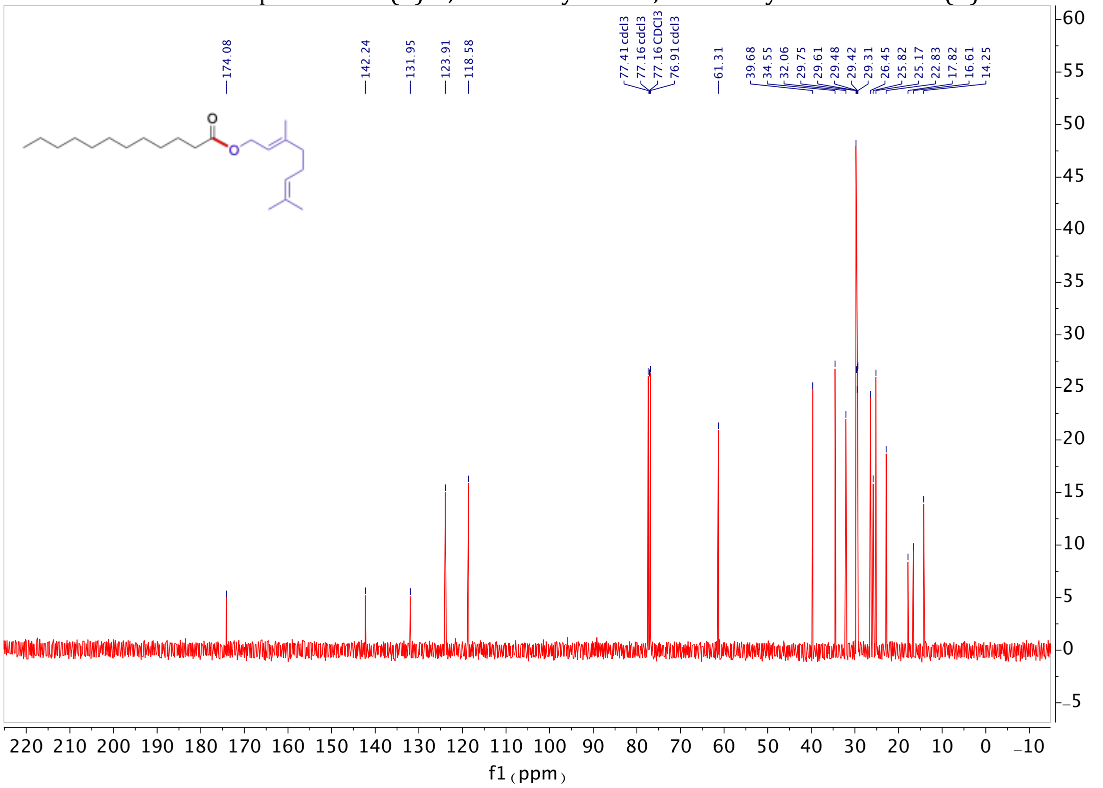

^{13}C NMR spectrum of (*E*)-3,7-Dimethylocta-2,6-dien-1-yl dodecanoate (**5**)

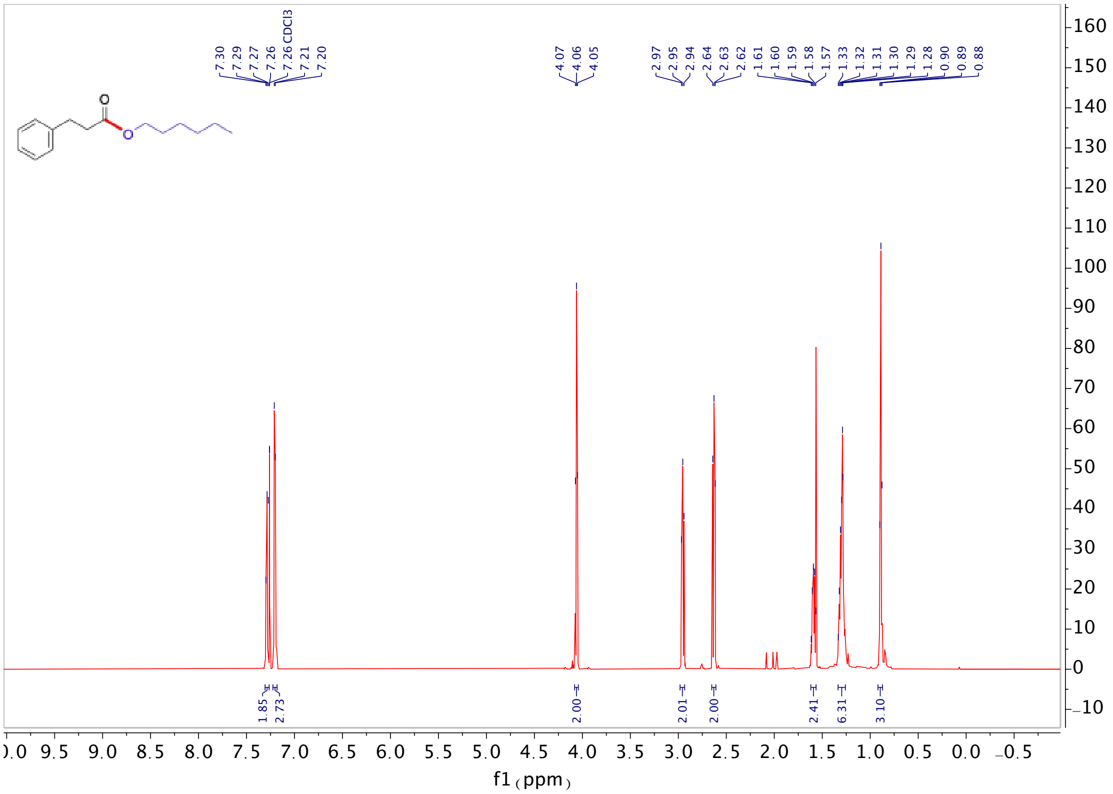

^{1}H NMR spectrum of *n*-Hexyl 3-phenylpropanoate (**6**)

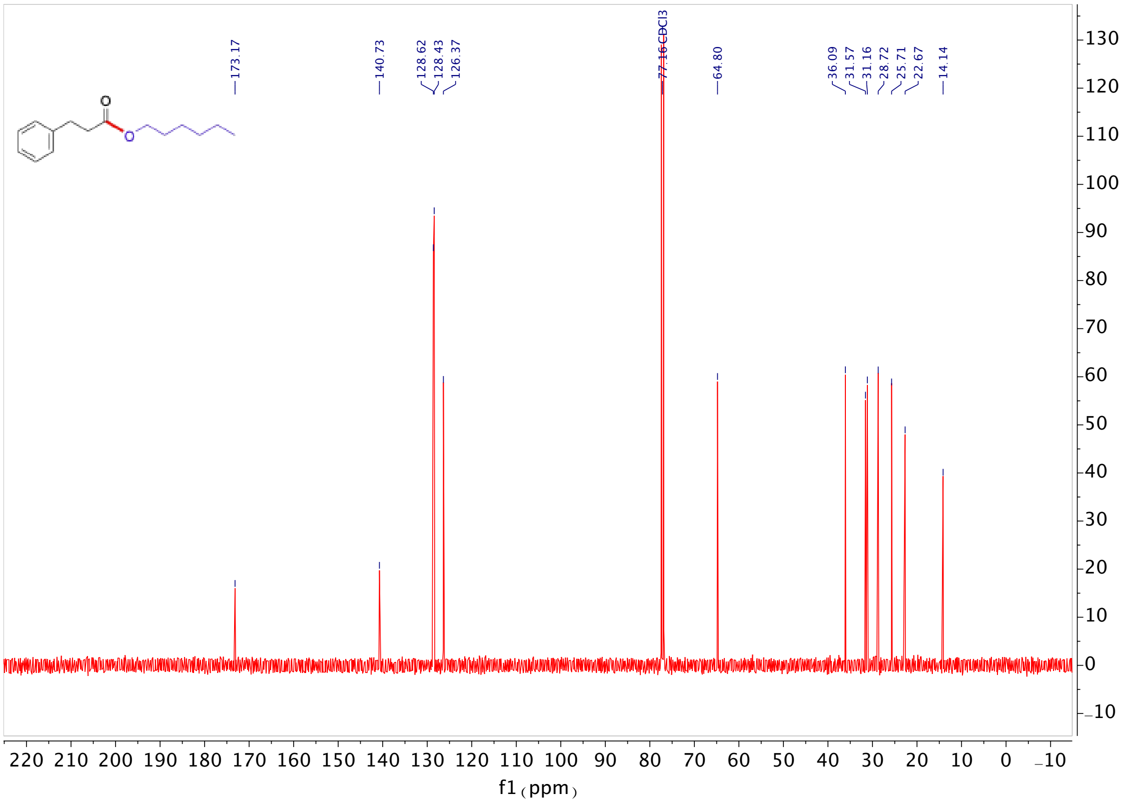

^{13}C NMR spectrum of *n*-Hexyl 3-phenylpropanoate (**6**)

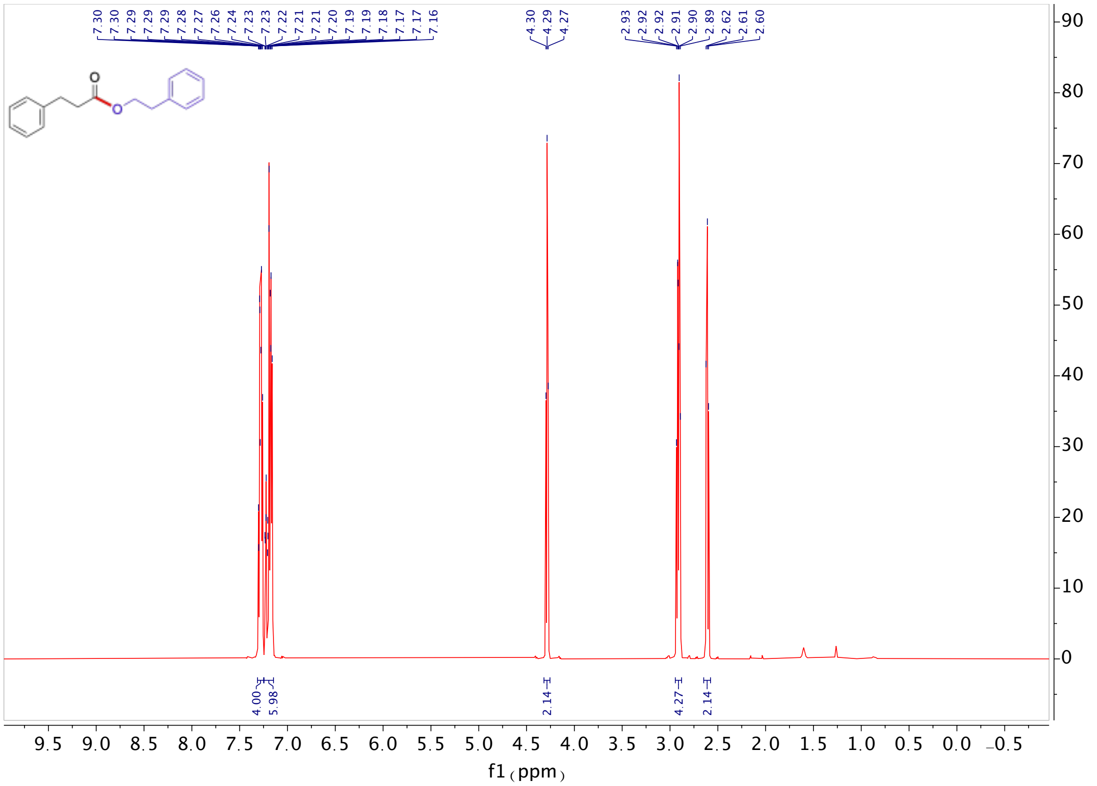

^{1}H NMR spectrum of Phenethyl 3-phenylpropanoate (**7**)

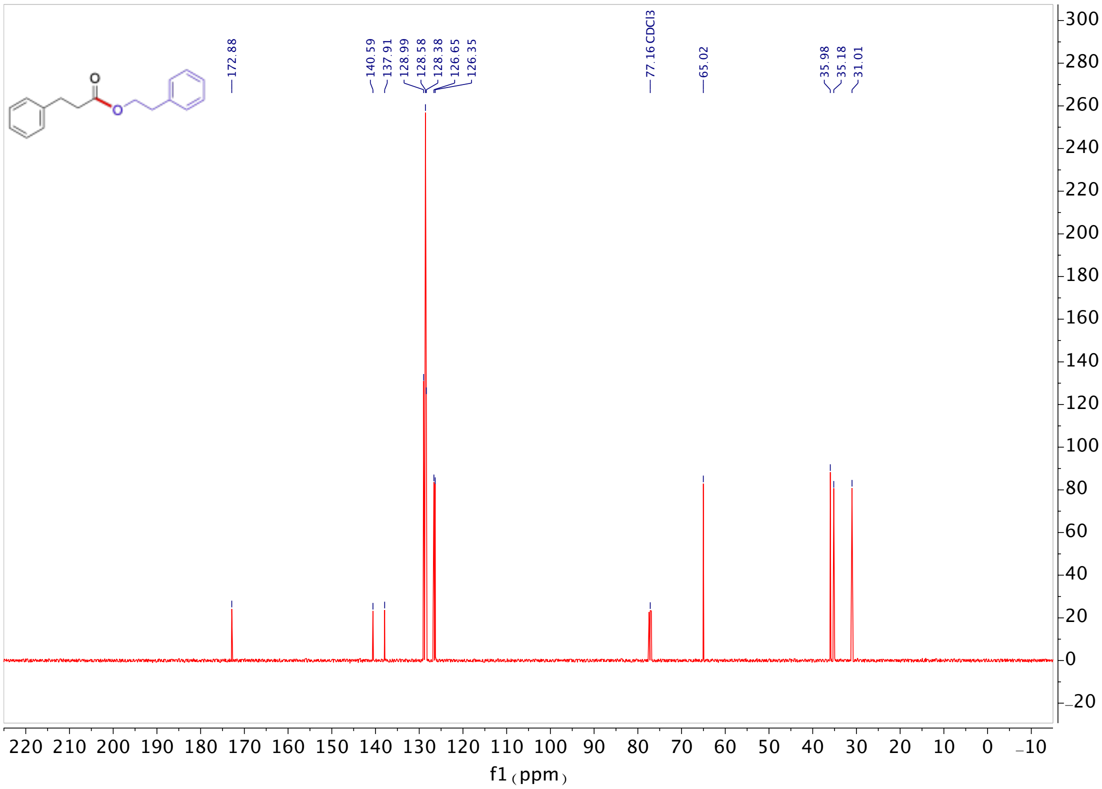

^{13}C NMR spectrum of Phenethyl 3-phenylpropanoate (**7**)

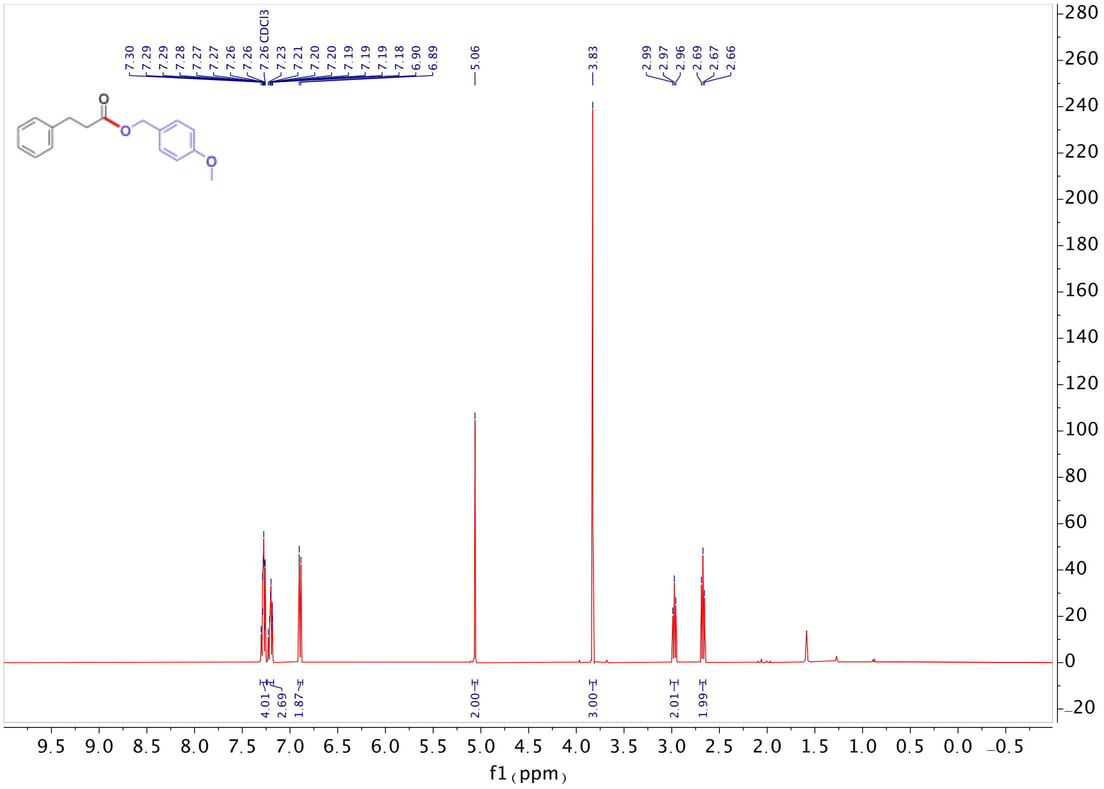

^{1}H NMR spectrum of 4-Methoxybenzyl 3-phenylpropanoate (**8**)

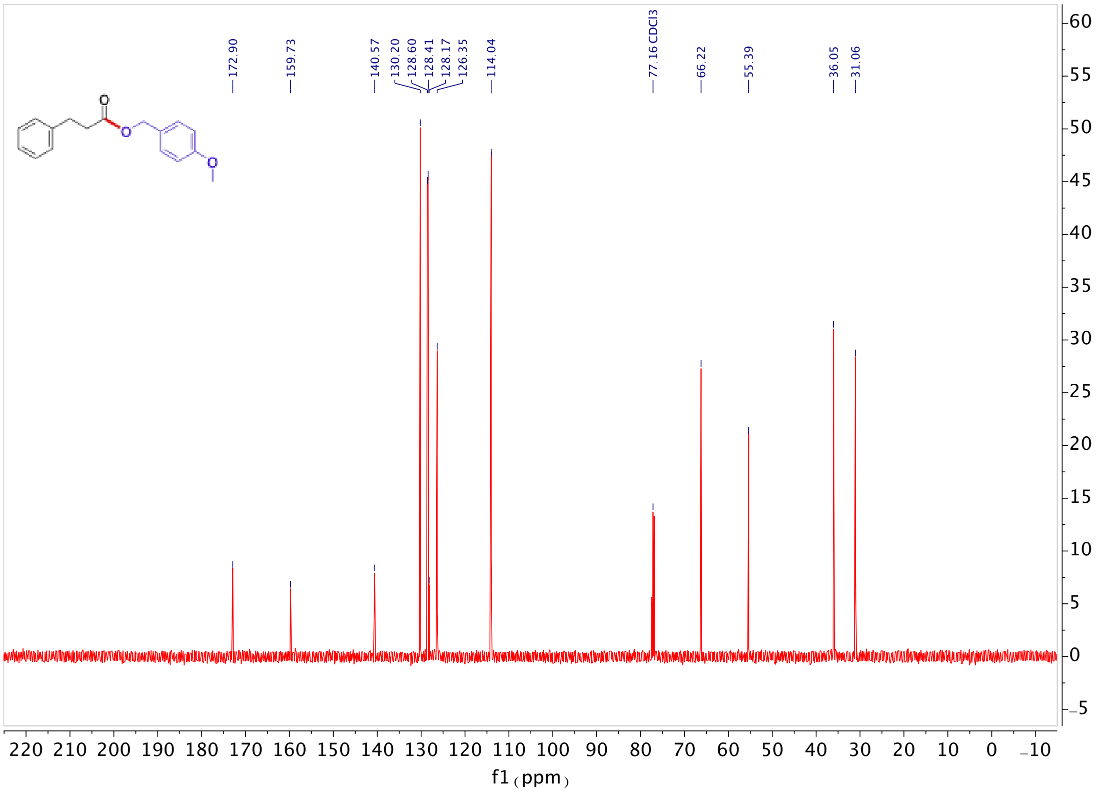

^{13}C NMR spectrum of 4-Methoxybenzyl 3-phenylpropanoate (**8**)

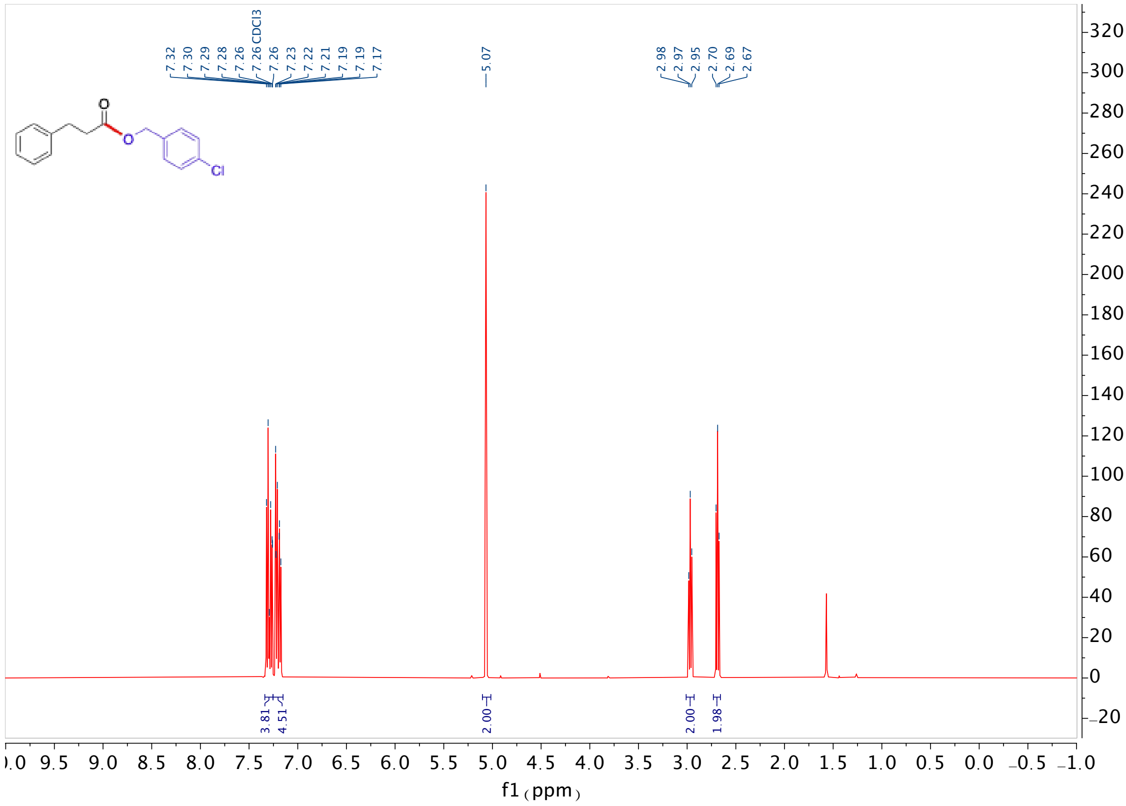

^{1}H NMR spectrum of 4-Chlorobenzyl 3-phenylpropanoate (**9**)

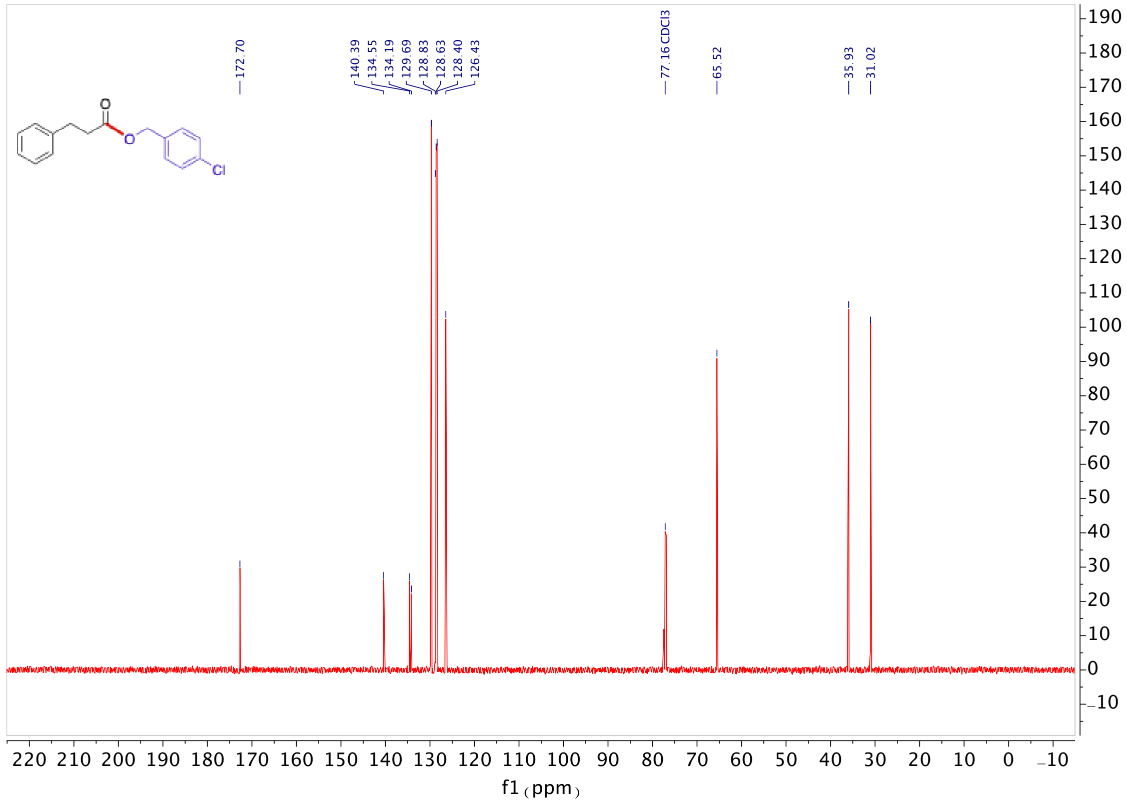

^{13}C NMR spectrum of 4-Chlorobenzyl 3-phenylpropanoate (**9**)

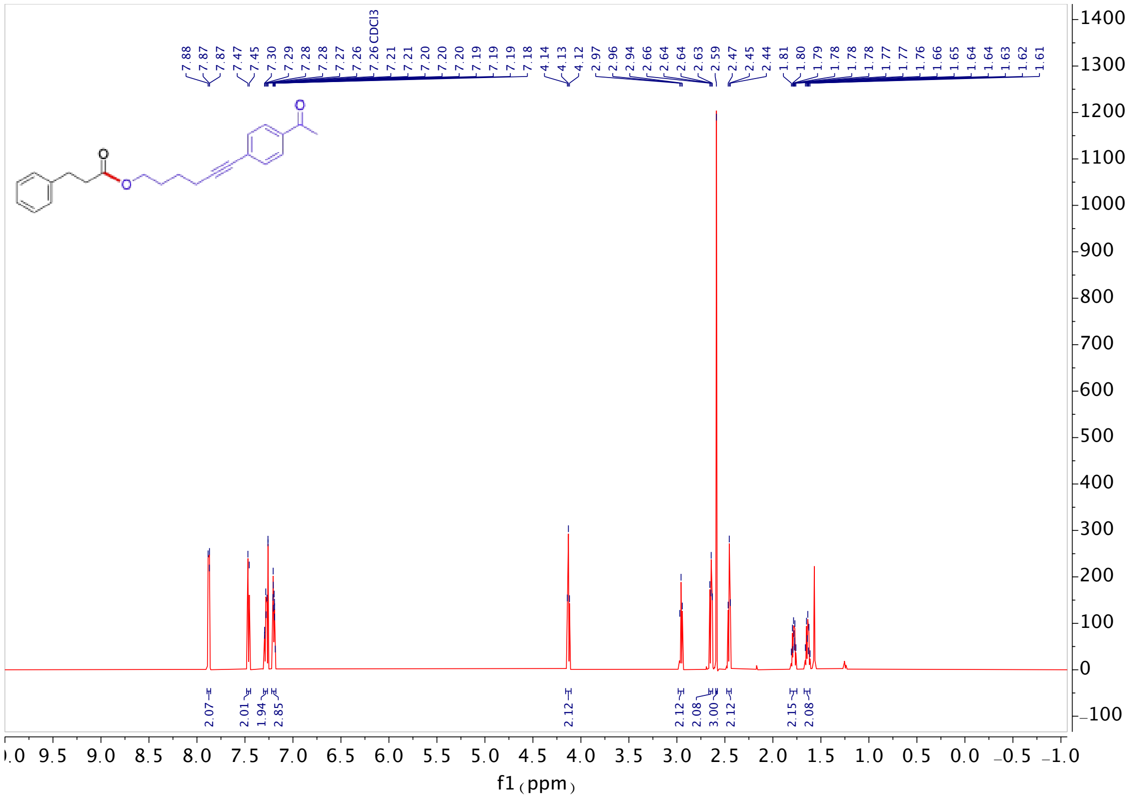

^{1}H NMR spectrum of 6-(4-Acetylphenyl)hex-5-yn-1-yl 3-phenylpropanoate (**10**)

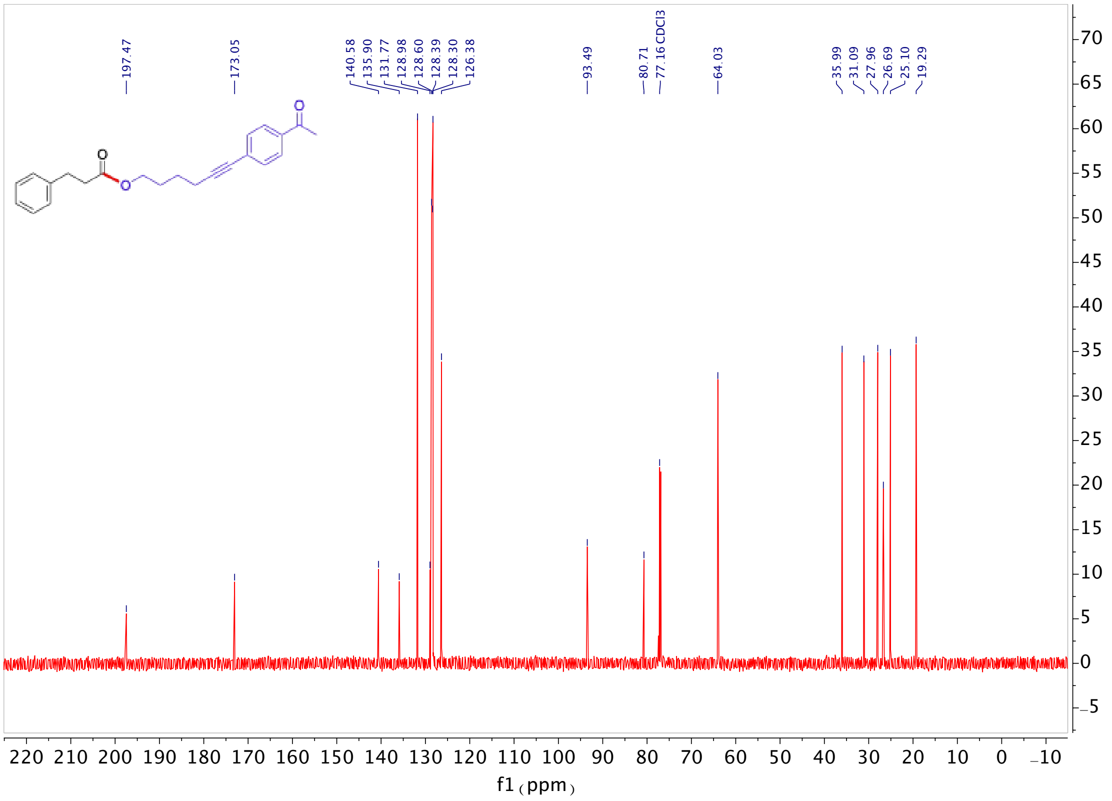

^{13}C NMR spectrum of 6-(4-Acetylphenyl)hex-5-yn-1-yl 3-phenylpropanoate (**10**)

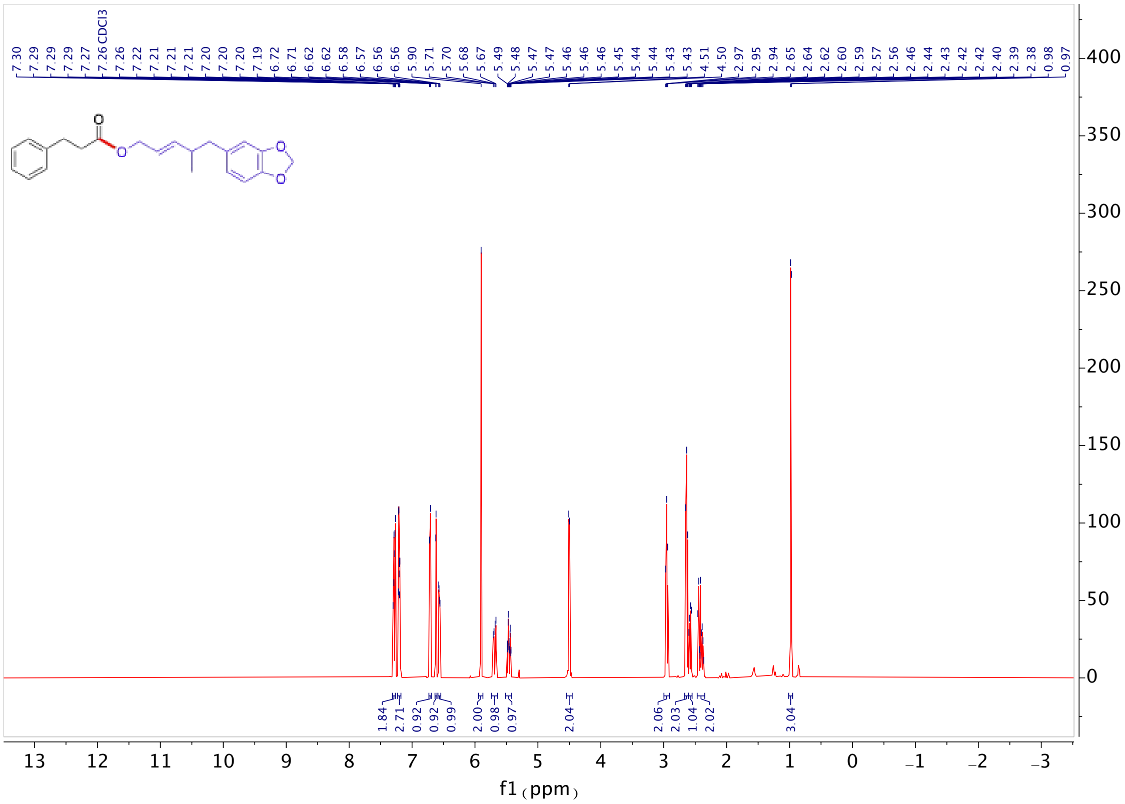

^{1}H NMR spectrum of (*E*)-5-(Benzo[d][1,3]dioxol-5-yl)-4-methylpent-2-en-1-yl 3-phenylpropanoate (**11**)

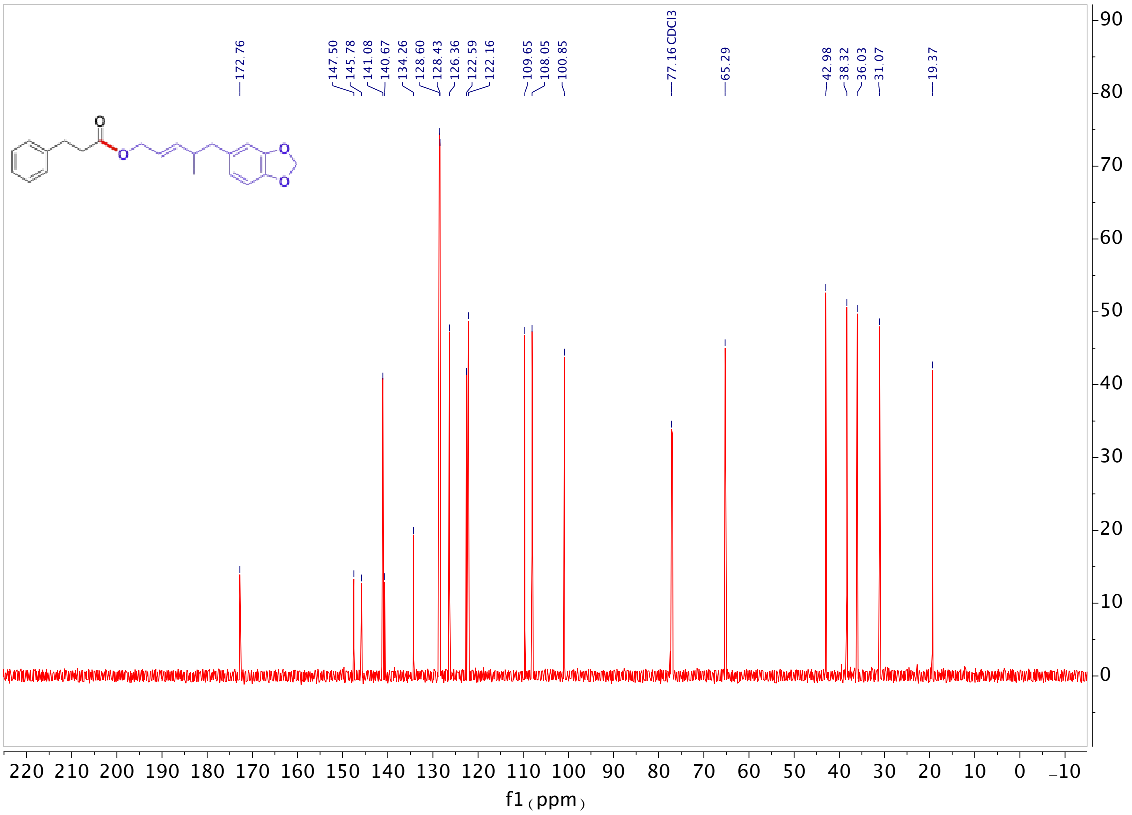

^{13}C NMR spectrum of (*E*)-5-(Benzo[d][1,3]dioxol-5-yl)-4-methylpent-2-en-1-yl 3-phenylpropanoate (**11**)

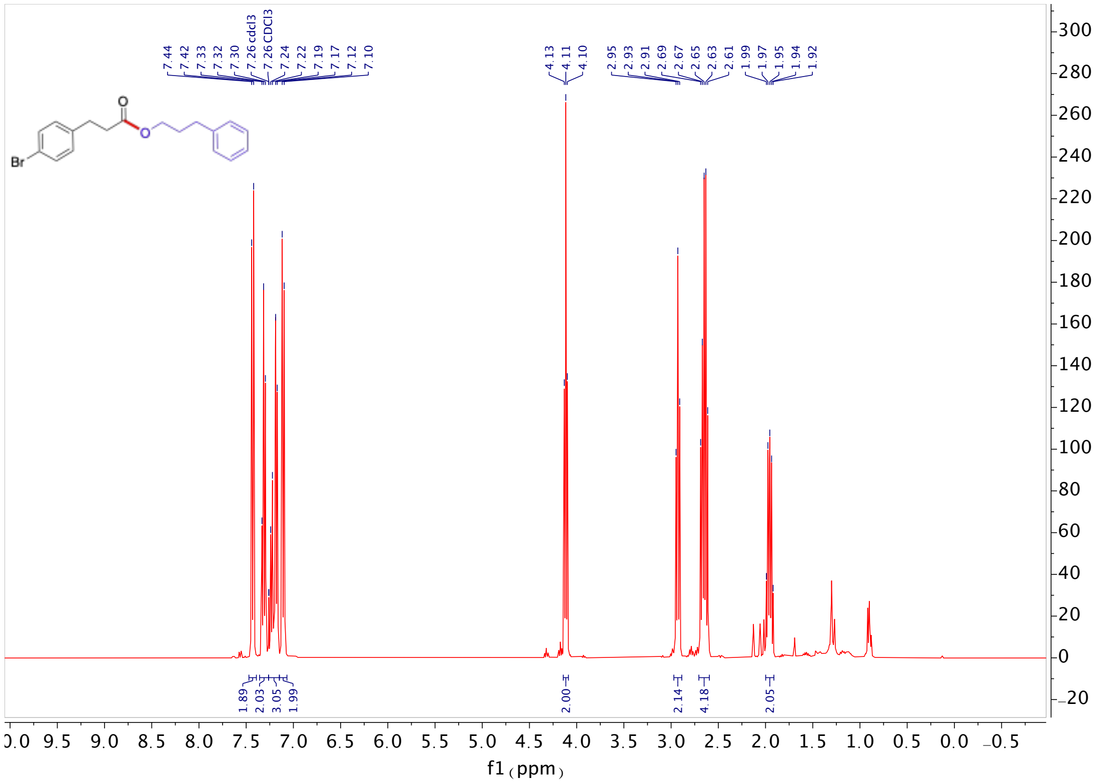

^{1}H NMR spectrum of 3-Phenylpropyl 3-(4-bromophenyl)propanoate (**12**)

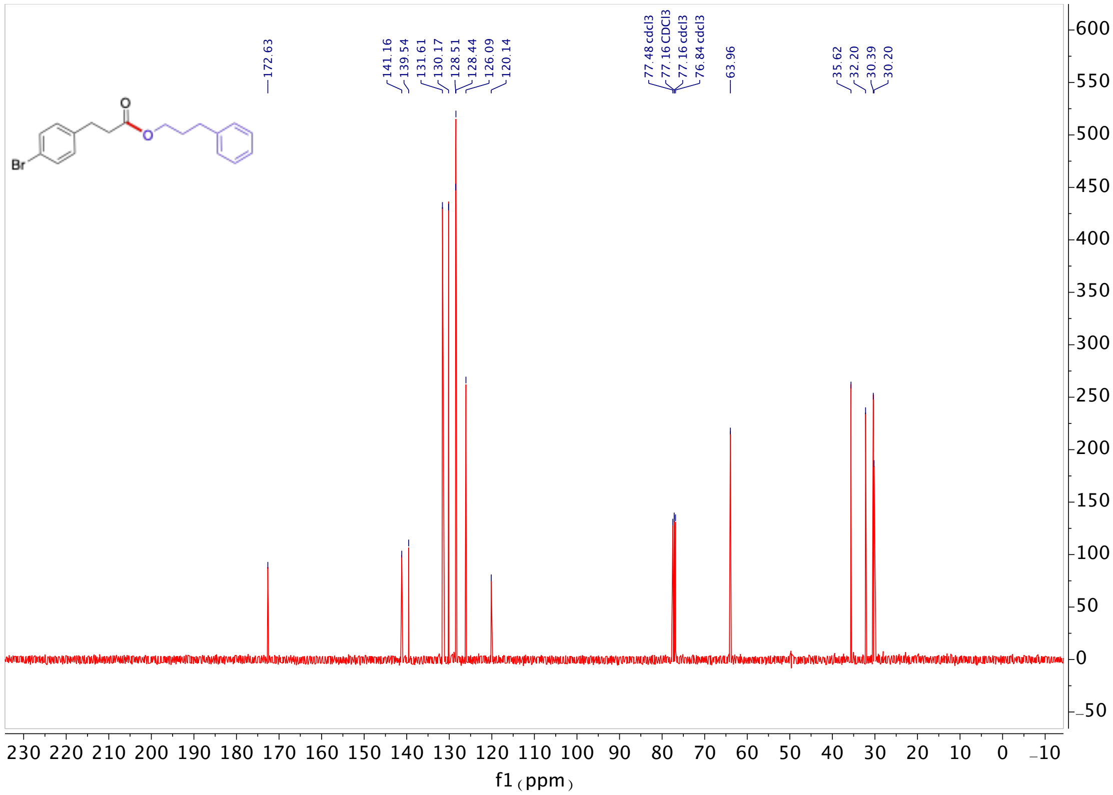

^{13}C NMR spectrum of 3-Phenylpropyl 3-(4-bromophenyl)propanoate (**12**)

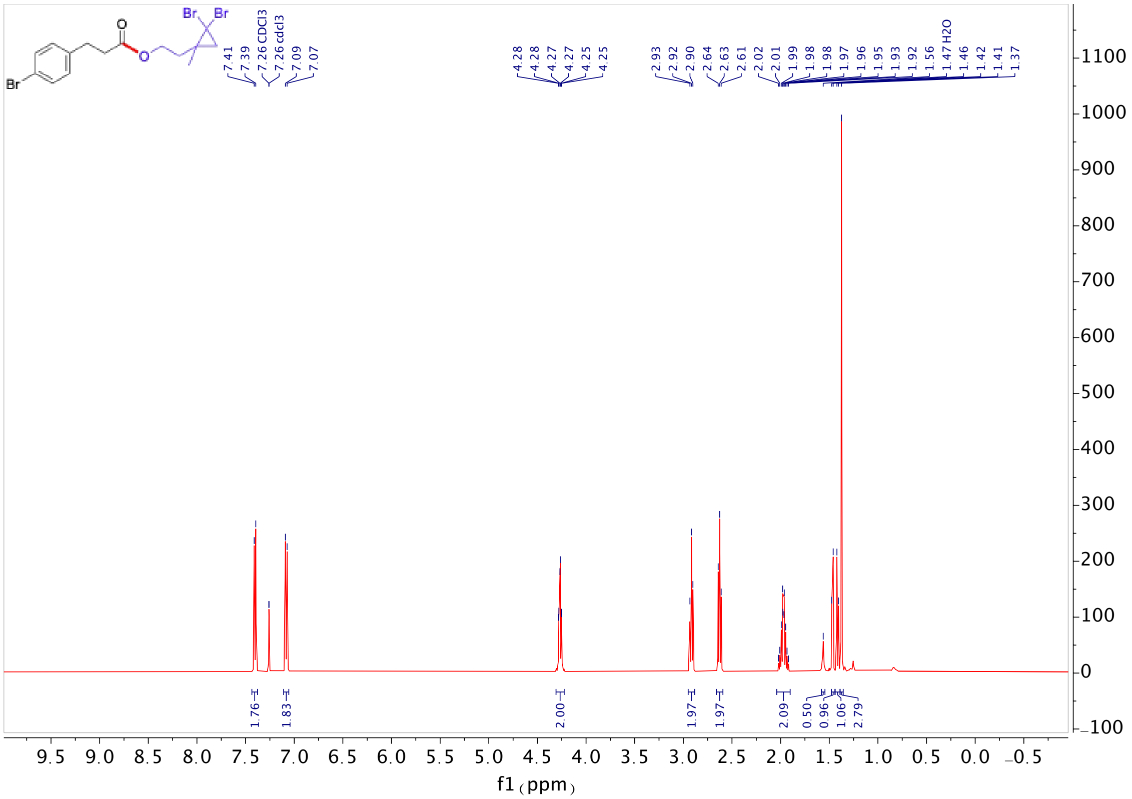

^{1}H NMR spectrum of 2-(2,2-Dibromo-1-methylcyclopropyl)ethyl 3-(4-bromophenyl)propanoate (**13**)

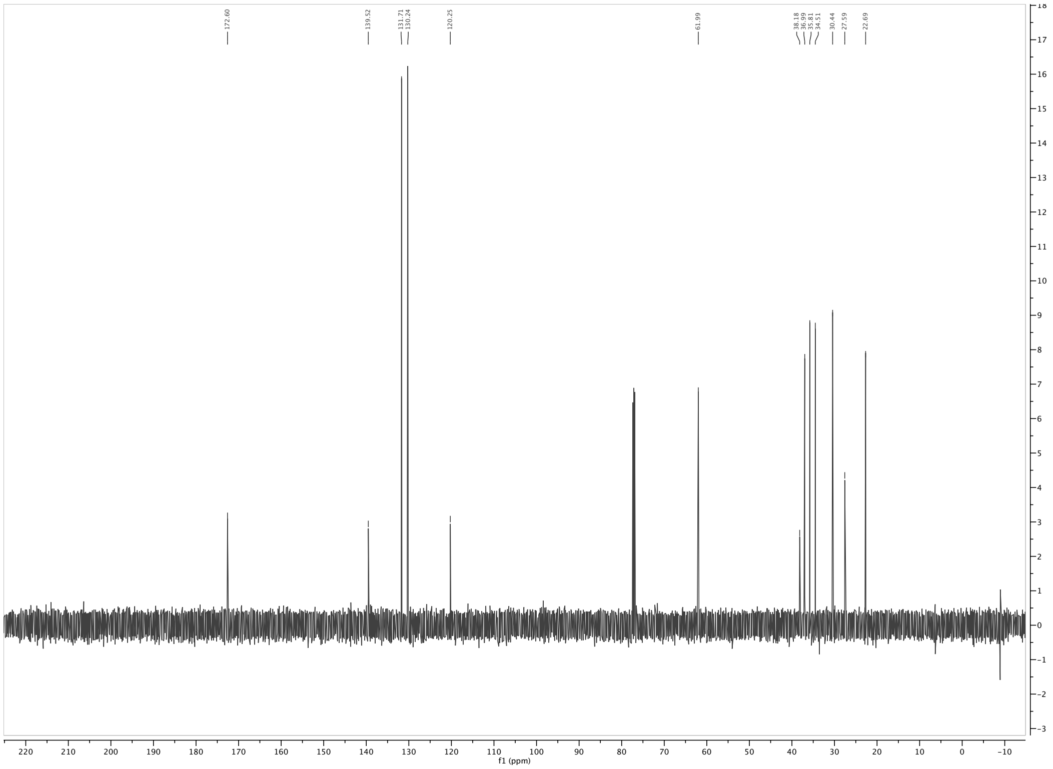

^{13}C NMR spectrum of 2-(2,2-Dibromo-1-methylcyclopropyl)ethyl 3-(4-bromophenyl)propanoate (**13**)

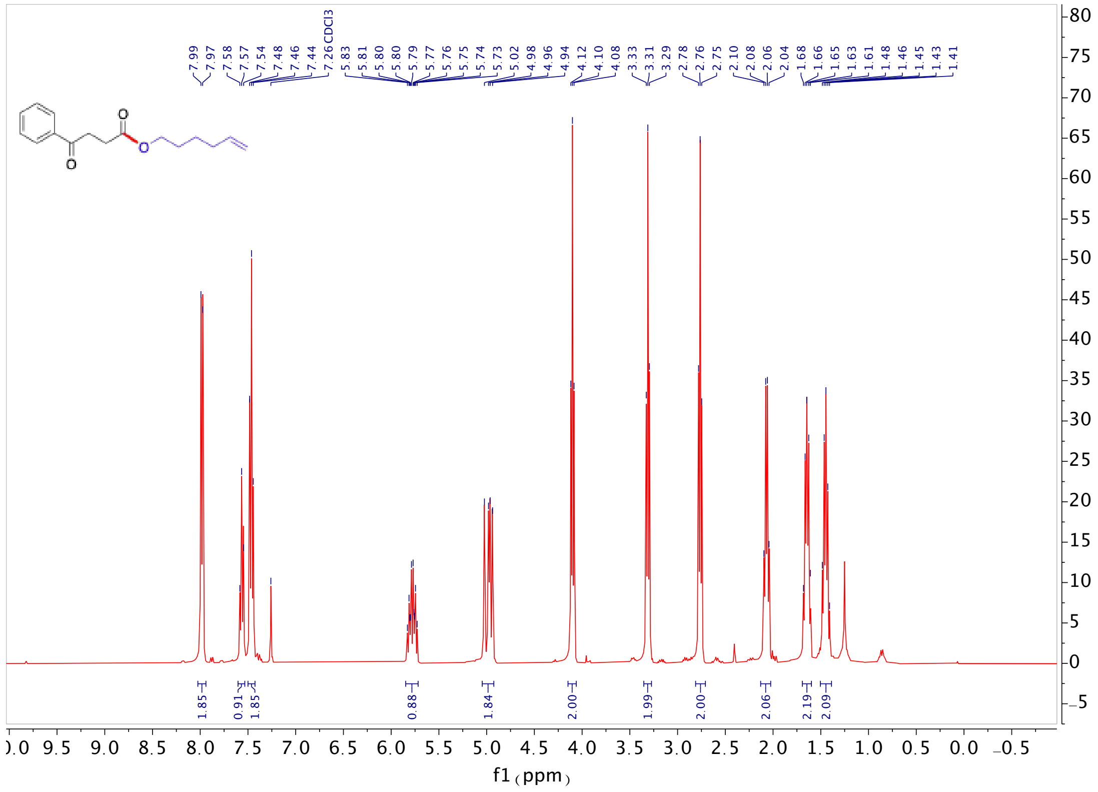

^{1}H NMR spectrum of Hex-5-en-1-yl 4-oxo-4-phenylbutanoate (**14**)

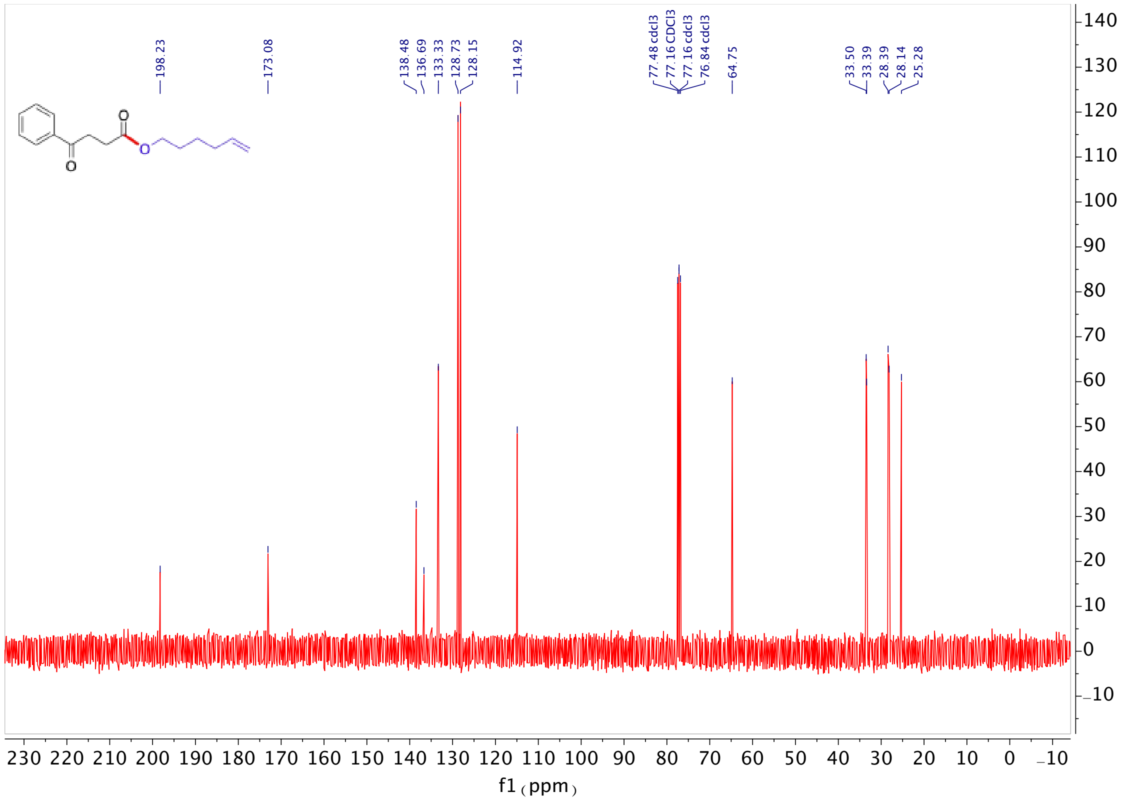

^{13}C NMR spectrum of Hex-5-en-1-yl 4-oxo-4-phenylbutanoate (**14**)

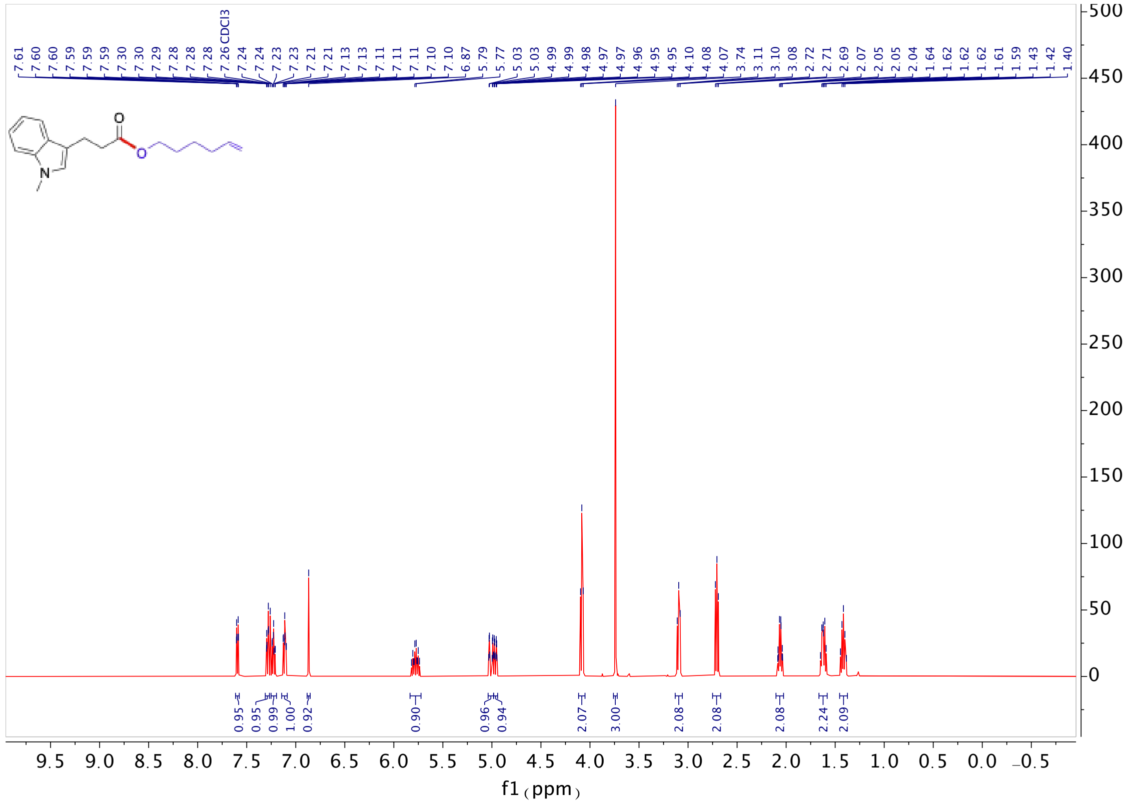

^{1}H NMR spectrum of Hex-5-en-1-yl 3-(1-methyl-1H-indol-3-yl)propanoate (**15**)

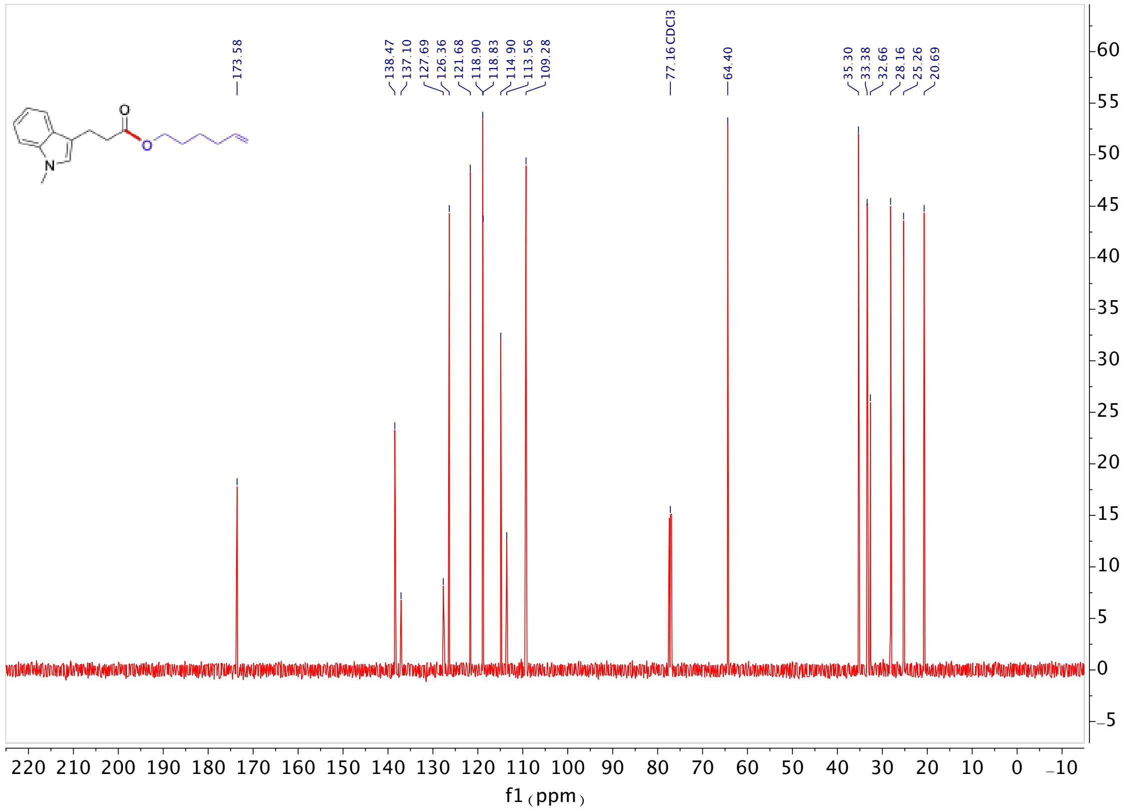

^{13}C NMR spectrum of Hex-5-en-1-yl 3-(1-methyl-1H-indol-3-yl)propanoate (**15**)

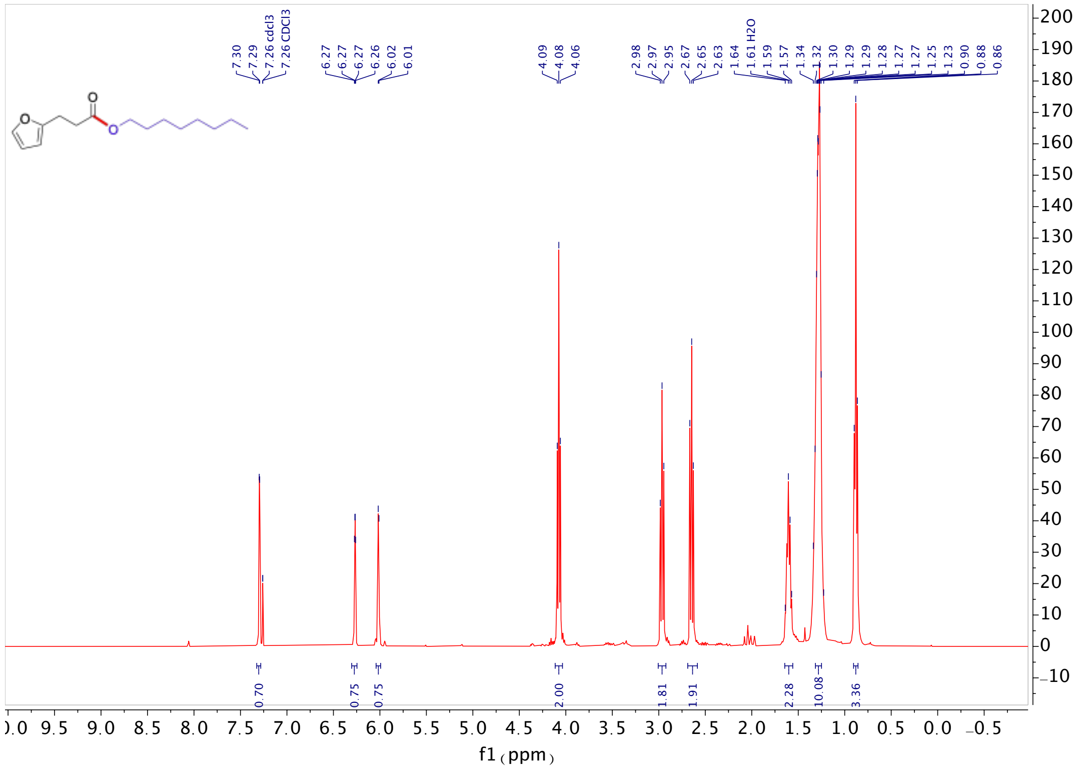

^{1}H NMR spectrum of *n*-Octyl 3-(furan-2-yl)propanoate (**16**)

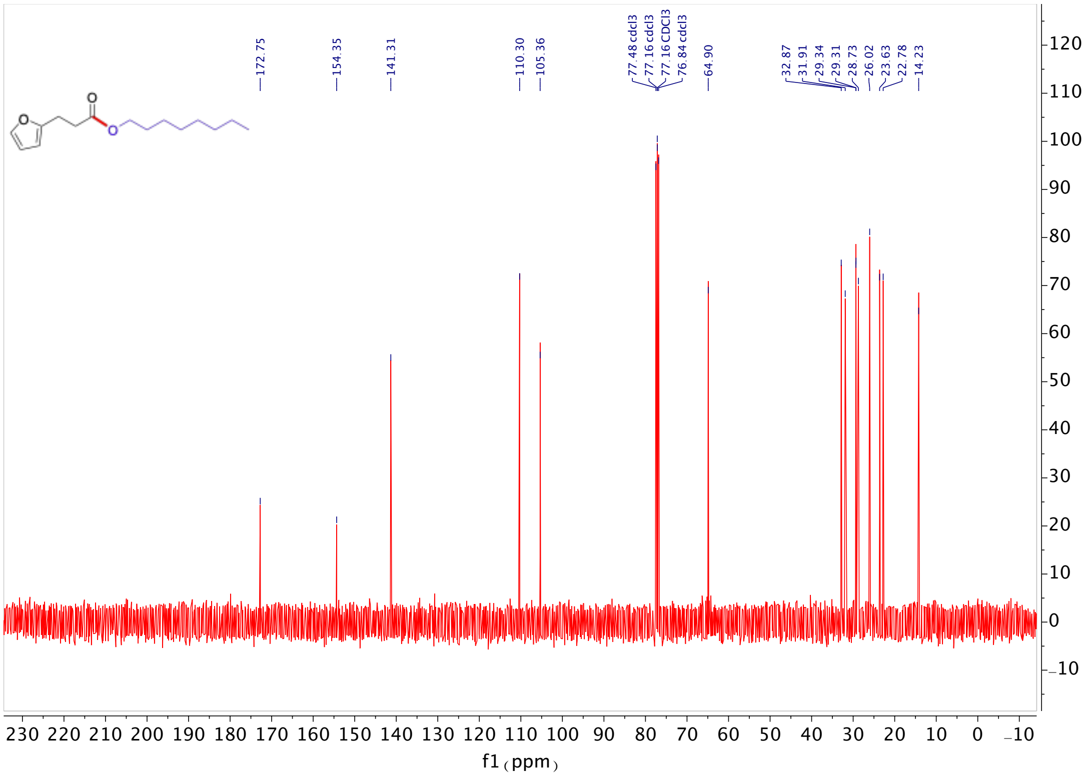

^{13}C NMR spectrum of *n*-Octyl 3-(furan-2-yl)propanoate (**16**)

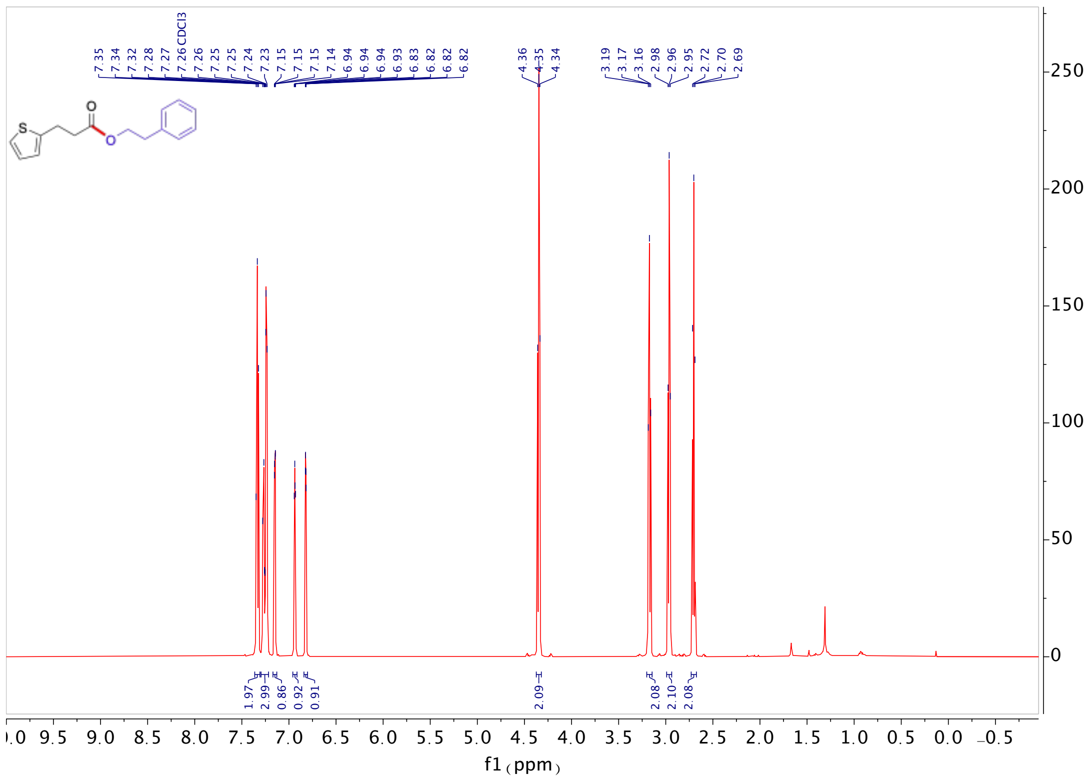

^{1}H NMR spectrum of Phenethyl 3-(thiophen-2-yl)propanoate (**19**)

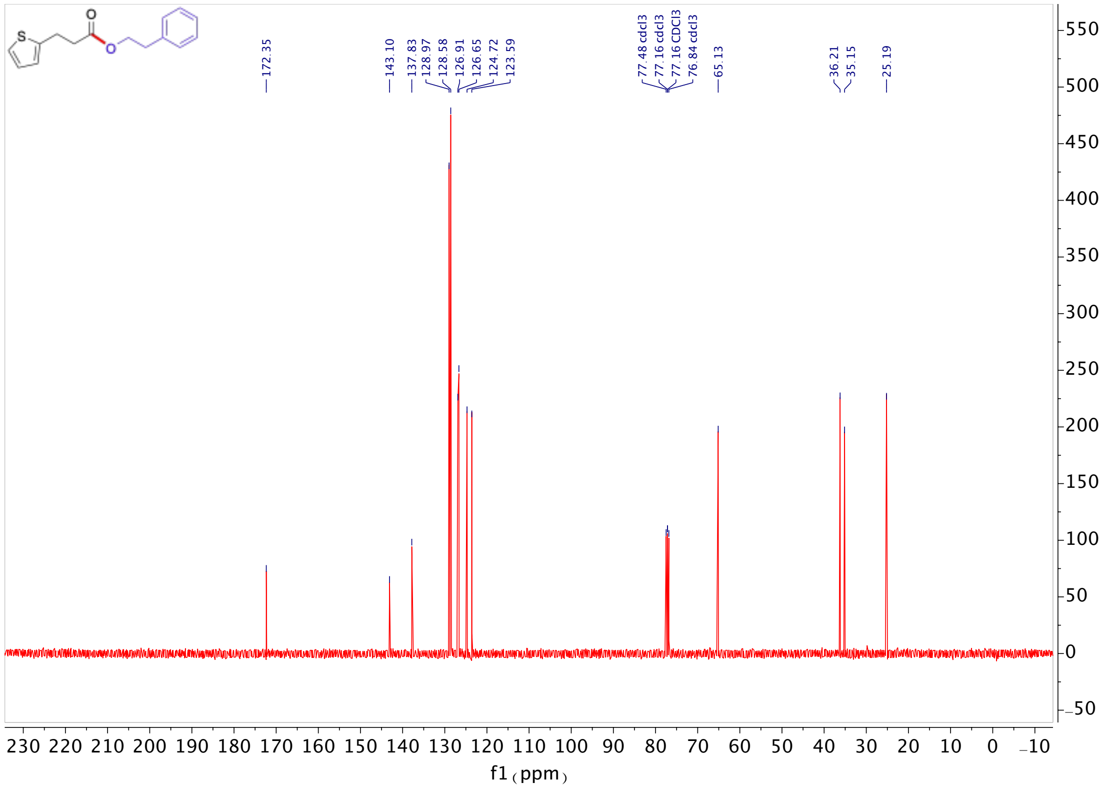

^{13}C NMR spectrum of Phenethyl 3-(thiophen-2-yl)propanoate (**19**)

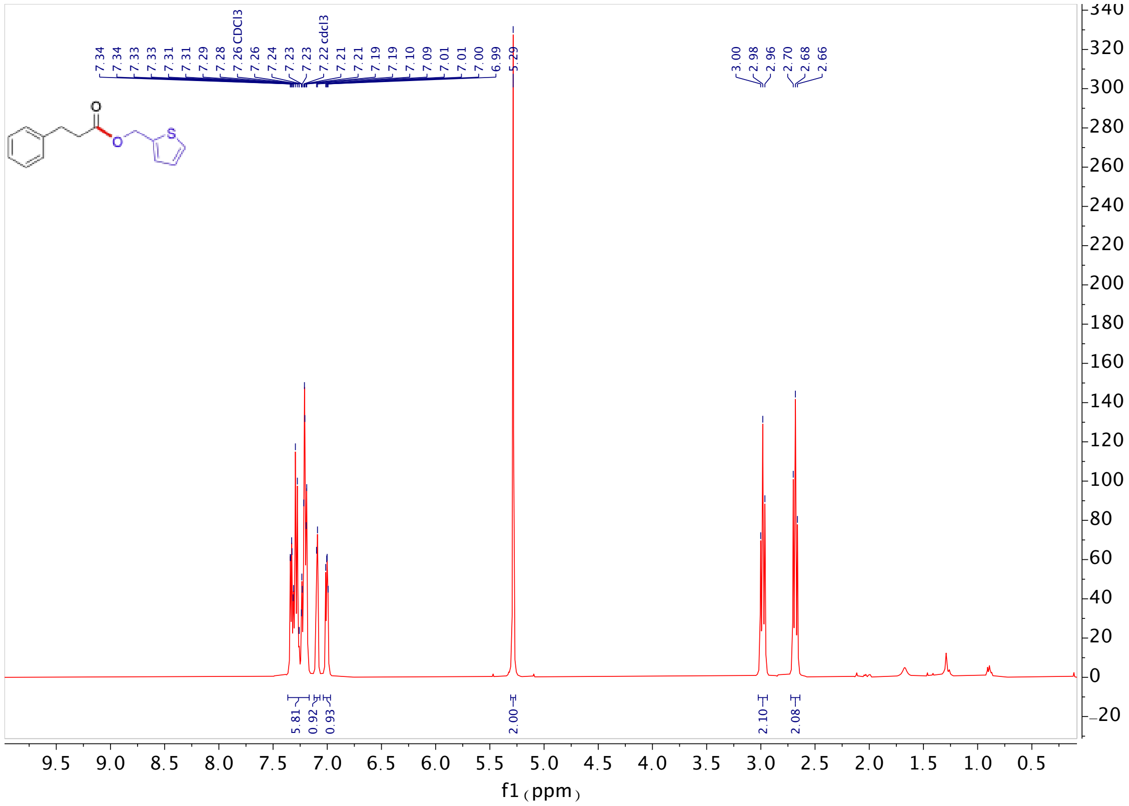

^{1}H NMR spectrum of Thiophen-2-ylmethyl 3-phenylpropanoate (**22**)

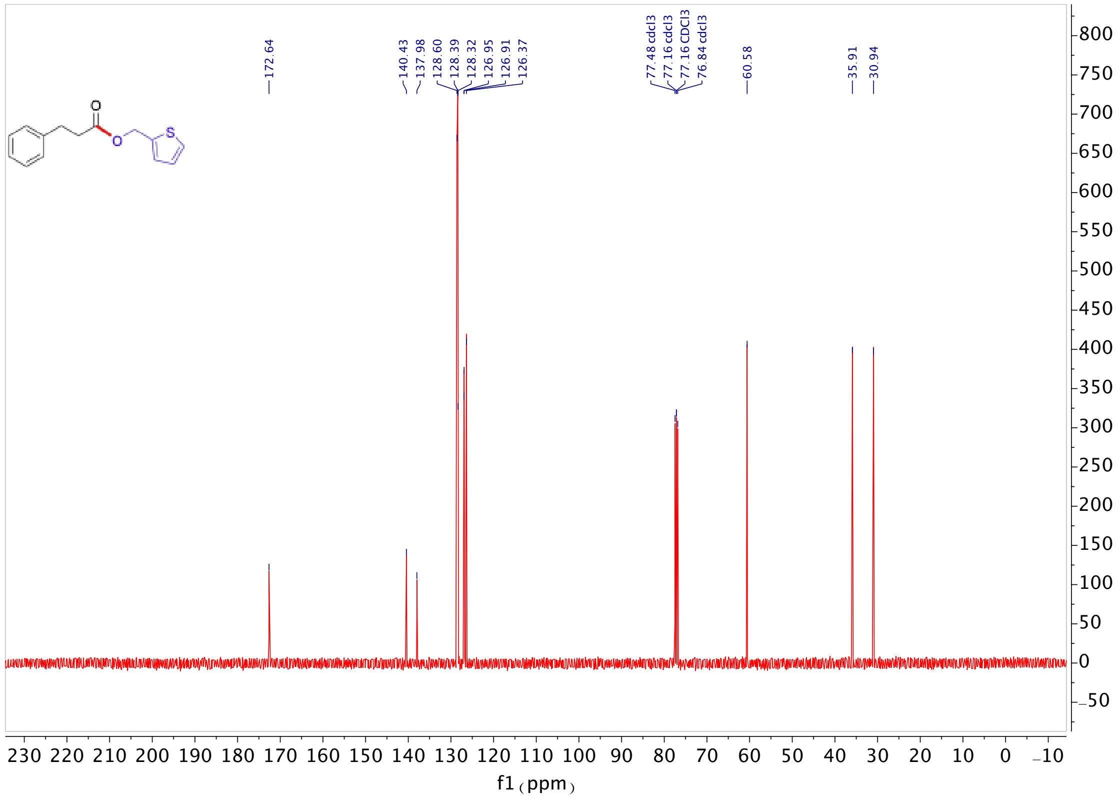

^{13}C NMR spectrum of Thiophen-2-ylmethyl 3-phenylpropanoate (**22**)

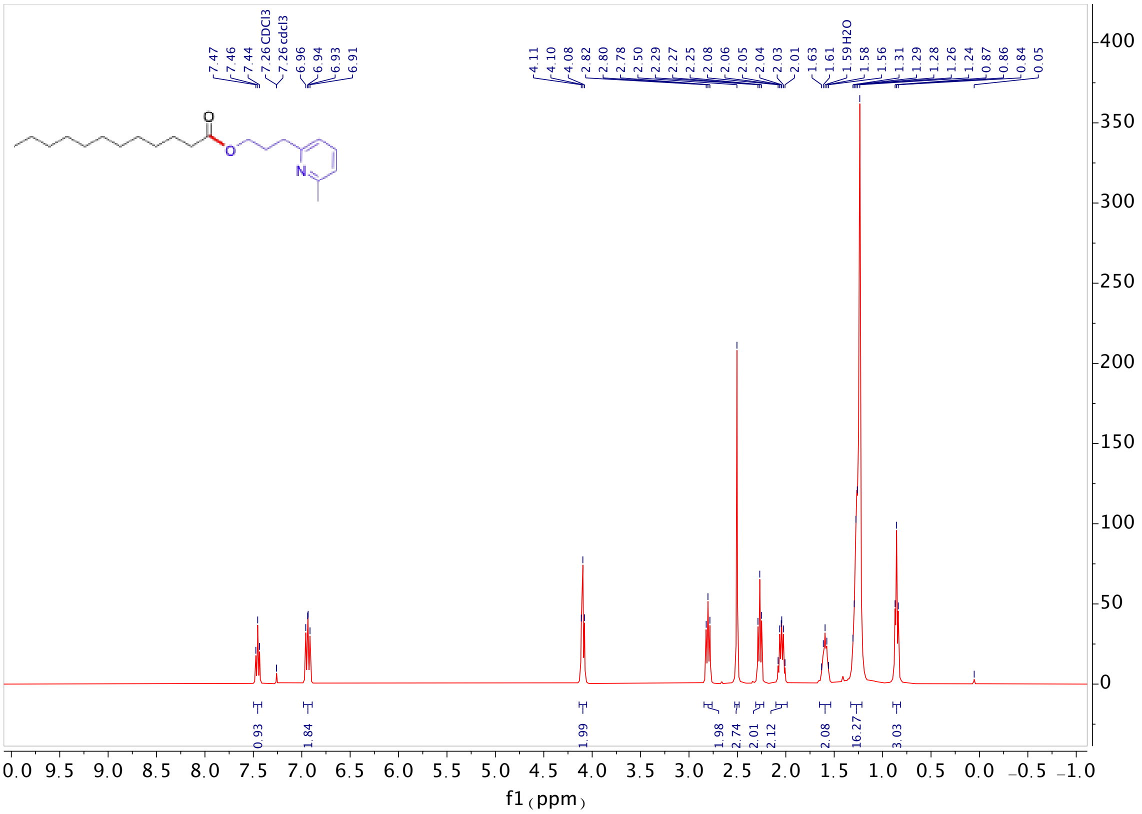

^{1}H NMR spectrum of 3-(6-Methylpyridin-2-yl)propyl dodecanoate (**23**)

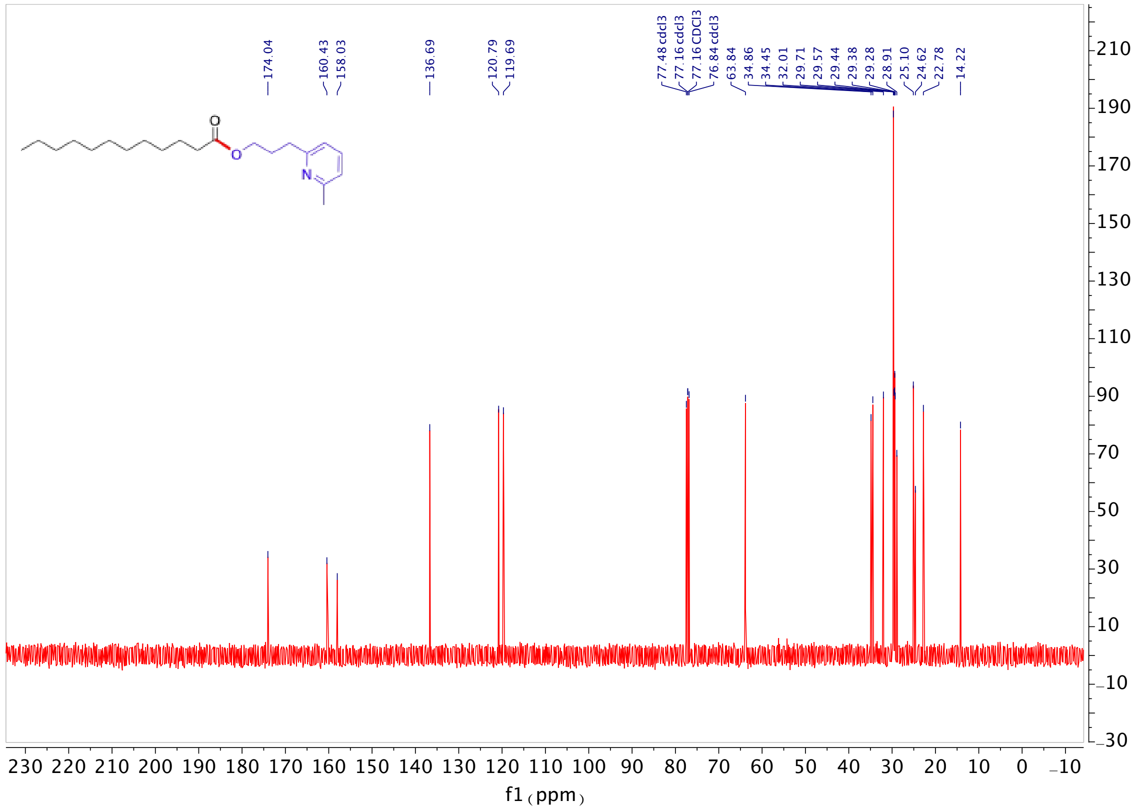

^{13}C NMR spectrum of 3-(6-Methylpyridin-2-yl)propyl dodecanoate (**23**)

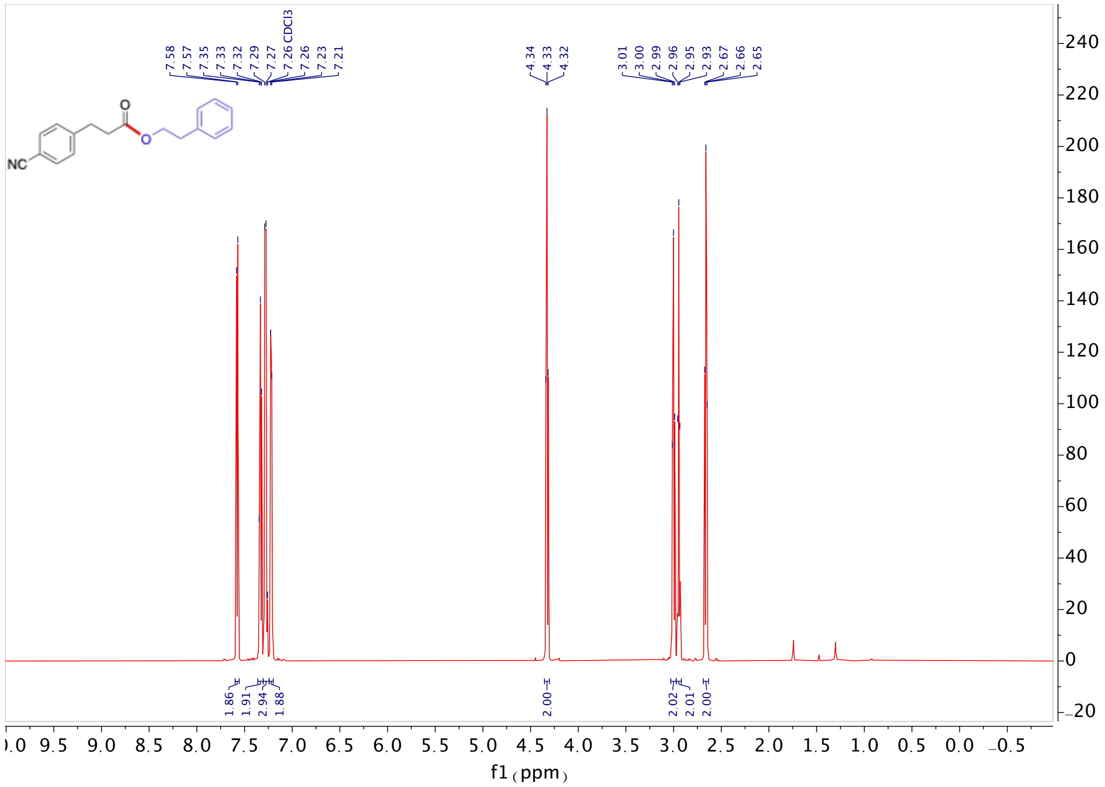

^{1}H NMR spectrum of Phenethyl 3-(4-cyanophenyl)propanoate (**24**)

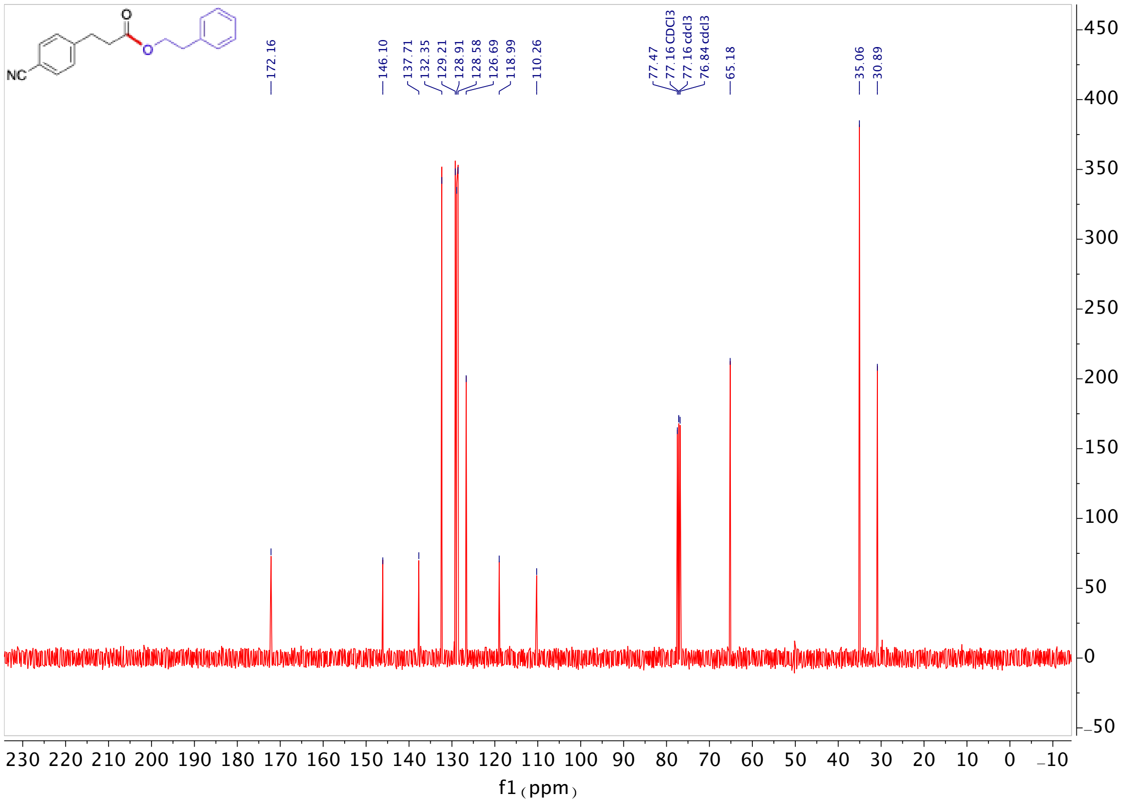

^{13}C NMR spectrum of Phenethyl 3-(4-cyanophenyl)propanoate (**24**)

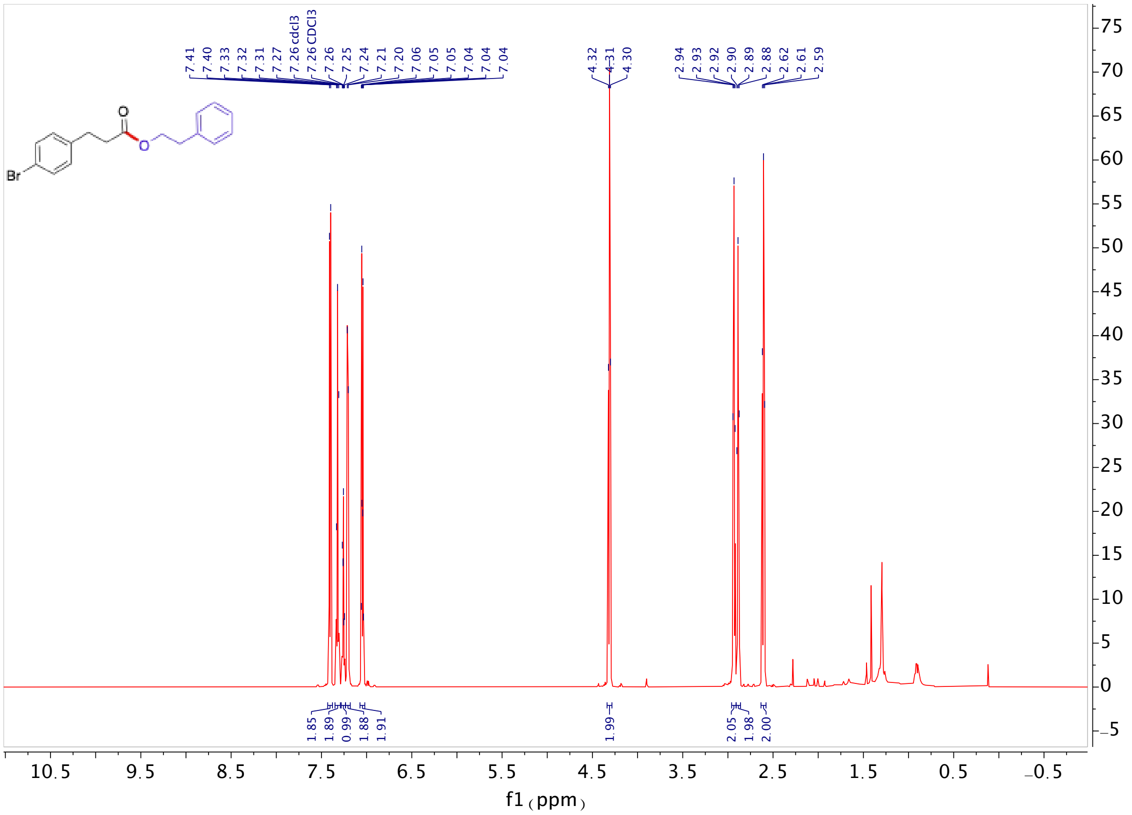

^{1}H NMR spectrum of Phenethyl 3-(4-bromophenyl)propanoate (**25**)

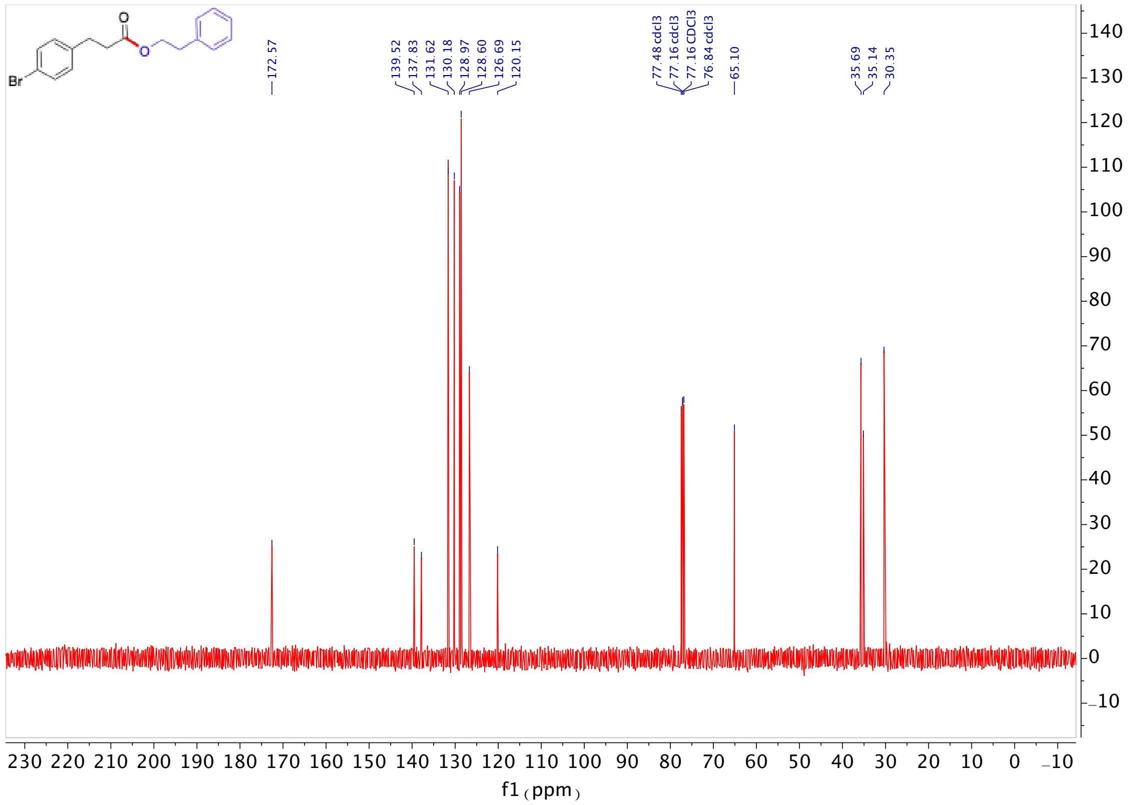

^{13}C NMR spectrum of Phenethyl 3-(4-bromophenyl)propanoate (**25**)

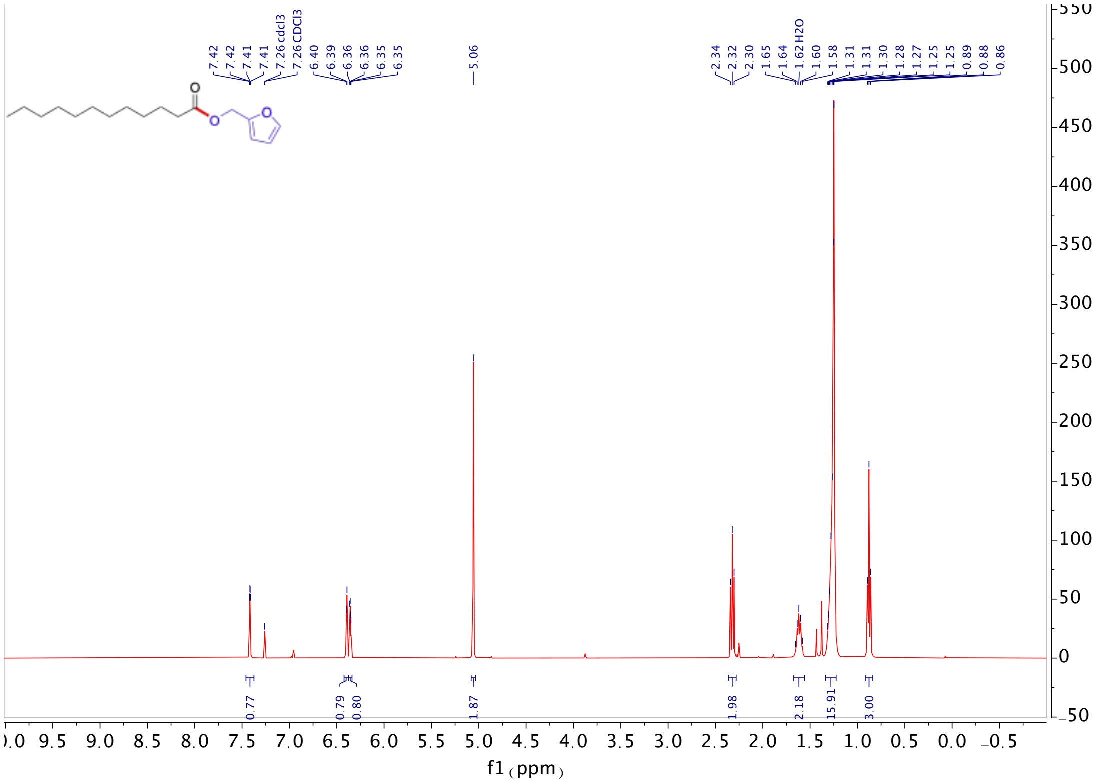

^1H NMR spectrum of Furan-2-ylmethyl dodecanoate (**26**)

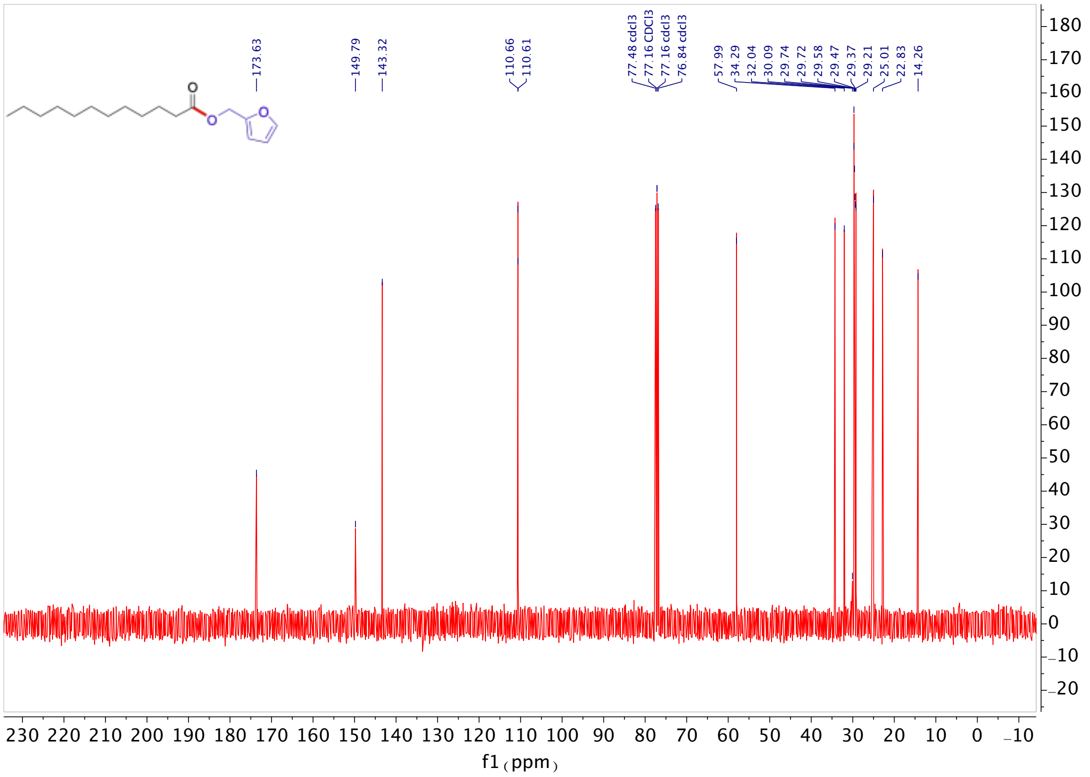

^{13}C NMR spectrum of Furan-2-ylmethyl dodecanoate (**26**)

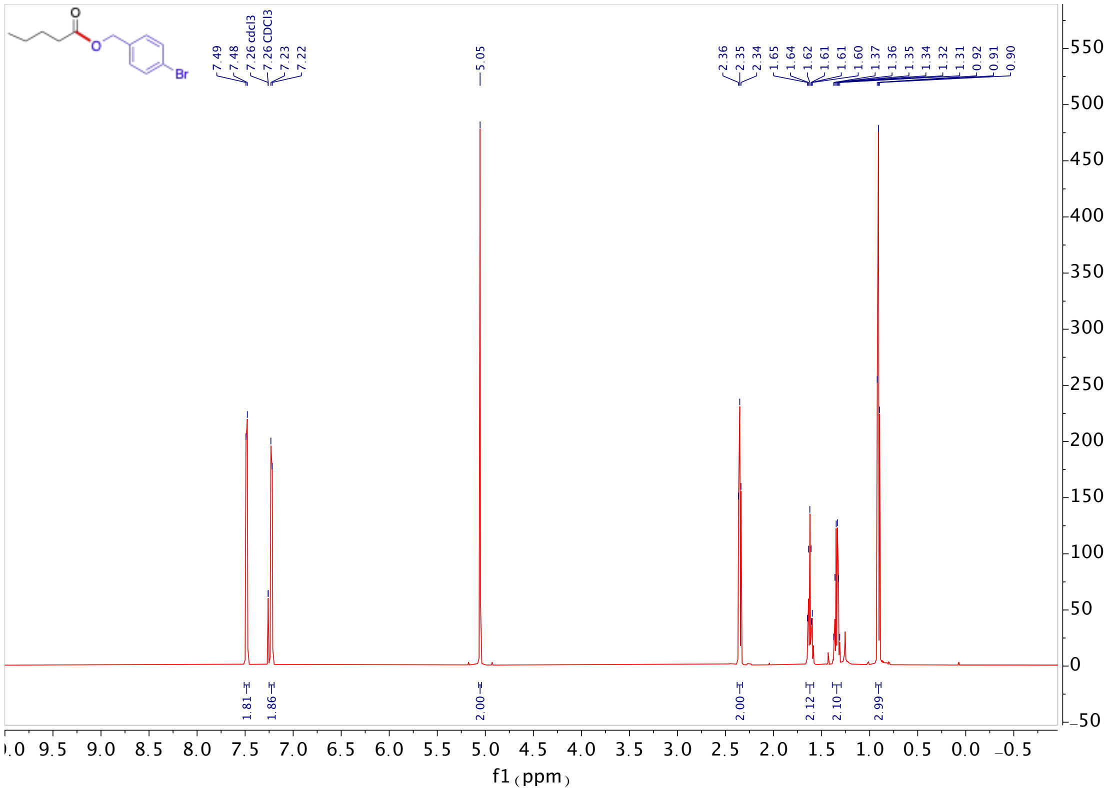

^{1}H NMR spectrum of 4-Bromobenzyl pentanoate (**27**)

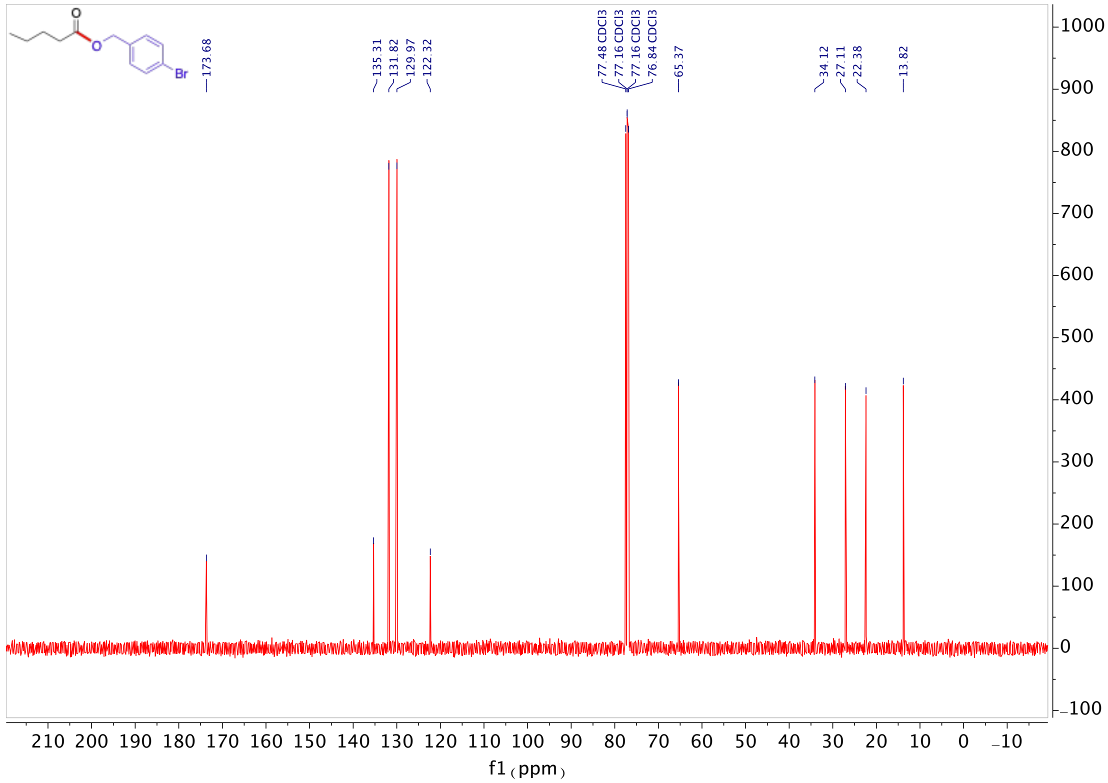

^{13}C NMR spectrum of 4-Bromobenzyl pentanoate (**27**)

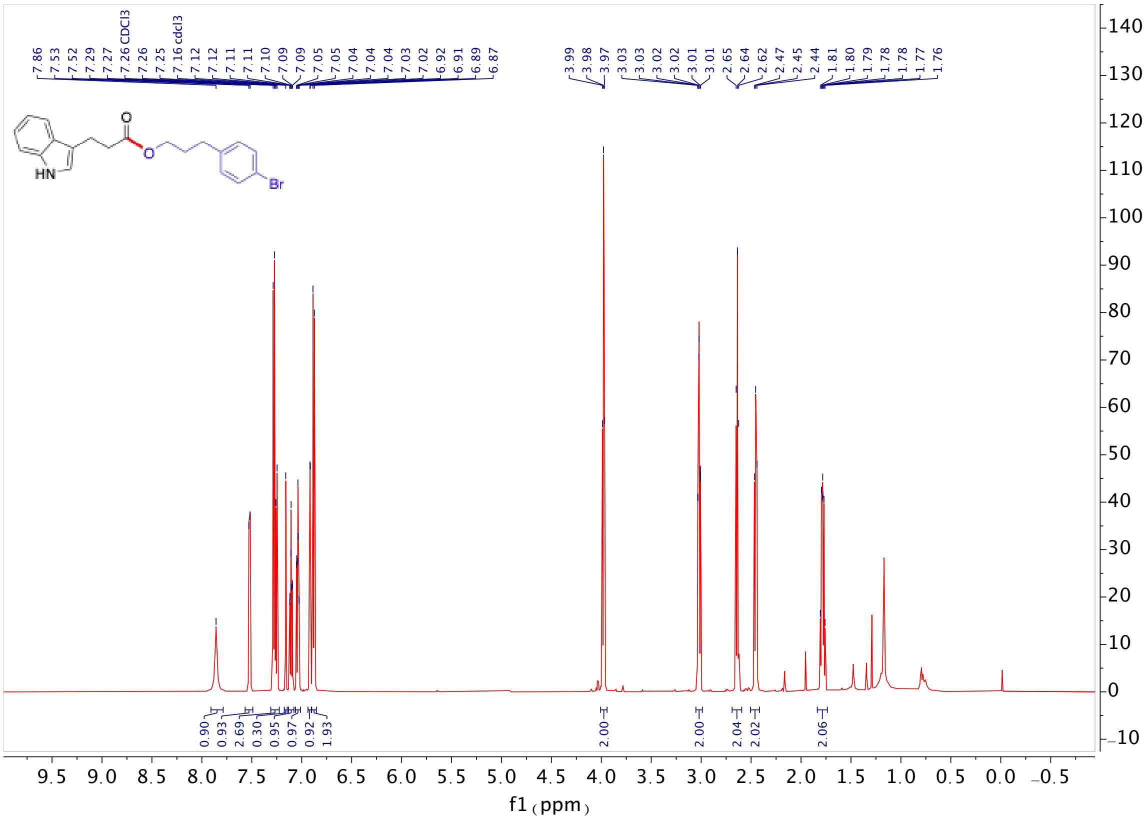

^{1}H NMR spectrum of 3-(4-Bromophenyl)propyl 3-(1H-indol-3-yl)propanoate (**28**)

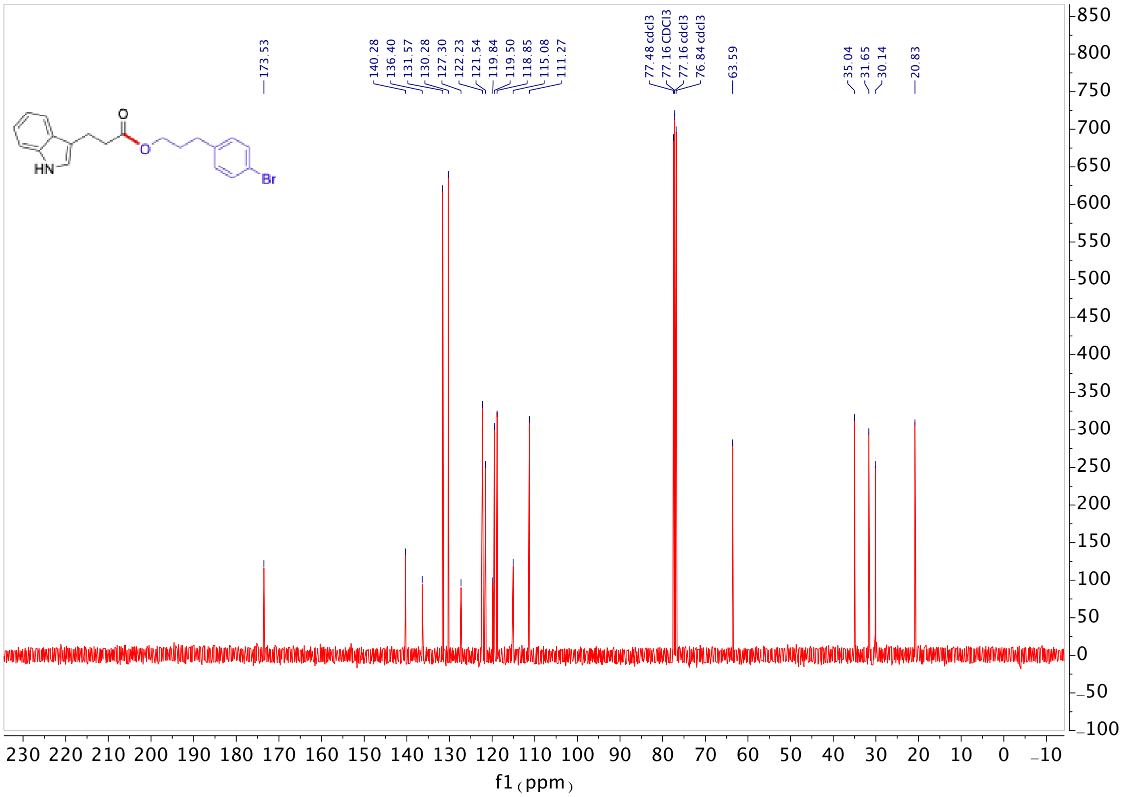

^{13}C NMR spectrum of 3-(4-Bromophenyl)propyl 3-(1H-indol-3-yl)propanoate (**28**)

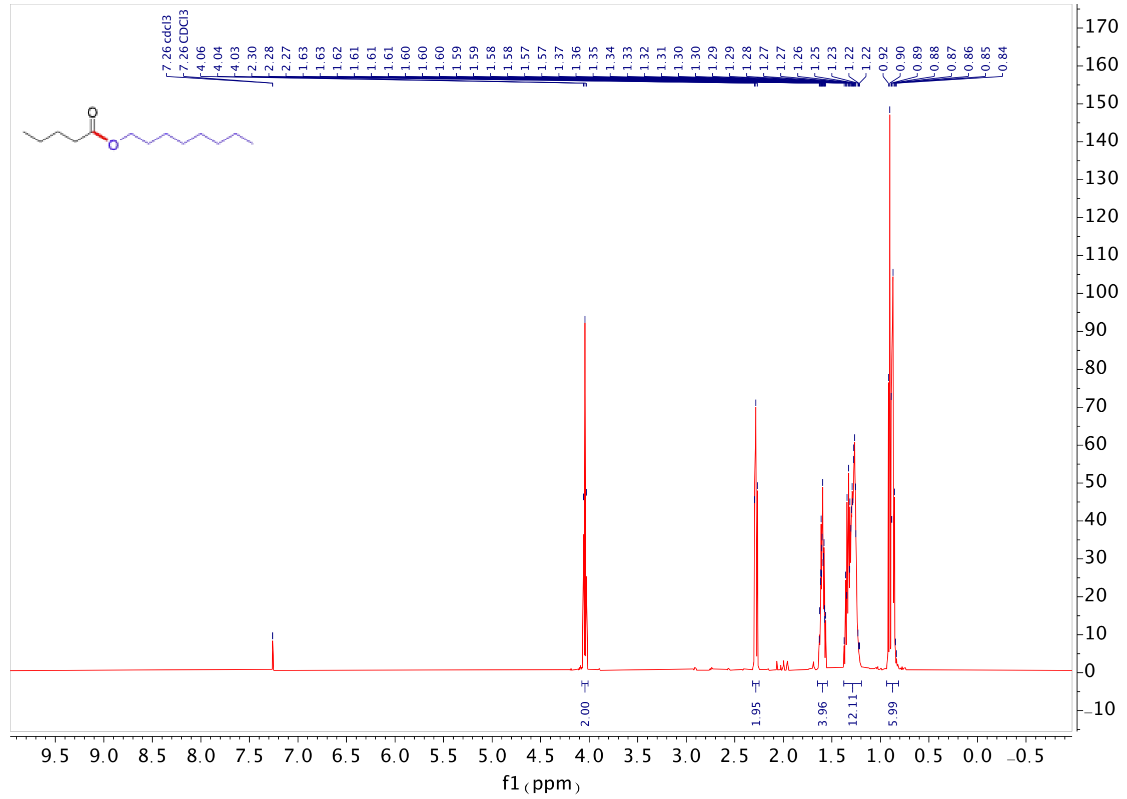

^{1}H NMR spectrum of *n*-Octyl pentanoate (**33**)

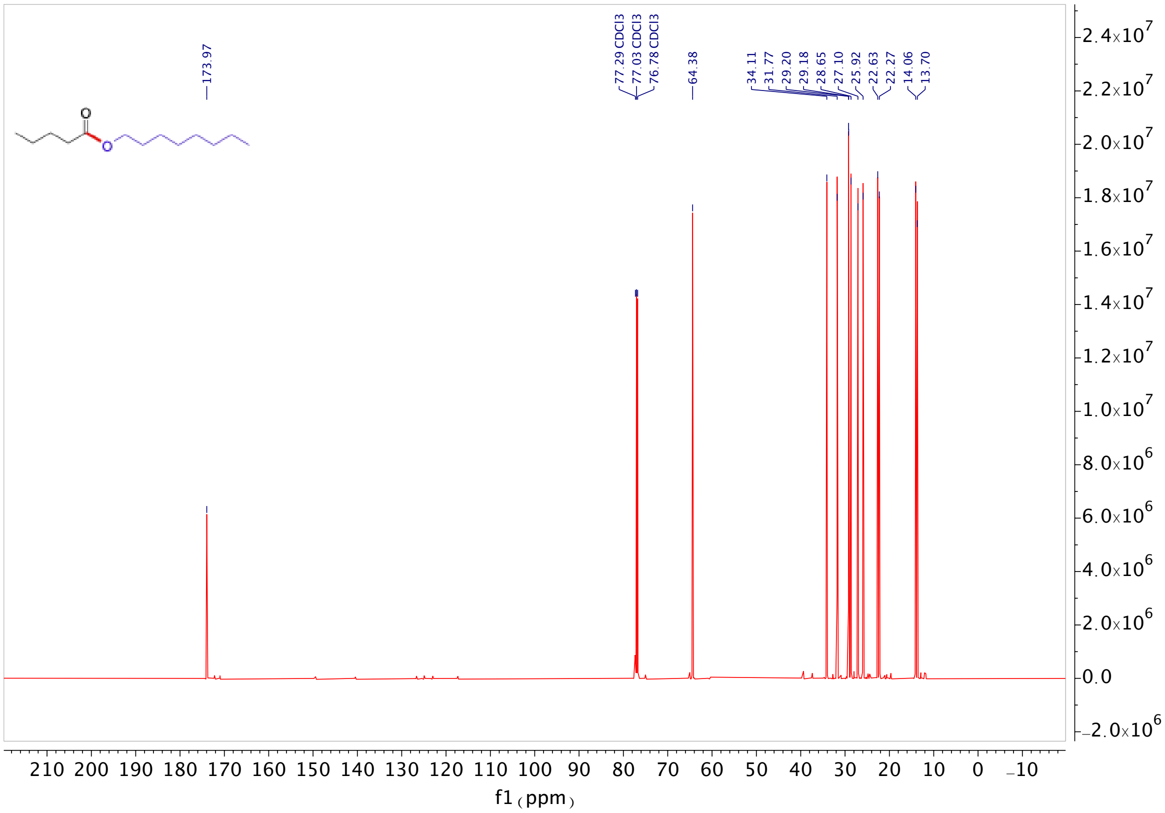

^{13}C NMR spectrum of *n*-Octyl pentanoate (**33**)

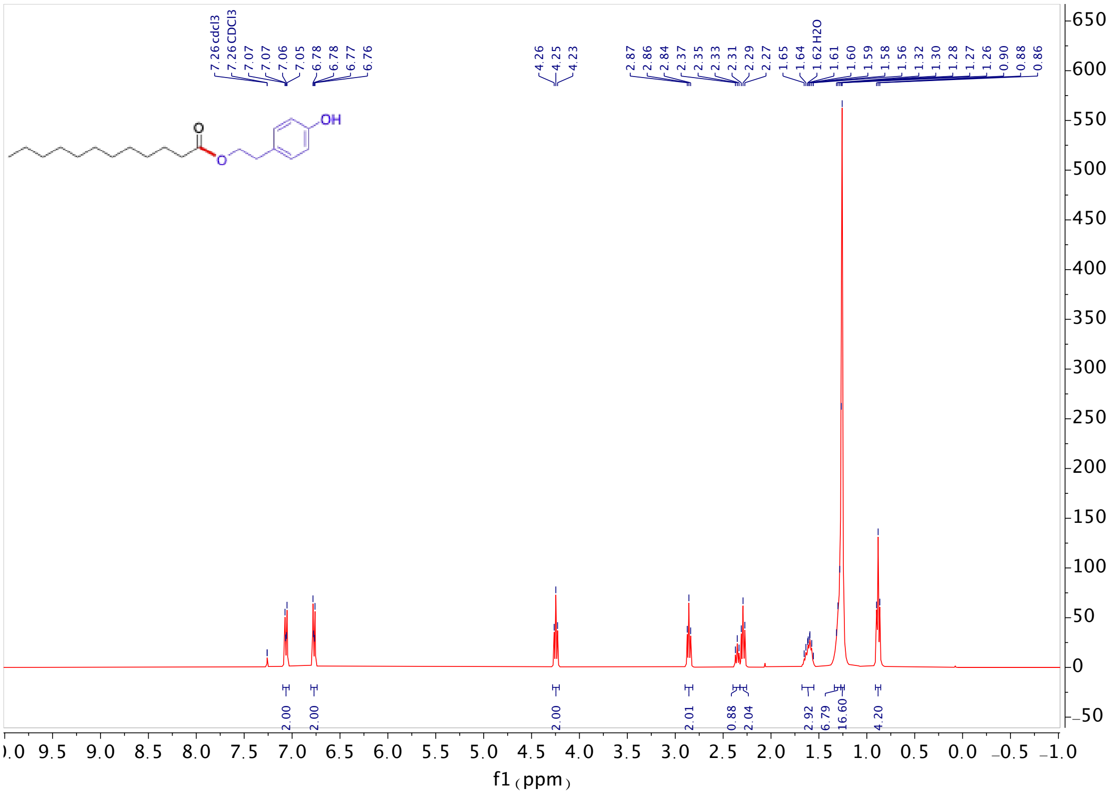

^{1}H NMR spectrum of 4-Hydroxyphenethyl dodecanoate (**36**)

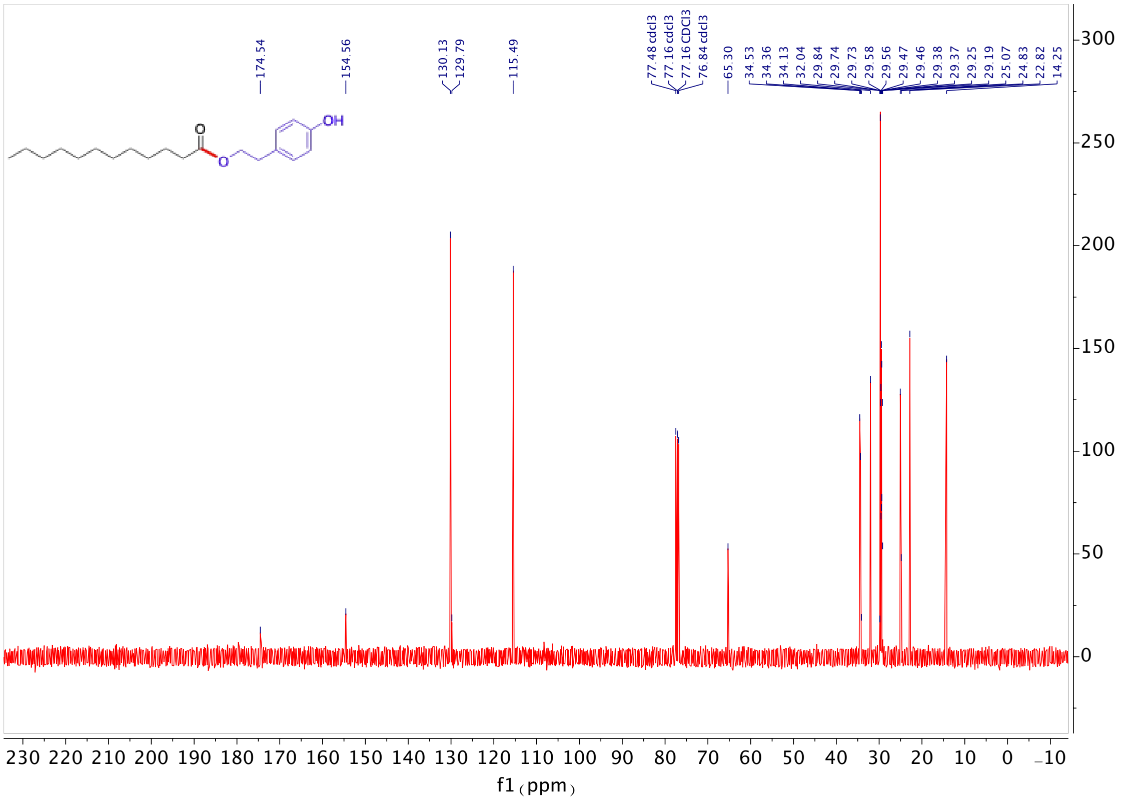

^{13}C NMR spectrum of 4-Hydroxyphenethyl dodecanoate (**36**)

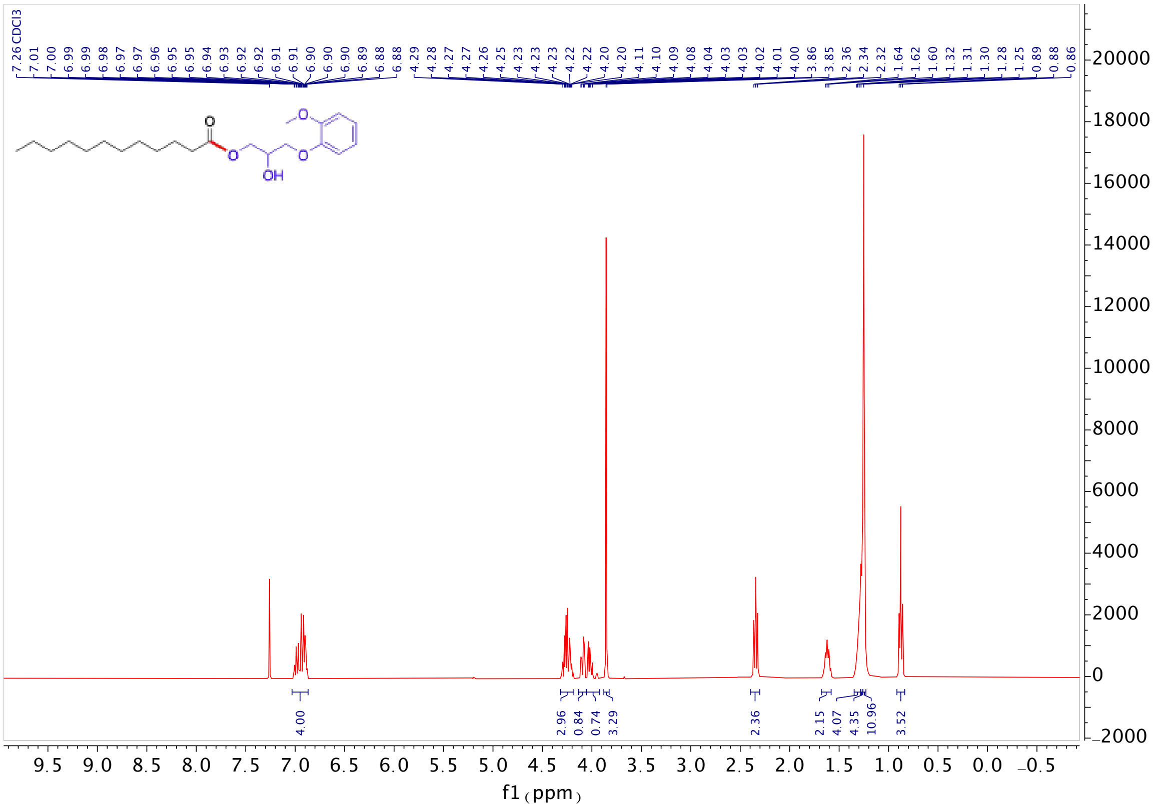

^1H NMR spectrum of 2-Hydroxy-3-(2-methoxyphenoxy)propyl dodecanoate (**38**)

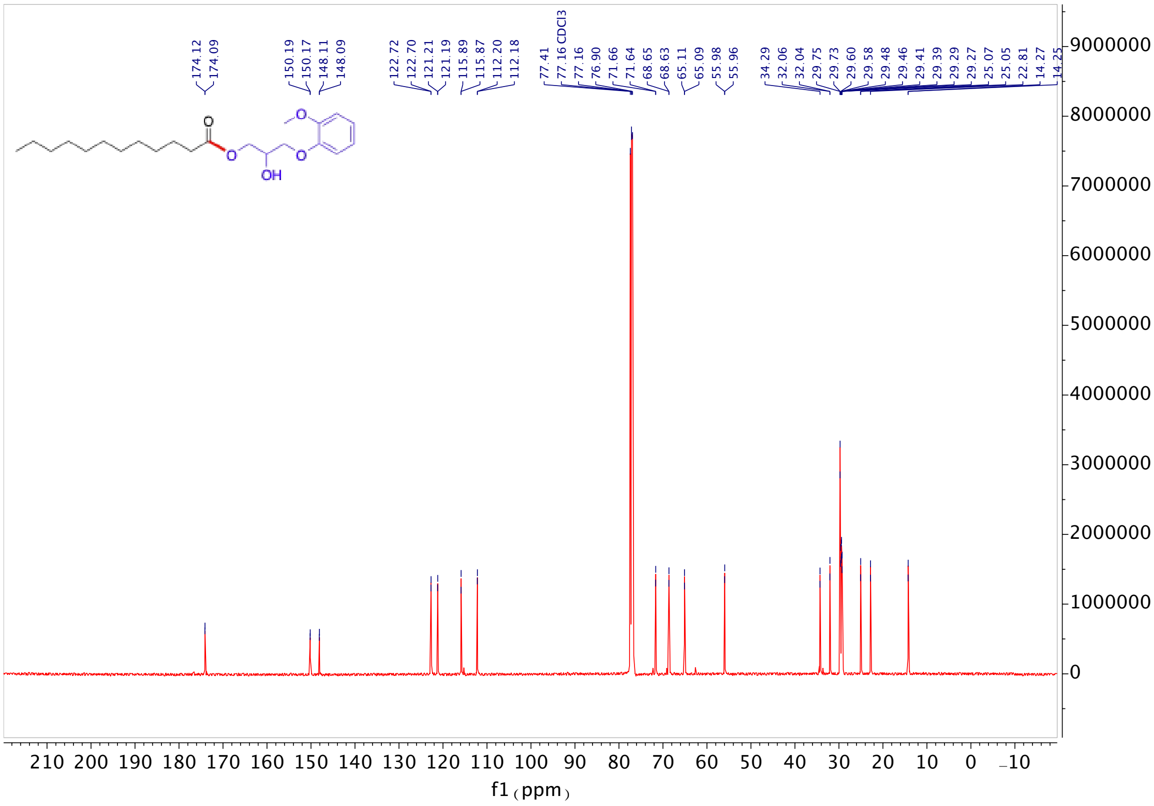

^{13}C NMR spectrum of 2-Hydroxy-3-(2-methoxyphenoxy)propyl dodecanoate (**38**)

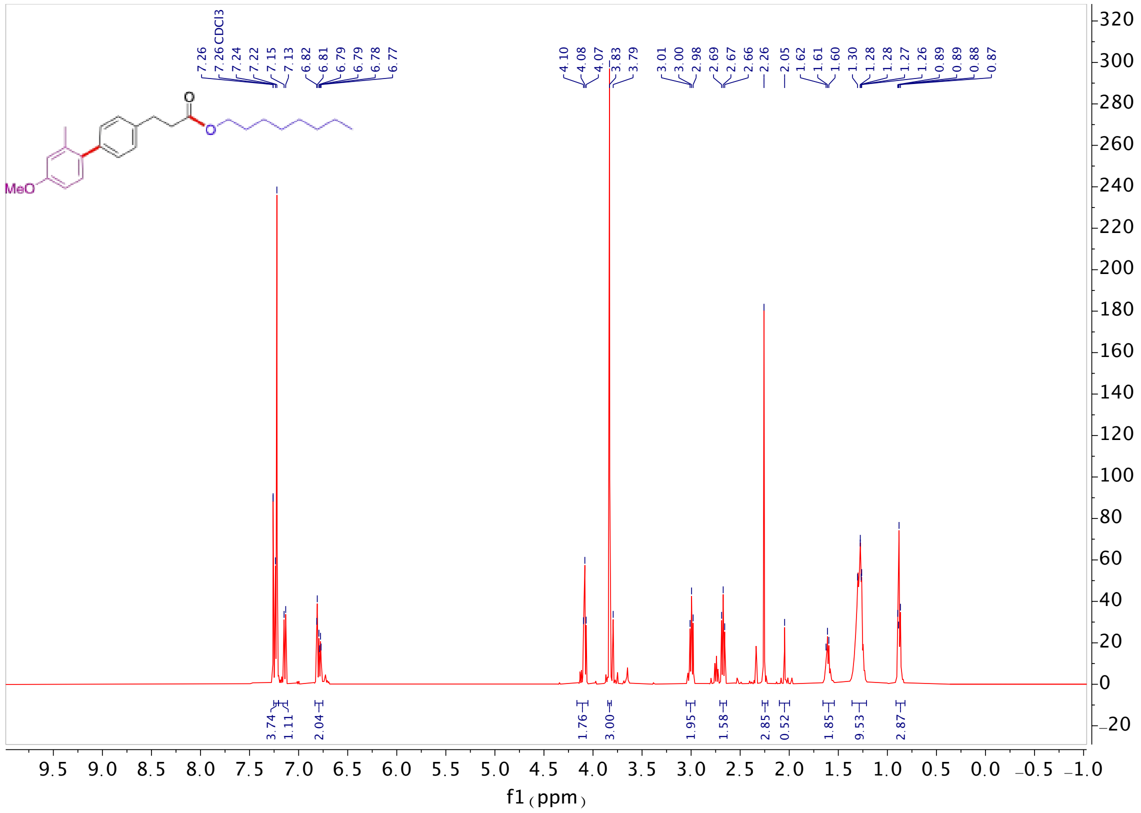

^{1}H NMR spectrum of Octyl 3-(4'-methoxy-2'-methyl-[1,1'-biphenyl]-4-yl)propanoate (**43**)

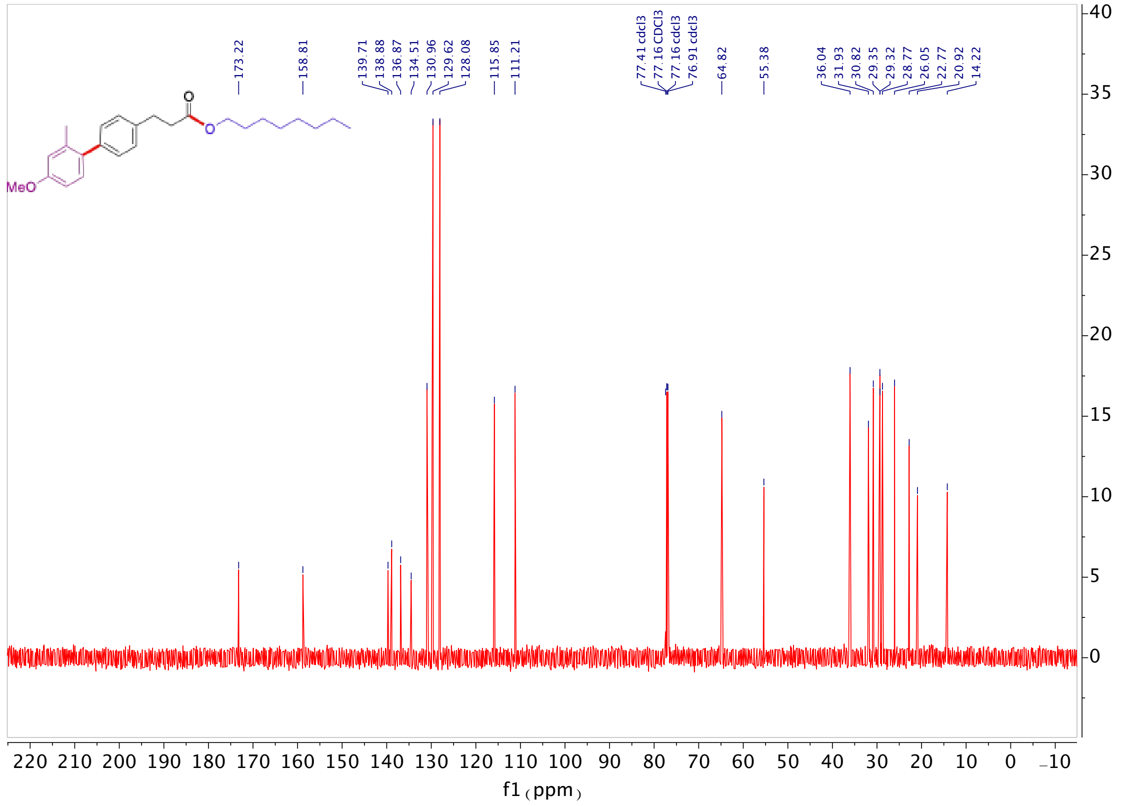

^{13}C NMR spectrum of Octyl 3-(4'-methoxy-2'-methyl-[1,1'-biphenyl]-4-yl)propanoate (**43**)

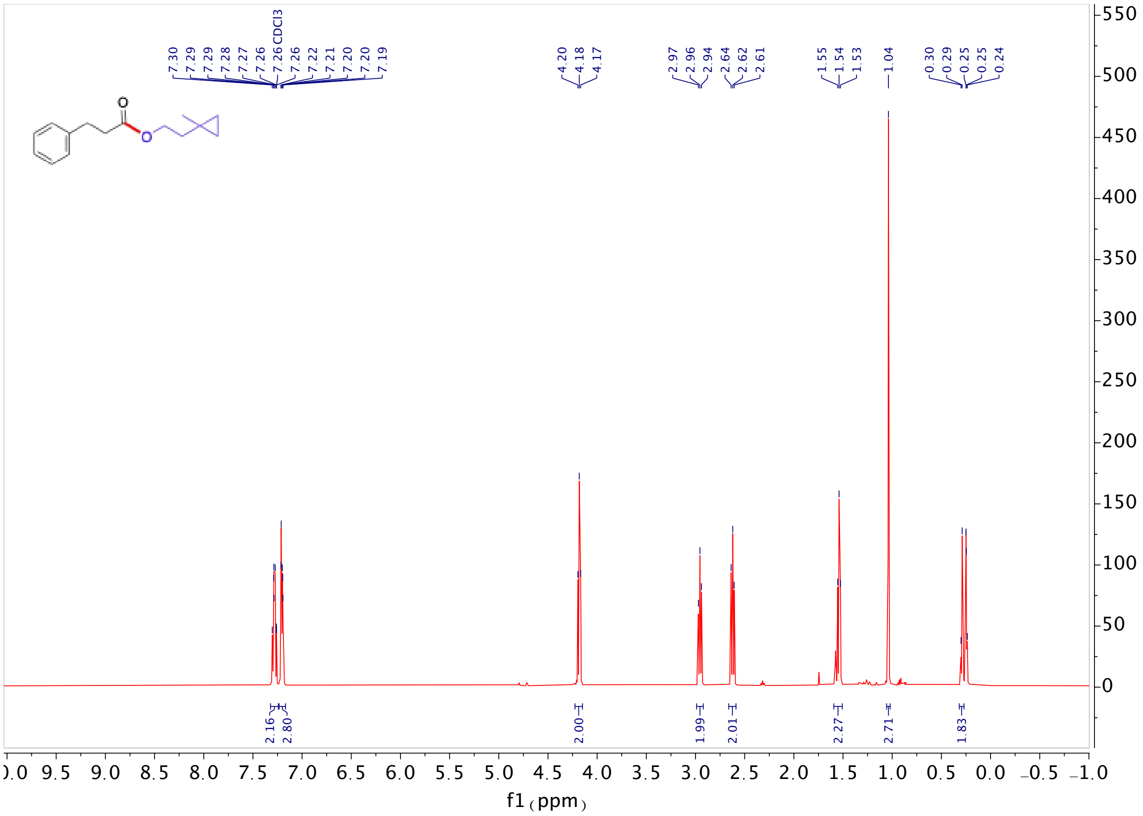

^{1}H NMR spectrum of 2-(1-Methylcyclopropyl)ethyl 3-phenylpropanoate (**47**)

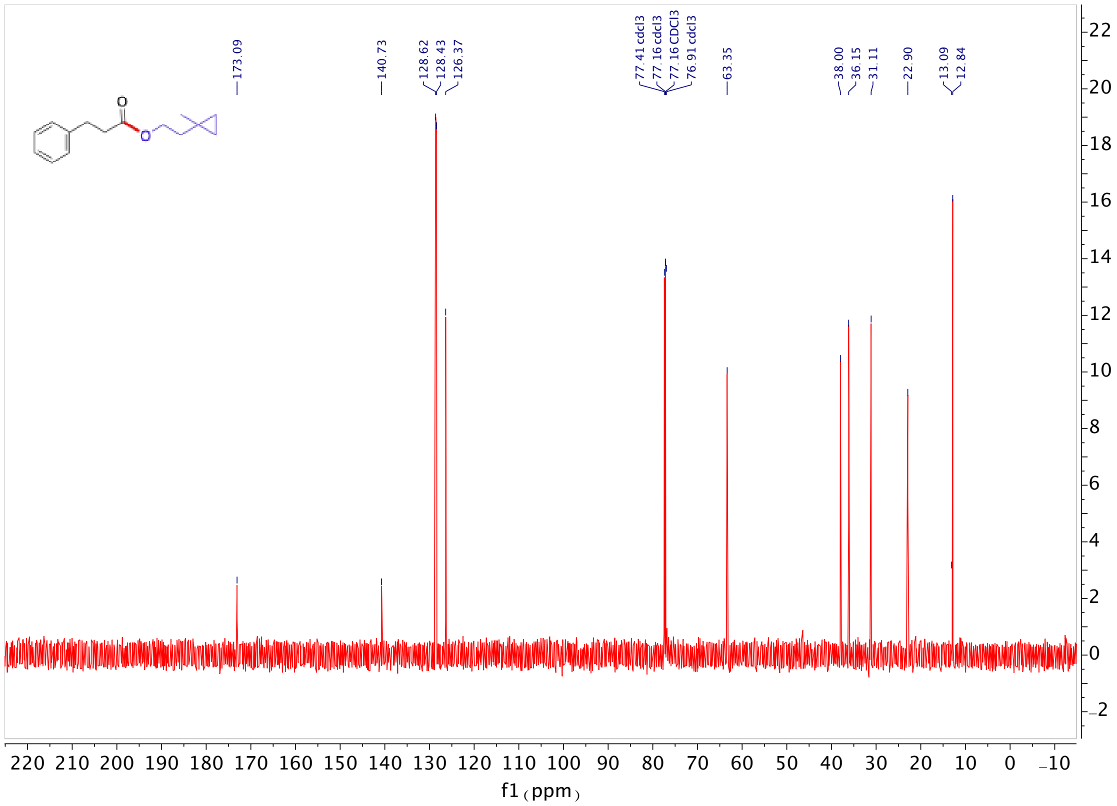

^{13}C NMR spectrum of2-(1-Methylcyclopropyl)ethyl 3-phenylpropanoate (**47**)

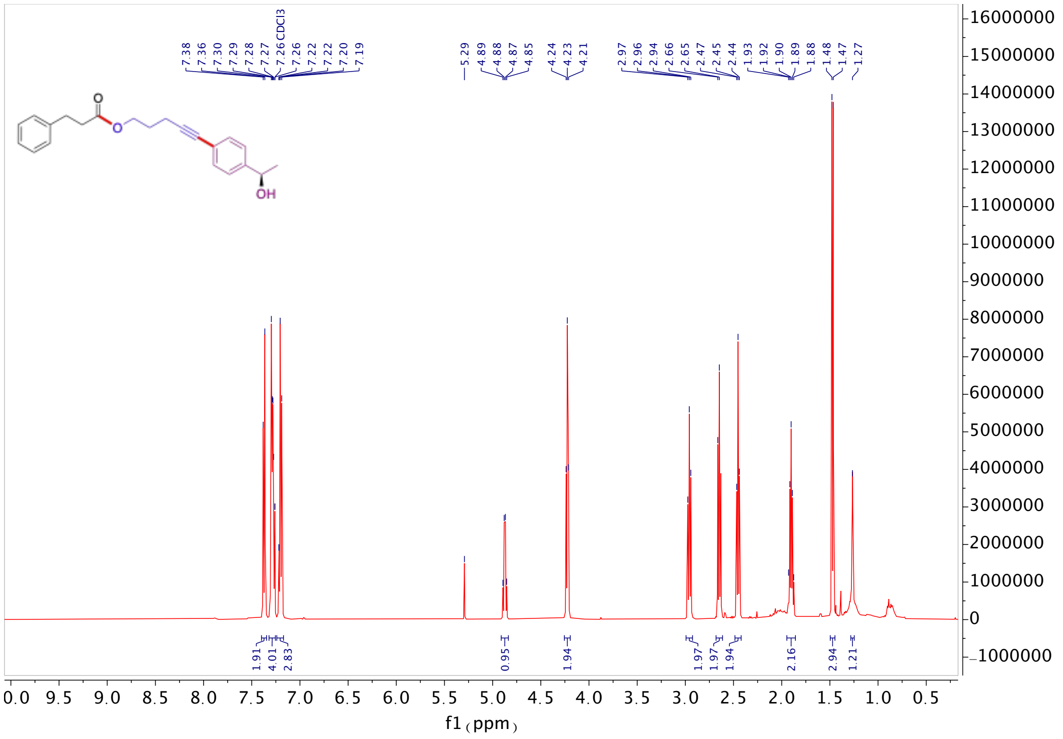

^{1}H NMR spectrum of (*R*)-5-(4-(1-Hydroxyethyl)phenyl)pent-4-yn-1-yl 3-phenylpropanoate (**52**)

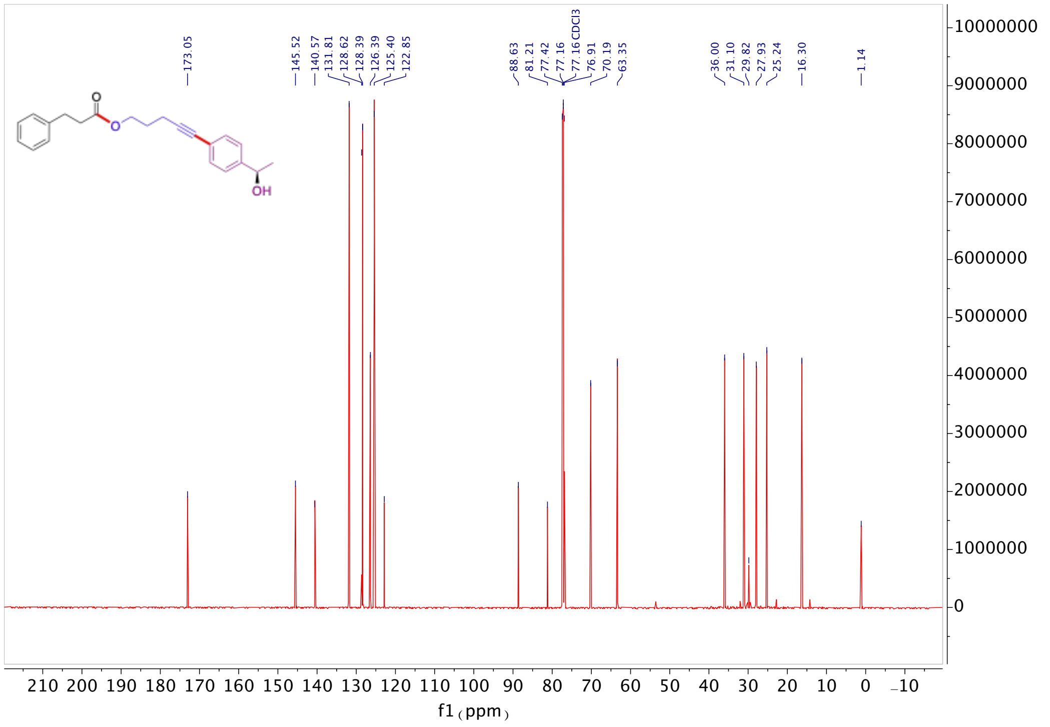

^{13}C NMR spectrum of(*R*)-5-(4-(1-Hydroxyethyl)phenyl)pent-4-yn-1-yl 3-phenylpropanoate (**52**)

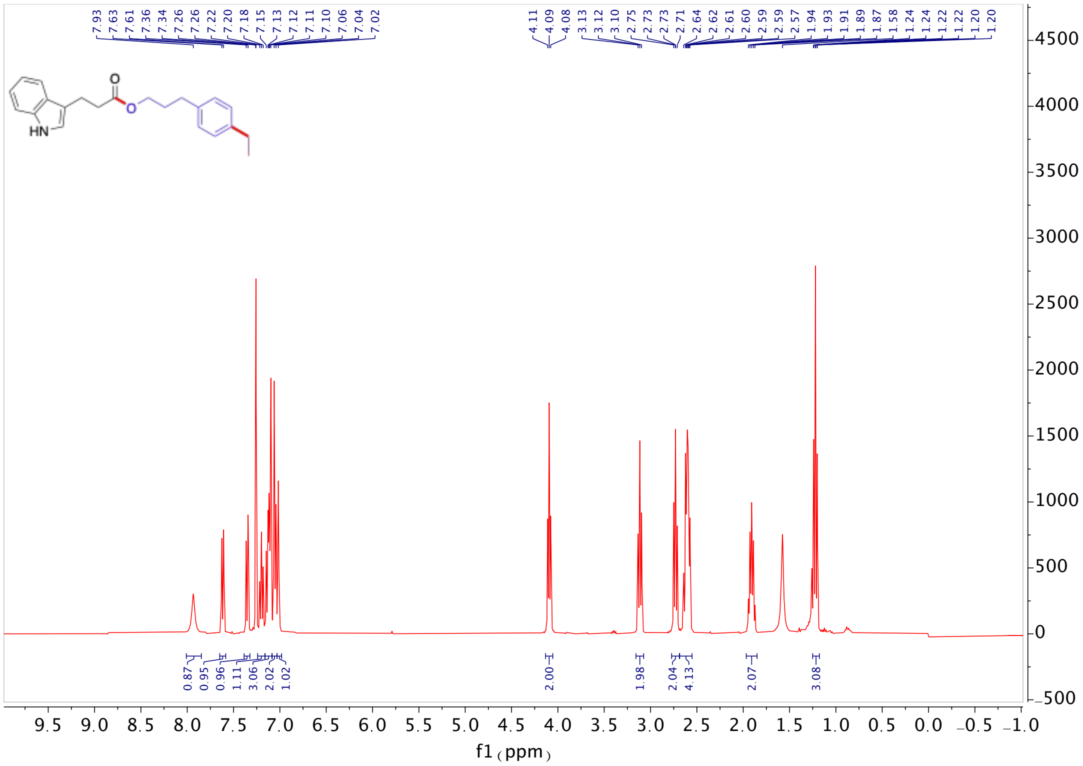

^{1}H NMR spectrum of 3-(4-Ethylphenyl)propyl 3-(1H-indol-3-yl)propanoate (**56**)

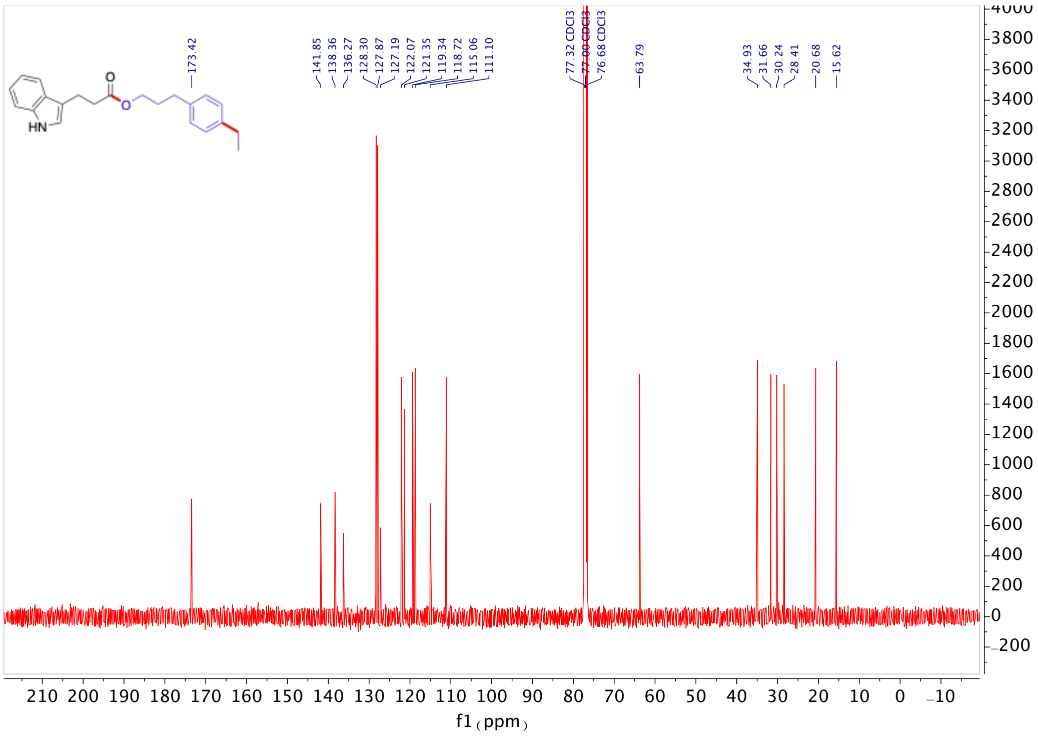

^{13}C NMR spectrum of 3-(4-Ethylphenyl)propyl 3-(1H-indol-3-yl)propanoate (**56**)
